# Combined BET bromodomain and DNA methyltransferase inhibition targets critical survival pathways in transdifferentiated prostate cancer

**DOI:** 10.1172/jci.insight.207543

**Published:** 2026-08-11

**Authors:** William K. Storck, Diana Flores, Anbarasu Kumaraswamy, Zhi Duan, Shrabastee Chakraborty, Chao Zhang, Eva Rodansky, Dhruv Khokhani, Olivia A. Swaim, Karan Bedi, Raymond G. Cavalcante, Canping Chen, Faming Zhao, Ya-Mei Hu, Zheng Xia, Ryan J. Rebernick, Marcin Cieslik, Rahul Mannan, Somnath Mahapatra, Arul M. Chinnaiyan, Aaron M. Udager, Joshua A. Kuleape, Catherine R. Alumkal, Hannah N. Beck, Peter S. Nelson, Colm Morrissey, Michael C. Haffner, Leigh Ellis, Yuzhuo Wang, Joel A. Yates, Joshi J. Alumkal

**Affiliations:** 1 Department of Internal Medicine,; 2 Rogel Cancer Center,; 3 College of Literature, Science, and the Arts,; 4 Department of Biostatistics, School of Public Health, and; 5 Bioinformatics Core, University of Michigan, Ann Arbor, Michigan, USA.; 6 Department of Biomedical Engineering and; 7 Knight Cancer Institute, Oregon Health & Science University, Portland, Oregon, USA.; 8 Michigan Center for Translational Pathology,; 9 Department of Computational Medicine and Bioinformatics,; 10 Department of Pathology,; 11 Howard Hughes Medical Institute, and; 12 Department of Urology, University of Michigan, Ann Arbor, Michigan, USA.; 13 Department of Urology, University of Washington, Seattle, Washington, USA.; 14 Division of Human Biology and; 15 Division of Clinical Research, Fred Hutchinson Cancer Center, Seattle, Washington, USA.; 16 Department of Laboratory Medicine and Pathology, University of Washington, Seattle, Washington, USA.; 17 Center for Prostate Disease Research, Murtha Cancer Center Research Program, Department of Surgery, Uniformed Services University of the Health Sciences, Bethesda, Maryland, USA.; 18 The Henry M. Jackson Foundation for the Advancement of Military Medicine, Inc., Bethesda, Maryland, USA.; 19 Genitourinary Malignancies Branch, Center for Cancer Research, National Cancer Institute, Bethesda, Maryland, USA.; 20 Vancouver Prostate Centre, Vancouver, British Columbia, Canada.; 21 Department of Urologic Sciences, Faculty of Medicine, University of British Columbia, Vancouver, British Columbia, Canada.; 22 BC Cancer Research Institute, BC Cancer, Vancouver, British Columbia, Canada.

**Keywords:** Cell biology, Oncology, Drug therapy, Epigenetics, Prostate cancer

## Abstract

Lineage plasticity, or transdifferentiation, is increasingly recognized as a resistance mechanism to androgen receptor (AR) inhibition in prostate cancer. Lineage plasticity is characterized by loss of AR signaling and epithelial differentiation, along with activation of stemness-associated pathways, epithelial-mesenchymal transition, or alternative differentiation programs such as neuroendocrine prostate cancer (NEPC). Loss of the tumor suppressors *TP53* and *RB1* is common in tumors exhibiting lineage plasticity; however, the mechanisms by which *TP53*/*RB1* loss promotes this phenotype remain poorly understood, and effective treatments are limited. Using multiomic profiling of *TP53*/*RB1*-loss prostate cancer models, we identified alterations in chromatin accessibility, DNA methylation, and gene expression associated with lineage plasticity. Importantly, many pathways activated upon *TP53*/*RB1* loss could be blocked through BET bromodomain inhibition. *TP53*/*RB1*-deficient cells also harbored widespread DNA methylation changes that silenced pathways linked with restraining lineage plasticity. Combined BET bromodomain and DNA methyltransferase (DNMT) inhibition was more effective than single-agent treatment in suppressing growth of *TP53*/*RB1*-loss models exhibiting a stem-like or NEPC program. This was partly explained by abrogation of discrete lineage plasticity pathways modulated by each agent. Altogether, our work suggests combined BET bromodomain and DNMT inhibition is a promising therapeutic approach for prostate tumors exhibiting lineage plasticity.

## Abstract 2

Some prostate cancers undergo lineage plasticity, or transdifferentiation, through epigenetic reprogramming. Combination BET bromodomain and DNMT inhibition is a promising treatment for these tumors.

## Graphical abstract

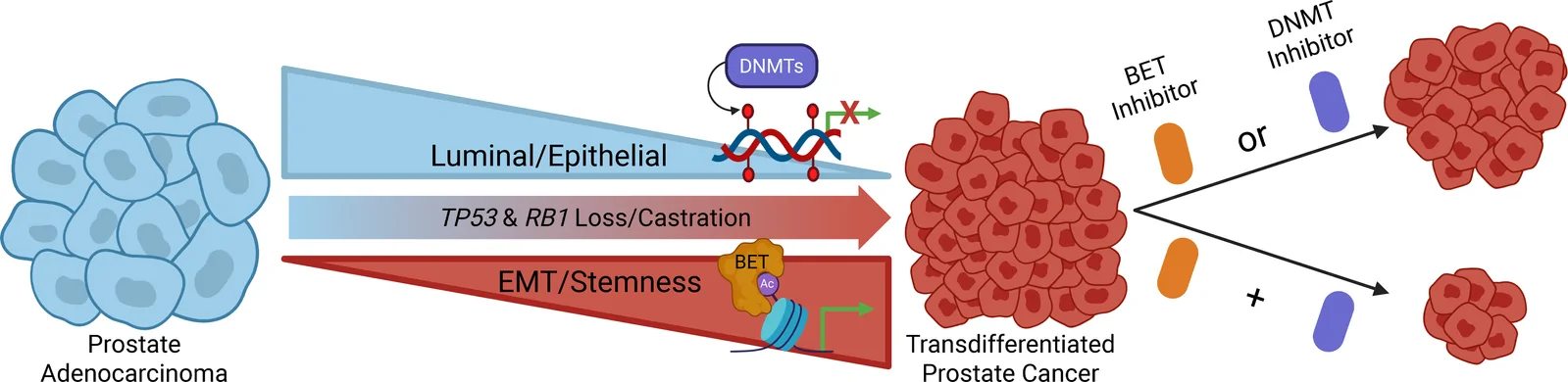

## Introduction

The androgen receptor (AR) is the primary therapeutic target in metastatic prostate cancer. Although AR pathway inhibitors are initially effective, resistance is nearly universal. Most resistant tumors still exhibit AR reliance upon progression. However, a subset loses AR reliance and undergoes treatment-induced lineage plasticity, also known as transdifferentiation (1, 2). Lineage plasticity is an adaptive process that enables cells to respond to environmental stressors (3) and drives treatment resistance, leading to poor clinical outcomes (1, 3). In prostate cancer, lineage plasticity is characterized by reduced AR expression/function, loss of epithelial identity, and activation of stem-like or alternative differentiation programs (3, 4). This plasticity represents a continuum of alternate differentiation programs (3, 4). Neuroendocrine prostate cancer (NEPC) is one of the most aggressive forms and has been increasing in incidence with the use of newer, more potent AR pathway inhibitors (1, 3, 4).

Loss of the tumor suppressors *TP53* and *RB1* is associated with lineage plasticity (5–7). Previous work demonstrates that loss of these factors promotes epithelial-mesenchymal transition (EMT) and neuronal differentiation while suppressing epithelial differentiation — 2 key aspects of lineage plasticity (5, 6). However, the molecular mechanisms by which *TP53*/*RB1* loss promotes lineage plasticity remain poorly characterized, and there are limited treatment options once lineage plasticity develops in prostate tumors.

Previous work by us and others demonstrated that *TP53*/*RB1* loss may induce neuronal differentiation through activation of reprogramming transcription factors (5, 6, 8). We also showed BET bromodomain inhibition (BETi) can block the lineage plasticity program activated in NEPC tumors (8). Notably, a subset of patients with NEPC experienced prolonged tumor control in our prior BETi clinical trial (8, 9). Unfortunately, disease progression was universal, highlighting the need to address additional mechanisms contributing to lineage plasticity and to develop rational combination therapies.

*TP53*/*RB1* loss also suppresses epithelial differentiation (5, 6) — in part by DNA methylation changes (10, 11). The maintenance DNA methyltransferase DNMT1 is upregulated with *TP53*/*RB1* loss (10). Genome-wide DNA methylation studies in patient tumors revealed global DNA methylation changes in NEPC compared with adenocarcinoma, including at the *AR* and its target genes (12, 13). Preclinical studies demonstrate growth suppression with DNMT inhibition (DNMTi) (10, 11, 14), suggesting that targeting DNA methylation may be a promising approach for these tumors but also highlighting the need for combination approaches.

In this study, we examined the effects of *TP53*/*RB1* loss on lineage plasticity. We showed that *TP53*/*RB1* loss activated EMT and stemness gene sets that were BET bromodomain protein-responsive and induced genome-wide DNA methylation changes that suppress genes that regulate or reflect epithelial differentiation. Hence, we hypothesized that simultaneously blocking BET bromodomain proteins and DNMTs would lead to more durable tumor control. Combined treatment with BETi and DNMTi in *TP53*/*RB1*-deficient mouse and patient-derived NEPC tumor models, both in vitro and in vivo*,* resulted in superior growth suppression compared with either agent alone and was well tolerated. Combination therapy modulated lineage plasticity and cancer-associated gene sets with an overall additive effect. Altogether, our findings support combining BETi and DNMTi as a promising therapeutic strategy for prostate cancers exhibiting lineage plasticity — largely due to the unique effects of each agent.

## Results

### TP53/RB1 loss induces changes in global chromatin hyper-accessibility and activates stemness, EMT, metastasis, and neuronal gene programs.

**Figure 1:**
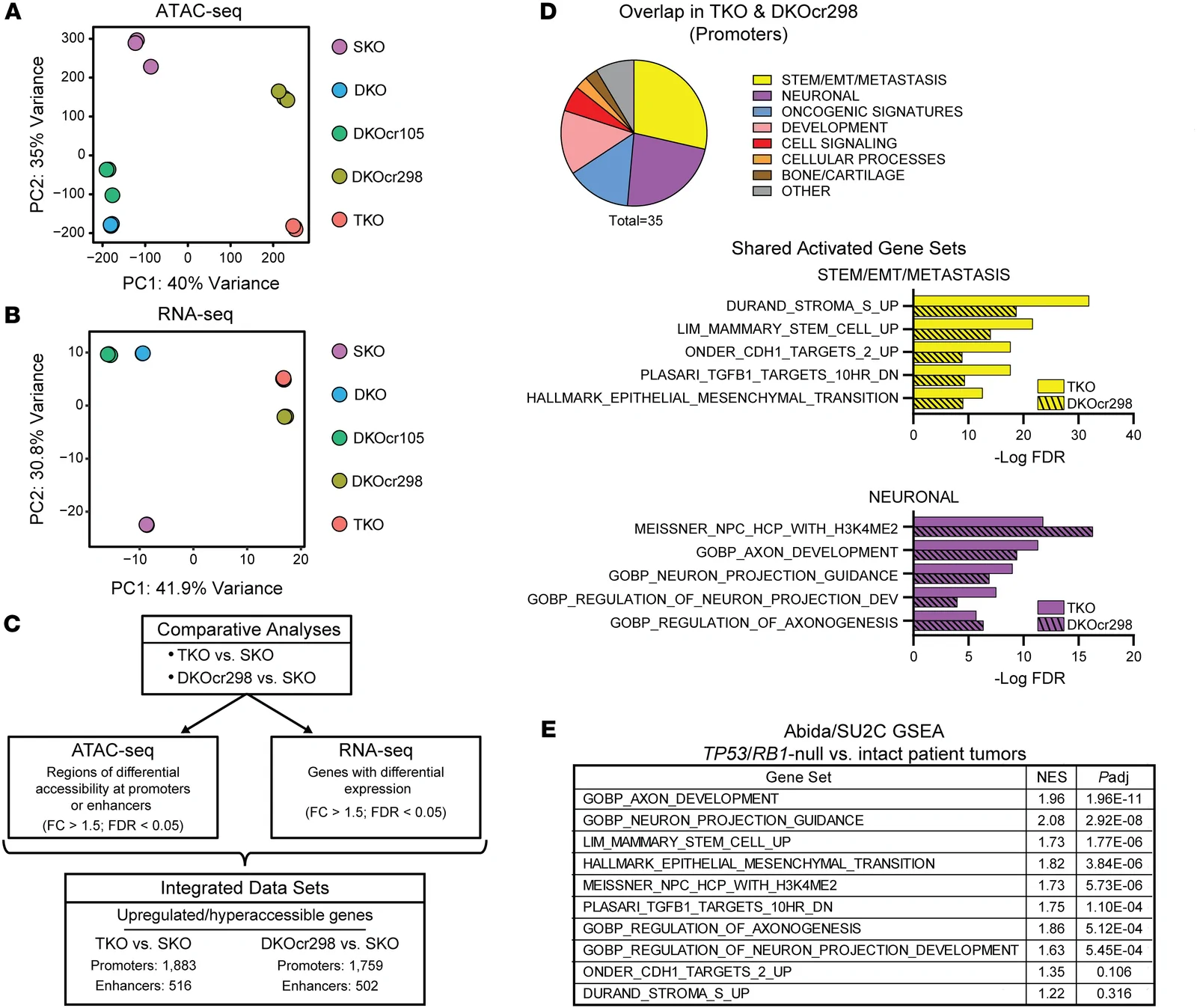
*Trp53*/*Rb1* loss leads to global chromatin hyper-accessibility changes and activation of stemness, EMT, metastasis, and neuronal genes. (**A** and **B**) PCA plots of ATAC-Seq data (**A**) and RNA-Seq data (**B**) from SKO, DKO, DKOcr105, DKOcr298, and TKO mouse cell lines (5); (*n* = 3 for all cell lines). (**C**) Schematic depicting analysis integrating differential hyper-accessible chromatin at promoters and enhancers (ATAC-Seq) corresponding with gene activation (RNA-Seq) from *Trp53*/*Rb1*-deficient (TKO; DKOcr298) versus *Trp53*/*Rb1*-intact (SKO) cell lines to determine genes activated with loss of *Trp53* and *Rb1*. (**D**) Top panel: top shared activated gene sets (FDR < 0.1) from MSigDB Hallmark, Chemical and Genetic Perturbations (CGP), and Gene Ontology Biological Process (GOBP) gene set collections between TKO and DKOcr298 cell lines that are enriched in activated genes with hyper-accessible promoter regions. Middle and bottom panels: representative top shared activated gene sets related to stemness, EMT, and metastasis (middle) and neuronal regulation (bottom) in TKO and DKOcr298 ranked by TKO FDR. Statistical significance was determined using hypergeometric test followed by Benjamini-Hochberg correction. (**E**) GSEA of *TP53*/*RB1*-null versus intact patient tumors (15) showing activation of EMT, stemness, metastasis, and neuronal gene sets depicted in **D**.

Prior work demonstrates that loss of *Trp53*, *Rb1*, and *Pten* in a genetically engineered mouse model (GEMM) drives lineage plasticity (5), mirroring observations in patient tumors (5–7). To understand the molecular events underlying this process, we performed assay for transposase-accessible chromatin sequencing (ATAC-Seq) and RNA-Seq on GEMM-derived cell lines with the following genotypes: *Pten* deletion (SKO); *Pten* and *Rb1* deletion (DKO); castration-emergent DKO (DKOcr105); castration-emergent DKO with a *Trp53* loss-of-function mutation (DKOcr298); and *Pten*, *Rb1*, and *Trp53* deletion (TKO) (5). Principal component analysis (PCA) revealed distinct shifts in chromatin accessibility (Figure 1A) and gene expression (Figure 1B) across *Rb1-* or *Trp53*/*Rb1*-deficient cell lines compared with SKO. Notably, *Trp53*/*Rb1*-loss tumors clustered together (i.e., TKO and DKOcr298).

We performed an integrated analysis of upregulated genes exhibiting promoter or enhancer hyper-accessibility in TKO and DKOcr298 compared with SKO (Figure 1C and Supplemental Table 1; supplemental material available online with this article; https://doi.org/10.1172/jci.insight.207543DS1). Gene set overrepresentation analysis (ORA) of upregulated genes with hyper-accessible promoters indicated robust activation of EMT, stemness, metastasis, neuronal, developmental, and oncogenic gene signatures (Supplemental Figure 1, A and B, and Supplemental Table 2). Most activated gene sets were conserved across TKO versus SKO and DKOcr298 versus SKO comparisons and primarily involved stemness, EMT, metastasis, and neuronal processes (Figure 1D and Supplemental Table 2). Activated enhancers corresponded to similar gene sets and overlapped substantially between TKO and DKOcr298 (Supplemental Figure 1, C–E, and Supplemental Table 2).

For clinical relevance, we analyzed RNA-Seq and DNA-Seq data from human castration-resistant prostate tumors with biallelic *TP53*/*RB1* loss versus intact tumors (15). Pathways activated in the *Trp53*/*Rb1-*loss cell line models were also activated in patient tumors (Figure 1E and Supplemental Table 3). These data suggest these genetically engineered models faithfully recapitulate the molecular pathways enriched in human tumors with a similar genotype (5). Overall, these findings indicate that *TP53*/*RB1* loss promotes global chromatin and transcriptional reprogramming associated with lineage plasticity.

### BET inhibition blocks induction of stemness, EMT, and metastasis pathways and impairs growth in TP53/RB1-deficient tumors.

**Figure 2:**
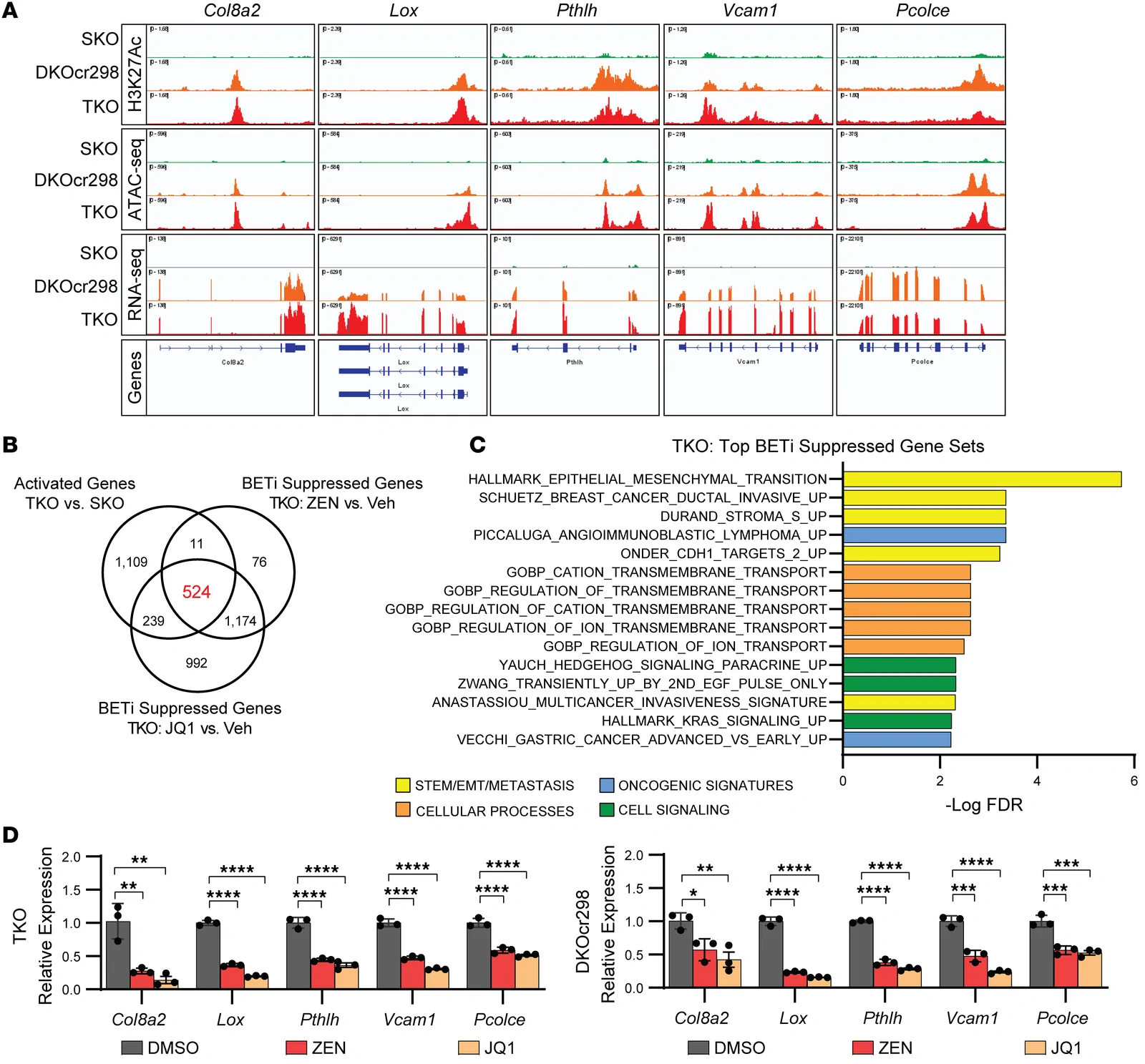
BETi blocks induction of stemness, EMT, and metastasis pathways in *Trp53*/*Rb1*-deficient tumors. (**A**) IGV (57) genome browser snapshots at BETi-sensitive genes from the Hallmark EMT gene set of H3K27Ac ChIP-Seq enrichment in SKO, DKOcr298, and TKO cell lines; chromatin accessibility (ATAC-Seq) in SKO, DKOcr298, and TKO cell lines; and gene expression (RNA-Seq) of SKO, DKOcr298, and TKO. (**B**) Venn diagram of activated genes with hyper-accessible promoter regions in TKO versus SKO but suppressed (fold change < 1.5; adj *P* < 0.05) with 24-hour treatment with 1 μM of the BET bromodomain inhibitor ZEN-3694 (ZEN) or 24-hour treatment with 500 nM of the BET bromodomain inhibitor JQ1 in TKO (*n* = 3 for each treatment condition). (**C**) Top gene sets (FDR < 0.1) from MSigDB Hallmark, Chemical and Genetic Perturbations (CGP), and Gene Ontology Biological Process (GOBP) gene set collections enriched in the intersection of 524 genes are shown. Statistical significance was determined using hypergeometric test followed by Benjamini-Hochberg correction. (**D**) RNA-qPCR of BETi-sensitive genes from the Hallmark EMT gene set in TKO and DKOcr298 treated with DMSO, 1 μM ZEN-3694 (ZEN), or 500 nM JQ1 for 24 hours. Data presented are the mean ± SD (*n* = 3 for all treated samples). Statistical significance was determined using 1-way ANOVA with Dunnett’s multiple-comparison test. *P < 0.05; **P < 0.01; ***P < 0.001; ****P < 0.0001.

BET bromodomain chromatin reader proteins bind to regions of high histone acetylation (e.g., H3K27Ac) throughout the genome to facilitate gene transcription and activation of a variety of differentiation programs (8, 16, 17). To determine whether *Trp53*/*Rb1* loss increases histone acetylation of genes associated with lineage plasticity, we performed H3K27Ac ChIP-Seq in TKO, DKOcr298, and SKO lines. Acetylation levels at genes from the Hallmark EMT gene set (18), which was activated in both TKO and DKOcr298 cell lines (Figure 1D), were increased and coincided with hyper-accessible chromatin at these regions (Figure 2A).

Previous studies, including our own, have demonstrated that BETi suppresses the emergence of pathways related to EMT and neuronal differentiation in treatment-induced NEPC models after AR pathway inhibitor therapy with enzalutamide (8, 19). We hypothesized BETi may also block the expression of genes activated by *TP53*/*RB1* loss. To test this, we treated TKO or DKOcr298 with the BETi JQ1 or ZEN-3694 (ZEN) — which is currently being tested in a phase II clinical trial in prostate cancer (ClinicalTrials.gov NCT04986423) — and performed RNA-Seq. We identified genes upregulated by *Trp53*/*Rb1* loss and repressed by both JQ1 and ZEN (Figure 2B and Supplemental Figure 2A) and then performed gene set ORA. The top gene sets were associated with stemness, EMT, and metastasis (Figure 2C and Supplemental Figure 2B). The full results are shown in Supplemental Table 4. We confirmed BETi suppressed expression of the EMT-associated genes highlighted in Figure 2A by RNA-qPCR, validating our RNA-Seq results (Figure 2D). These results demonstrate BETi suppresses gene networks activated through *Trp53*/*Rb1* loss that are linked to lineage plasticity or cancer aggressiveness.

We next assessed the antitumor activity of ZEN in TKO and DKOcr298 cells. In both cell lines, ZEN reduced cell viability in a dose-dependent manner (Supplemental Figure 2C). However, our prior ZEN clinical trial in prostate cancer showed limited objective tumor responses with BETi treatment, and all patients eventually progressed (9). This highlights the need to explore cotargeting strategies.

### TP53/RB1 loss induces global chromatin hypo-accessibility and DNA methylation changes that silence epithelial gene expression.

During our examination of chromatin accessibility changes induced by *TP53*/*RB1* loss, we observed numerous regions with reduced chromatin accessibility. In addition to activating neuronal and EMT programs, *TP53*/*RB1* loss is linked to suppression of epithelial differentiation (5–7). *RB1* loss alone has been linked to upregulation of DNMTs (14). NEPC tumors, which commonly harbor *TP53*/*RB1* loss-of-function alterations (7), also exhibit elevated DNMT expression (14) and aberrant DNA methylation relative to prostate adenocarcinomas (12, 13). Nevertheless, the specific effects of *TP53*/*RB1* loss on DNA methylation in prostate epithelial cells remained unclear. We hypothesized these *TP53*/*RB1* loss–induced hypo-accessible regions of chromatin might be associated with changes in DNA methylation and gene silencing.

**Figure 3:**
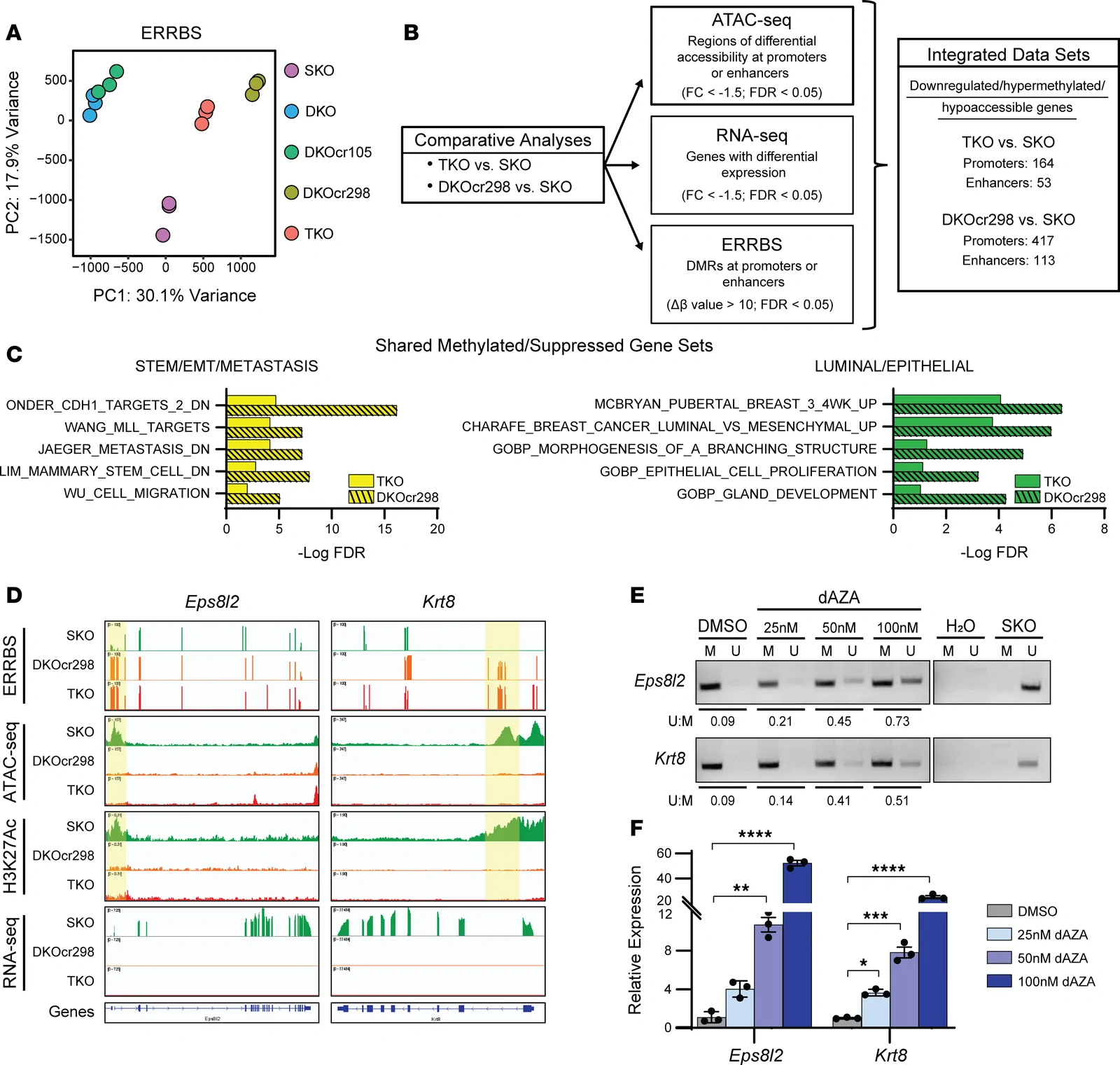
*Trp53*/*Rb1* loss leads to widespread chromatin hypo-accessibility and DNA methylation changes that suppress epithelial gene expression. (**A**) PCA plot of ERRBS data from SKO, DKO, DKOcr105, DKOcr298, and TKO mouse cell lines (5) (*n* = 3 for all cell lines). (**B**) Schematic depicting analysis integrating differential hypo-accessible chromatin at promoters and enhancers (ATAC-Seq) corresponding with increased DNA methylation (ERRBS) and gene suppression (RNA-Seq) from *Trp53*/*Rb1*-deficient (TKO; DKOcr298) versus *Trp53*/*Rb1*-intact (SKO) cell lines to determine methylated and silenced genes induced by loss of *Trp53* and *Rb1*. (**C**) Representative top shared methylated and suppressed gene sets (FDR < 0.1) from MSigDB Hallmark, 16. Chemical and Genetic Perturbations (CGP), and and Gene Ontology Biological Process (GOBP) gene set collections related to stemness, EMT, metastasis, and luminal/epithelial regulation in TKO and DKOcr298 ranked by TKO FDR. Statistical significance was determined using hypergeometric test followed by Benjamini-Hochberg correction. (**D**) IGV (57) genome browser snapshots showing DNA methylation (ERRBS), chromatin accessibility (ATAC-Seq), H3K27Ac ChIP-Seq enrichment, and gene expression (RNA-Seq) at selected genes associated with epithelial differentiation in SKO, DKOcr298, and TKO cell lines. Gene promoter regions are highlighted. (**E**) Methylation-specific PCR (MSPCR) analysis of *Trp53*/*Rb1* loss–induced hypermethylated and silenced genes in TKO treated with increasing doses of dAZA. Cells were treated daily for 72 hours. Methylated (M) and unmethylated (U) specific amplicons are shown for each indicated sample. The densitometry ratio of unmethylated to methylated products (U to M) is shown below their respective amplicon pairs. SKO is included as an unmethylated control. Representative samples from biological triplicates are shown for each condition. (**F**) RNA-qPCR of *Trp53*/*Rb1* loss–induced hypermethylated and silenced genes in TKO with increasing doses of dAZA. RNAs used in this analysis were derived from the same samples as in **E**. Data presented are the mean ± SD (*n* = 3 for each treatment condition). Statistical significance was determined using 1-way ANOVA with Dunnett’s multiple-comparison test. *P < 0.05; **P < 0.01; ***P < 0.001; ****P < 0.0001.

To address this hypothesis, we performed enhanced reduced representation bisulfite sequencing (ERRBS) in SKO, DKO, DKOcr105, DKOcr298, and TKO cells. Similar to our ATAC-Seq and RNA-Seq analyses, ERRBS PCA showed TKO and DKOcr298 clustering together (Figure 3A). We next performed an integrated analysis of ERRBS, ATAC-Seq, and RNA-Seq data to identify genes characterized by promoter or enhancer hypermethylation, reduced chromatin accessibility, and decreased expression in TKO or DKOcr298 cells relative to SKO (Figure 3B and Supplemental Table 1). Next, we performed gene set ORA. Downregulated gene sets associated with hypermethylated and hypo-accessible promoter regions were enriched for genes that are suppressed during EMT, metastasis, or acquisition of a stemness phenotype in addition to genes linked to luminal/epithelial differentiation (Figure 3C, Supplemental Figure 3, A–C, and Supplemental Table 5). Downregulated gene sets enriched with hypermethylated and hypo-accessible enhancer regions were associated with suppression of stemness, EMT, metastasis, and regulation of oncogenic signatures and developmental pathways (Supplemental Figure 3, D–F, and Supplemental Table 5).

Two of the top hypermethylated and silenced genes in TKO and DKOcr298 were *Eps8l2* and *Krt8* — the latter of which is linked to epithelial differentiation (5, 6). Both genes harbored extensive DNA methylation at their promoter regions coinciding with chromatin hypo-accessibility, loss of histone acetylation, and gene silencing in TKO and DKOcr298 versus SKO cells (Figure 3D). To evaluate the reversibility of *Trp53/Rb1* loss–induced hypermethylation, we treated TKO and DKOcr298 with a low-dose range of the DNMTi 5-aza-2’-deoxycytidine (dAZA) (20). Methylation-specific PCR (MSPCR) and RNA-qPCR analyses of these representative genes showed dose-dependent DNA demethylation and gene reactivation (Figure 3, E and F, and Supplemental Figure 3, G and H). We selected 100 nM dAZA for subsequent experiments because this concentration induced the most robust DNA demethylation and gene reactivation (Figure 3, E and F, and Supplemental Figure 3, G and H). Overall, these results demonstrate *Trp53/Rb1* loss induces global reprogramming of DNA methylation, particularly affecting genes linked to lineage plasticity. DNMTi can reverse this epigenetic silencing, highlighting a potential therapeutic strategy for prostate cancer.

### Combination BET and DNMT inhibition leads to superior growth suppression in TP53/RB1-deficient cells versus single-agent treatment.

**Figure 4:**
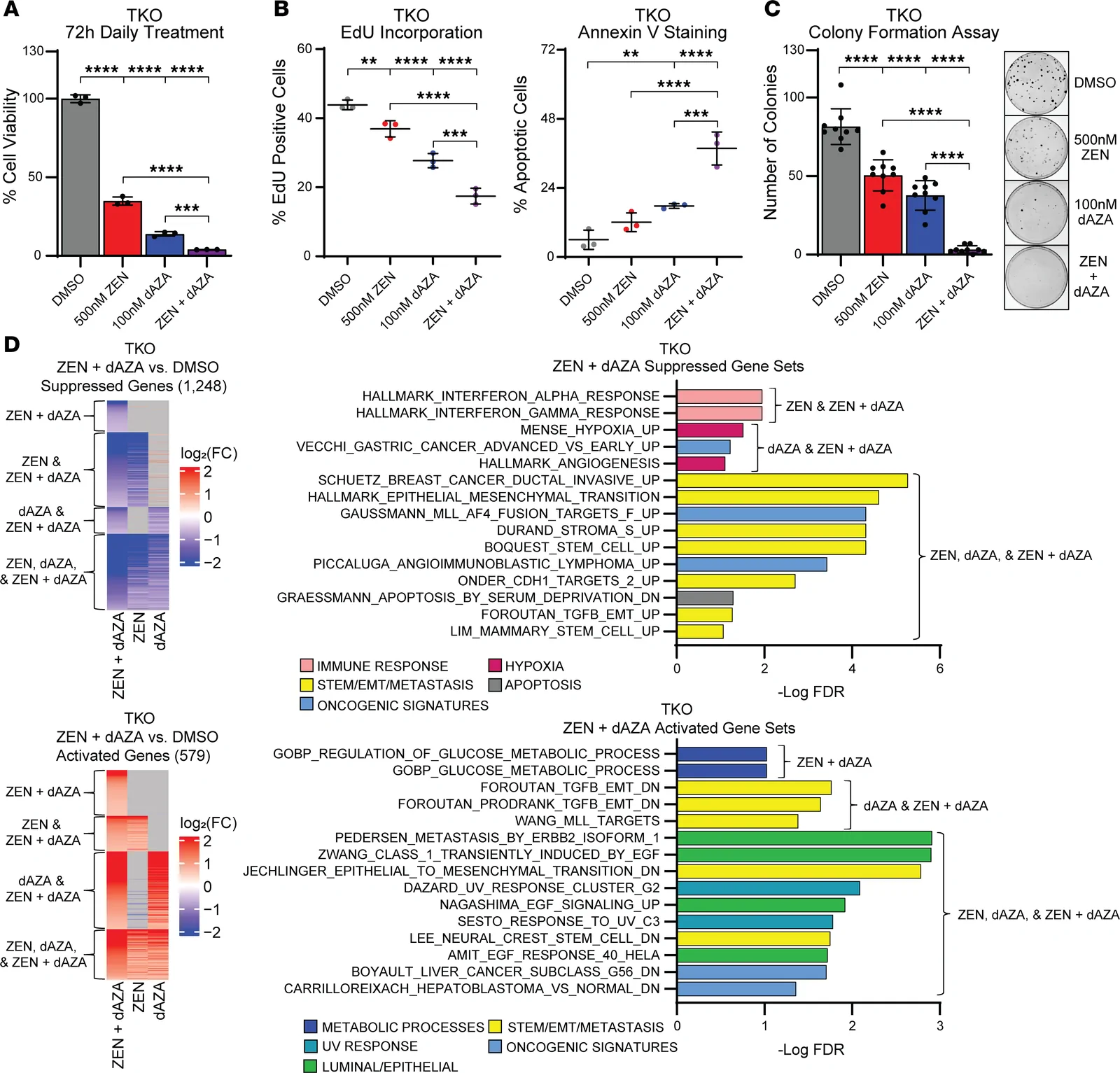
BETi and DNMTi combination treatment leads to superior growth suppression and reverses lineage plasticity gene expression in *Trp53*/*Rb1*-deficient cells in vitro. (**A**) Cell count viability of TKO cells after 72-hour daily treatment with indicated dose of ZEN, dAZA, or ZEN plus dAZA combination. (**B**) EdU incorporation and apoptosis assays using TKO cells after 48-hour daily treatment with indicated dose of ZEN, dAZA, or ZEN plus dAZA combination. (**C**) TKO colony formation assay (CFA) colony counts after long-term treatments. Cells were treated with indicated doses of ZEN, dAZA, or the combination for 72 hours daily before plating followed by sustained ZEN or ZEN-vehicle treatment without dAZA for 7 days. Representative scans are shown on the right. (**D**) Representative top gene sets (FDR < 0.1) composed of *Trp53*/*Rb1* loss–modulated genes reversed with ZEN plus dAZA combination treatment in TKO. Left: heatmaps depicting the relative differential gene expression in single-agent or combination-treated versus vehicle-treated control samples for *Trp53*/*Rb1* loss–modulated genes that are reversed with treatment. Right: gene set ORA on genes modulated by *Trp53*/*Rb1* loss–induced lineage plasticity that were reversed by combination treatment only (ZEN + dAZA), ZEN alone or combination treatment (ZEN and ZEN + dAZA), dAZA alone or combination treatment (dAZA and ZEN + dAZA), or either single agent alone or combination treatment (ZEN, dAZA, and ZEN + dAZA). Statistical significance was determined using hypergeometric test followed by Benjamini-Hochberg correction. Data presented in **A**–**C** are the mean ± SD (*n* = 3 for each treatment condition in **A** and **B**; *n* = 9 in **C**). Statistical significance in **A**–**C** was determined using 1-way ANOVA with Benjamini-Hochberg multiple testing correction. **P < 0.01; ***P < 0.001; ****P < 0.0001.

Though our results indicated BETi can block stemness, EMT, and metastasis-associated genes activated by *Trp53/Rb1* loss, BETi alone at the concentrations tested (up to 10 μM) did not completely eliminate tumor cells (Supplemental Figure 2C). *Trp53/Rb1* loss also induced widespread DNA methylation changes associated with loss of luminal or epithelial differentiation or tumor suppressors that may contribute to cell survival (Figure 3C) and that may be reversible through treatment with DNMTi. We hypothesized that combining BETi and DNMTi would target the complementary gene expression programs regulated by each target and yield a greater overall antitumor effect. To test this hypothesis, TKO and DKOcr298 cells were treated with 500 nM ZEN, 100 nM dAZA, or the combination of both agents daily for 72 hours. Treatment with either single agent reduced cell viability, but the combination had a more pronounced effect in both cell lines (Figure 4A and Supplemental Figure 4A). EdU incorporation and annexin V/propidium iodide staining assays showed the greatest suppression of cell proliferation and greatest apoptosis induction with combination treatment in TKO cells (Figure 4B). Combination treatment in DKOcr298 also had the greatest impact on suppressing cell proliferation but induced apoptosis to a similar degree as single-agent dAZA (Supplemental Figure 4B). In contrast, combination treatment in the *Trp53*/*Rb1*-intact adenocarcinoma cell line SKO did not further reduce cell viability beyond the effect of single-agent dAZA (Supplemental Figure 5A). We confirmed drug target engagement in SKO through *Hexim1* RNA-qPCR, which is upregulated with BETi treatment (21), and MSPCR at *Lrrc4*, which is one of the top methylated and silenced genes in SKO versus TKO (Supplemental Figure 5, B and C). These results suggest *Trp53*/*Rb1*-loss cells may be particularly sensitive to ZEN plus dAZA combination therapy.

We next tested long-term ZEN plus dAZA combination treatment in TKO and DKOcr298 tumor cells, similar to how these drugs are administered clinically — ZEN is administered continuously (9); dAZA is administered daily for 3–5 days followed by a multiweek holiday (20). After an initial 72-hour treatment with vehicle, ZEN, dAZA, or the combination, cells were plated for colony formation assays with continued ZEN or vehicle treatment every 72 hours without further treatment with dAZA. In both TKO and DKOcr298 cell lines, up-front combination-treated cells formed the fewest colonies, suggesting this combination may impair replicative potential (Figure 4C and Supplemental Figure 6). Lastly, we used the Bliss independence model (22) and found the combination had an additive effect (Supplemental Figure 7).

Because combination therapy was superior in suppressing growth, we sought to identify gene expression changes that might explain these effects. Drug-induced changes in gene expression and DNA methylation were confirmed in TKO and DKOcr298 by RNA-qPCR and MSPCR (Supplemental Figure 8, A–D) as well as RNA-Seq and ERRBS (Supplemental Figure 8, E–H). PCA of RNA-Seq of 72-hour–treated cells showed distinct clustering by treatment group, indicating unique effects of both single-agent and combination treatments on gene expression (Supplemental Figure 8, E and F). ERRBS PCA in TKO and DKOcr298 showed vehicle and single-agent ZEN-treated cells largely overlapping, indicating ZEN did not affect global DNA methylation (Supplemental Figure 8, E and F). However, single-agent dAZA and combination-treated cells clustered together and separated from vehicle and ZEN groups (Supplemental Figure 8, E and F), suggesting a similar DNA methylation pattern. Global assessment of DNA methylation changes also indicated a very similar pattern of DNA demethylation with dAZA versus the ZEN plus dAZA combination (Supplemental Figure 8, G and H).

Next, we aimed to determine whether lineage plasticity–associated genes modulated by *Trp53/Rb1* loss were reversed by ZEN plus dAZA. In both TKO and DKOcr298, we compared genes significantly upregulated or downregulated versus SKO with genes modulated in the opposite direction by single-agent or combination treatment (Supplemental Figure 9A). Some lineage plasticity genes were uniquely reversed with combination treatment. However, most were also reversed by single-agent ZEN or dAZA treatment (Supplemental Figure 9A). This suggests the overall combination treatment effect is largely additive between ZEN and dAZA, consistent with our Bliss independence model analysis of cell viability (Supplemental Figure 7). Importantly, these include genes previously implicated in advanced prostate cancer, neuronal regulators, and stemness factors (Supplemental Table 6). To further explore the effect of combination treatment on genes associated with prostate cancer cell viability, we compared combination treatment upregulated and downregulated genes in TKO and DKOcr298 with genes found to be important for cell viability in the NEPC cell line model NCI-H660 (23) via the Cancer Dependency Map (DepMap) project (24) (https://depmap.org/portal). We found many genes essential in NCI-H660 were downregulated with combination treatment in TKO and DKOcr298 (Supplemental Table 7; dependency score < –0.5). Additionally, we also identified putative tumor suppressors (i.e., genes that improved NCI-H660 viability upon knockdown/KO) that were upregulated in both cell lines with combination treatment (Supplemental Table 7; dependency score > 0.5).

To identify lineage plasticity–associated gene sets uniquely reversed with combination treatment, we performed gene set ORA on genes affected by *Trp53*/*Rb1* loss (TKO or DKOcr298 versus SKO comparison) that changed in the opposite direction with combination treatment (Supplemental Figure 9A). In TKO, genes activated with *Trp53/Rb1* loss but suppressed with combination treatment were largely conserved with single-agent treatments and associated with immune response, hypoxia, stemness/EMT/metastasis, oncogenic signatures, and blocking apoptosis (Figure 4D and Supplemental Table 8). Similarly, genes downregulated by *Trp53/Rb1* loss in TKO but activated with combination treatment were largely conserved with single-agent treatments. These gene sets included those downregulated during stemness/EMT/metastasis or in oncogenesis. Luminal/epithelial gene sets containing genes downregulated by *Trp53/Rb1* loss were also enriched in the genes reactivated with treatment (Figure 4D and Supplemental Table 9). Glucose metabolism genes were also uniquely activated with combination treatment (Figure 4D and Supplemental Table 9).

Genes that were upregulated in DKOcr298 versus SKO but were suppressed with combination treatment were enriched in immune response, stemness/EMT/metastasis, and cell adhesion gene sets and were conserved with single-agent treatments (Supplemental Figure 9B and Supplemental Table 10). Genes suppressed in DKOcr298 versus SKO but activated with combination treatment were associated with genes downregulated during oncogenesis or stemness/EMT/metastasis, genes downregulated with loss of a luminal/epithelial phenotype, genes whose downregulation blocks apoptosis, and immune response (Supplemental Figure 9B and Supplemental Table 11). These combination treatment–activated genes were largely conserved with single-agent treatment (Supplemental Figure 9B). Notably, many of these treatment-reversed gene sets in TKO and DKOcr298 were previously identified as associated with lineage plasticity from our *Trp53/Rb1-*loss integrated analysis (Figure 1D, Figure 3C, Supplemental Table 2, and Supplemental Table 5). These results indicate the combination treatment response is largely additive with conserved effects of each single agent. Overall, these findings suggest that the ZEN plus dAZA combination counteracts many of the gene expression changes induced by *Trp53/Rb1* loss.

### Combination BET and DNMT inhibition leads to superior growth suppression in NEPC cells versus single-agent treatment.

**Figure 5:**
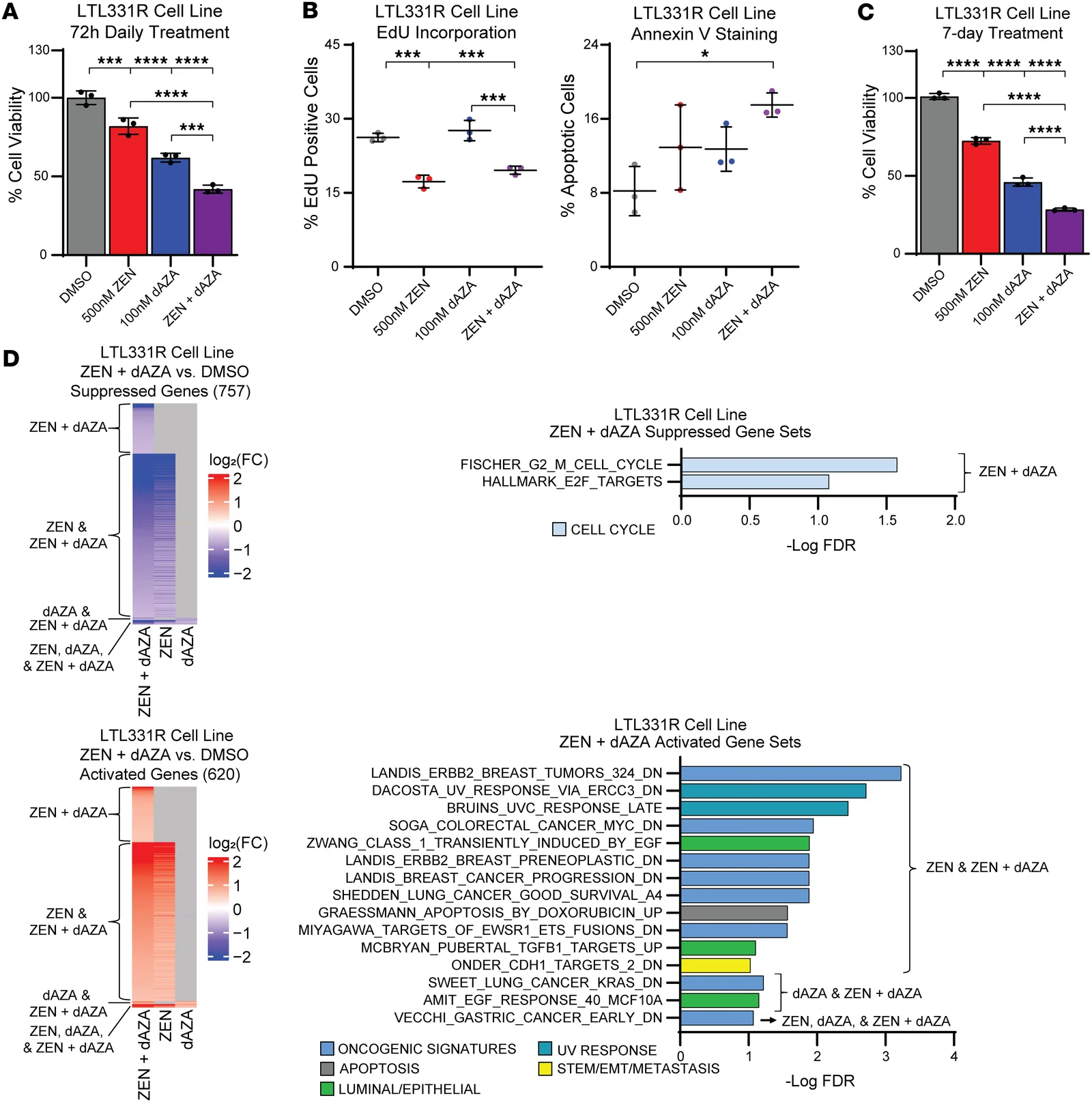
BETi and DNMTi combination treatment leads to superior growth suppression in NEPC in vitro. (**A**) Cell count viability of LTL331R (26) cells after 72-hour daily treatment of indicated dose of ZEN, dAZA, or ZEN plus dAZA combination. (**B**) EdU incorporation and apoptosis assays using LTL331R (26) cells after 72-hour daily treatment with indicated dose of ZEN, dAZA, or ZEN plus dAZA combination. (**C**) Cell count viability of LTL331R (26) cells after long-term treatments. Cells were treated with indicated doses of ZEN, dAZA, or the combination for 72 hours daily before plating followed by sustained ZEN or ZEN-vehicle treatment without dAZA for 7 days. (**D**) Representative top gene sets (FDR < 0.1) composed of castration-induced lineage plasticity–modulated genes that are reversed with ZEN plus dAZA combination treatment in LTL331R (26) cells. Left: heatmaps depicting the relative differential gene expression in single-agent or combination-treated versus vehicle-treated control samples for castration-induced lineage plasticity–modulated genes that are reversed with treatment. Right: gene set ORA on genes modulated by castration-induced lineage plasticity that were reversed by combination treatment only (ZEN + dAZA), ZEN alone or combination treatment (ZEN and ZEN + dAZA), dAZA alone or combination treatment (dAZA and ZEN + dAZA), or either single agent alone or combination treatment (ZEN, dAZA, and ZEN + dAZA). Statistical significance was determined using hypergeometric test followed by Benjamini-Hochberg correction. Data presented in **A**–**C** are the mean ± SD (*n* = 3 for each treatment condition in **A**–**C**). Statistical significance in **A**–**C** was determined using 1-way ANOVA with Benjamini-Hochberg multiple testing correction. *P < 0.05; **P < 0.01; ***P < 0.001; ****P < 0.0001.

In addition to *TP53/RB1* loss–induced reprogramming, castration or AR pathway inhibitors can induce NEPC transdifferentiation, and NEPC tumors are enriched for *TP53/RB1* loss (1, 7, 13, 25). Therefore, we next focused on treatment-induced NEPC models. We tested combination treatment in the LTL331R NEPC cell line, which is derived from an NEPC patient-derived xenograft (PDX) that emerges from an adenocarcinoma (LTL331) tumor after castration in mice (26) or the NEPC cell line NCI-H660 (23). Notably, both these cell lines have functional *TP53*/*RB1* loss (27). Single-agent treatment reduced cell viability, but combination treatment had a greater effect in both cell lines (Figure 5A and Supplemental Figure 10A). In LTL331R, ZEN and combination treatments significantly blocked cell proliferation, whereas dAZA alone had no effect (Figure 5B). Combination treatment also resulted in the greatest induction of apoptosis (Figure 5B). In NCI-H660 cells, ZEN and combination treatments similarly blocked cell proliferation, but apoptosis was not significantly induced (Supplemental Figure 10B). We also tested ZEN plus dAZA combination treatment in the *TP53*/*RB1*-intact adenocarcinoma cell lines LNCaP and V16D (6, 7, 28). Both single-agent treatments blocked adenocarcinoma cell viability, but combination treatment did not reduce viability to a greater extent than single-agent ZEN (Supplemental Figure 11A), in contrast to results observed in NEPC. Drug target engagement was confirmed by RNA-qPCR and MSPCR (Supplemental Figure 11, B and C).

We next assessed the impact of long-term ZEN plus dAZA combination treatment on cell viability in LTL331R and NCI-H660 cells. Cells were treated daily with vehicle, 500 nM ZEN, 100 nM dAZA, or the combination for 72 hours. After treatment, an equal number of viable cells from each treatment group was replated and cultured for an additional 7 days with continued ZEN or vehicle treatment in the absence of dAZA. In both cell lines, long-term treatment with either single agent reduced cell viability, but combination up-front treatment had the greatest effect (Figure 5C and Supplemental Figure 12). These results with combination therapy in NEPC models are consistent with results in *Trp53/Rb1*-deficient mouse tumor cells (Figure 4C and Supplemental Figure 6).

We investigated the impact of single-agent and combination treatment on gene expression in NEPC cells. Drug-mediated changes in gene expression and DNA methylation were verified by RNA-Seq and ERRBS (Supplemental Figure 13, A–D) and confirmed by RNA-qPCR and MSPCR at genes identified from RNA-Seq and ERRBS (Supplemental Figure 13, E and F). PCA of RNA-Seq showed samples segregated by treatment, demonstrating distinct effects on global gene expression with either single-agent or combination treatment (Supplemental Figure 13, A and B) and consistent with observations in TKO and DKOcr298 cells. ERRBS PCA showed global DNA methylation patterns were primarily influenced by dAZA treatment, with no effect from ZEN treatment alone (Supplemental Figure 13, A and B). Global measures of DNA methylation showed similar DNA methylation changes with combination or dAZA treatment (Supplemental Figure 13, C and D).

We next examined whether combination treatment could reverse the gene expression changes associated with castration-induced lineage plasticity. To identify castration-induced lineage plasticity genes, we compared differential expression between LTL331R and LTL331 PDXs (26). We then compared genes upregulated or downregulated in LTL331R versus LTL331 with genes that had the opposite effect with combination treatment in LTL331R or NCI-H660 cell lines (Supplemental Figure 14A). As observed in TKO and DKOcr298 cells, a small subset of these genes was uniquely reversed by combination treatment in both NEPC cell lines (Supplemental Figure 14A). However, most genes reversed by combination treatment were also reversed with single-agent ZEN alone (Supplemental Figure 14A). In contrast, dAZA single-agent treatment led to very few differentially expressed genes (DEGs), despite blocking cell viability to a greater extent than single-agent ZEN treatment (Figure 5A and Supplemental Figure 10A). Notably, genes reversed with combination treatment included a number of neuronal stemness regulatory factors, including suppression of *SOX2* (6, 29) and upregulation of *REST*, which is a well-characterized suppressor of neuronal differentiation (29) (Supplemental Table 12). We also compared combination treatment upregulated and downregulated genes in LTL331R and NCI-H660 cell lines with DepMap (24) NCI-H660 genes. Similar to our results in TKO and DKOcr298 cell lines, we observed combination treatment suppressed essential genes (dependency score < –0.5) and upregulated putative tumor suppressors (dependency score > 0.5) in LTL331R and NCI-H660 (Supplemental Table 7).

To define gene sets associated with castration-induced lineage plasticity that were reversed by treatment, we performed ORA on genes differentially expressed between LTL331R and LTL331 PDXs and modulated in the opposite direction by single-agent or combination therapy (Supplemental Figure 14A). In LTL331R cells, genes upregulated by castration and uniquely suppressed by combination treatment were enriched in cell cycle–associated gene sets (Figure 5D and Supplemental Table 13). Conversely, genes downregulated by castration but reactivated by treatment — particularly those restored by ZEN alone or conserved with the combination — were associated with DNA damage response, luminal/epithelial differentiation, and suppression of oncogenic signatures, including stemness, EMT, and metastasis downregulated genes (Figure 5D and Supplemental Table 14). In NCI-H660 cells, both ZEN and combination treatment blocked activation of genes upregulated in LTL331R versus LTL331 tumors related to cell adhesion and neuronal regulation (Supplemental Figure 14B and Supplemental Table 15). These treatments also specifically reactivated pathways associated with DNA damage response and suppression of oncogenesis. Metabolic process genes were reactivated by all 3 treatments — dAZA, ZEN, and combination (Supplemental Figure 14B; Supplemental Table 16). Collectively, these findings demonstrate that combination therapy effectively reverses important castration-induced lineage plasticity gene expression changes, and those effects are largely driven by the ZEN effects.

### Combination BET and DNMT inhibition leads to superior growth suppression in lineage plasticity models in vivo.

**Figure 6:**
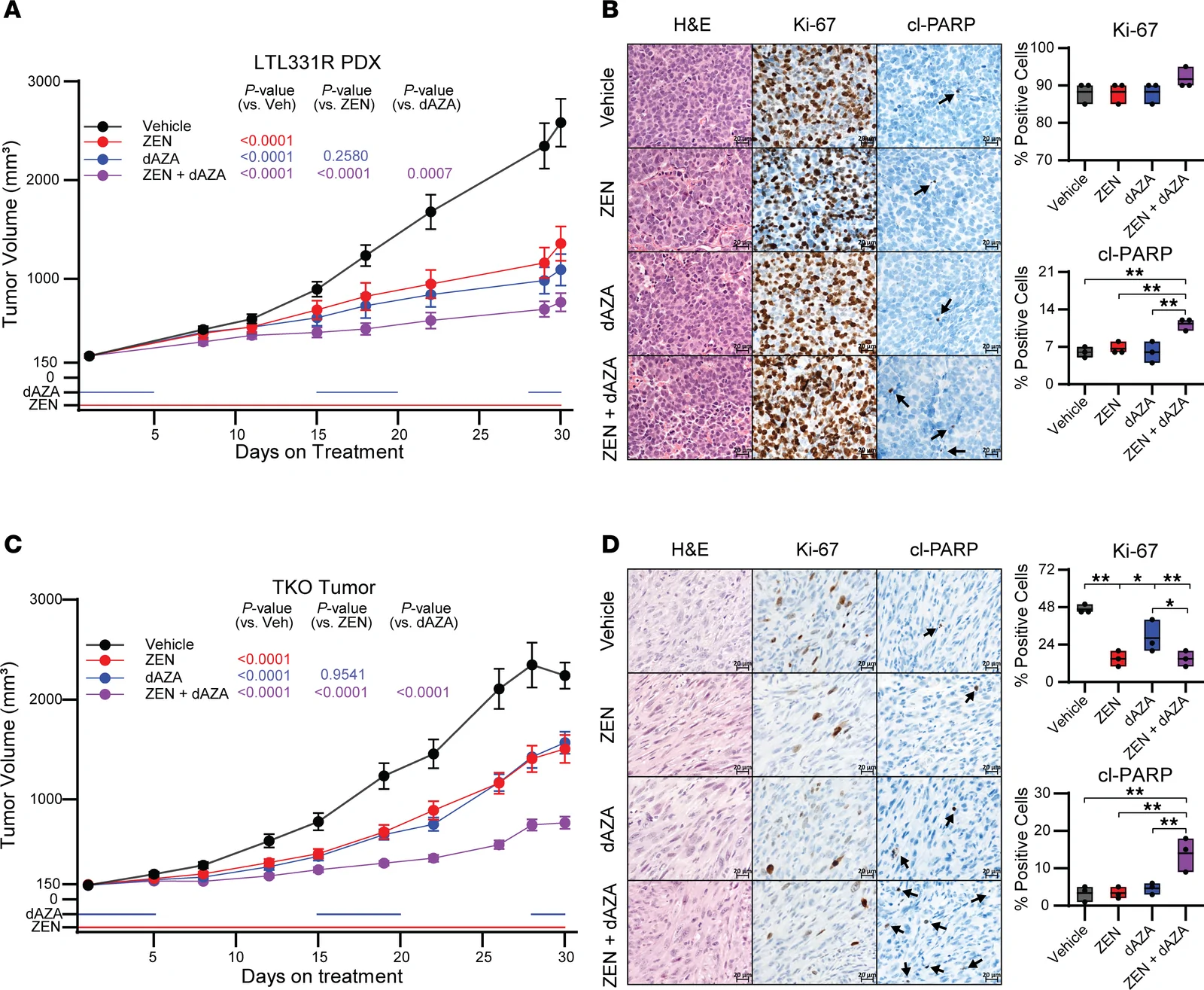
BETi and DNMTi combination treatment leads to superior growth suppression in lineage plasticity models in vivo. (**A** and **C**) Growth of LTL331R (26) PDXs treated with vehicle (*n* = 12), ZEN (50 mg/kg; *n* = 12), dAZA (0.8 mg/kg; *n* = 12), or ZEN plus dAZA (*n* = 15) (**A**) and TKO tumors treated with vehicle (*n* = 11), ZEN (50 mg/kg; *n* = 11), dAZA (0.4 mg/kg; *n* = 10), or ZEN plus dAZA (*n* = 11) (**C**) are shown. The treatment schedule for the duration of each study is indicated by colored bars beneath the tumor growth data. Data shown are the mean ± SEM. Statistical significance was determined using 2-way ANOVA followed by Tukey’s multiple-comparison test. (**B** and **D**) LTL331R (26) PDXs (**B**) and TKO tumors (**D**) harvested 5 days after treatment start were sectioned and stained with H&E or antibodies against the indicated proteins. Arrowheads indicate positively stained nuclei. Scale bar: 20 μm. Quantification of immunostaining is shown on the right and presented as floating bars representing the minimum and maximum values with the mean indicated (*n* = 3 for each treatment condition). Statistical significance was determined using 1-way ANOVA with Benjamini-Hochberg multiple testing correction. *P < 0.05; **P < 0.01.

To determine the antitumor efficacy of the ZEN plus dAZA combination treatment in vivo in NEPC, we treated mice implanted with LTL331R xenografts (26) with ZEN, dAZA, or the combination using established dosing schedules (9, 20). We also treated TKO cells implanted in mice with the same schedule but using a lower dose of dAZA due to the greater in vitro sensitivity of this cell line versus LTL331R cells (Figure 4A and Figure 5A). Consistent with our in vitro results, both single-agent treatments significantly inhibited tumor growth. However, combination treatment produced a greater growth-suppressive effect (Figure 6, A and C). To assess toxicity, we monitored mouse body weights throughout each study and observed no significant reduction with treatment (Supplemental Figure 15, A and C). Hematological toxicity is also a known side effect of each drug (9, 30). However, no reduction in blood cell counts was observed (Supplemental Figure 15, B and D), confirming a favorable safety profile of combination treatment. To test for the impact of combination treatment on cell proliferation and apoptosis, we measured levels of Ki-67 and cleaved PARP, respectively, by IHC. In LTL331R PDXs, there were no significant differences in the proliferation marker Ki-67 among treatment groups. However, combination-treated LTL331R tumors showed an increase in cleaved PARP, demonstrating enhanced apoptosis compared with the single-agent and control groups (Figure 6B). In TKO tumors, IHC analysis showed a significant reduction in Ki-67 staining in single-agent ZEN, single-agent dAZA, and combination-treated tumors (Figure 6D). Ki-67 staining in tumors treated with ZEN alone or the ZEN plus dAZA combination was reduced to a similar level and exhibited greater reduction than single-agent dAZA (Figure 6D). Combination-treated tumors were also enriched for cleaved PARP compared with vehicle- or single agent–treated tumors (Figure 6D). Overall, these findings support ZEN plus dAZA as a well-tolerated and promising treatment approach for NEPC or *TP53*/*RB1*-deficient tumors.

Consistent with our in vitro experiments, PCA of RNA-Seq from LTL331R PDXs revealed clear clustering of tumors by treatment group and was largely driven by ZEN. PCA of ERRBS separated samples primarily by dAZA exposure (Supplemental Figure 16A). ERRBS also showed global levels of DNA methylation were reduced with dAZA or combination treatment (Supplemental Figure 16B) We verified gene expression and DNA methylation changes of genes identified from RNA-Seq and ERRBS by RNA-qPCR and MSPCR (Supplemental Figure 16, C and D). We observed that dAZA induced fetal globin gene reactivation in mouse bone marrow, which has been shown to be silenced through DNA methylation (31) (Supplemental Figure 16E). We also confirmed target engagement in TKO experiments by measuring reactivation of fetal globin genes in bone marrow and expression of the BETi-responsive gene *Hexim1* (21) in TKO tumors (Supplemental Figure 17, A and B).

To determine whether the combination could reverse castration-induced lineage plasticity gene expression in vivo, we identified genes differentially expressed between LTL331R and LTL331 PDXs and analyzed their response to treatment in LTL331R PDXs (Supplemental Figure 18A). Consistent with our in vitro findings, a subset of lineage plasticity genes was uniquely reversed by combination therapy; however, most of the genes changing with castration and reversed with combination treatment were reversed by ZEN alone, with dAZA having only a minor effect on gene expression (Supplemental Figure 18A). In particular, the combination reversed expression changes of important reprograming factors, including *SOX2* (6, 29), *REST* (29), *PROX1* (32), and *ASCL1* (14) that were differentially expressed in LTL331R versus LTL331 (Supplemental Table 12). These results demonstrate that, in vivo, ZEN and dAZA combination therapy effectively reverses castration-induced lineage plasticity gene expression, predominantly via ZEN treatment effects. We compared combination treatment upregulated and downregulated genes in LTL331R PDXs with essential genes or putative tumor suppressors in NCI-H660 identified via DepMap (24). Similar to in vitro models tested, combination treatment downregulated essential genes (dependency score < –0.5) and upregulated putative tumor suppressors (dependency score > 0.5) (Supplemental Table 7).

We performed gene set ORA on castration-induced lineage plasticity genes whose expression change was reversed by each treatment (Supplemental Figure 18B). Notably, gene sets associated with the cell cycle were uniquely suppressed by the combination treatment (Supplemental Figure 18B and Supplemental Table 17). Genes that were downregulated in LTL331R versus LTL331 but reactivated by combination treatment and single-agent treatment were enriched in gene sets downregulated in oncogenesis and gene sets linked to luminal/epithelial regulation, among others (Supplemental Figure 18B and Supplemental Table 18). These results suggest that combined dAZA and ZEN modulates the expression of key pathways enriched in tumors that have undergone NEPC transdifferentiation.

Finally, we sought to determine the effect of combination treatment on differentiation state in lineage plasticity tumor cells. Histological examination of H&E-stained tumors did not show changes in cell morphology with either single-agent treatment or combination treatment, indicating BETi and DNMTi combination treatment did not influence the phenotype of treated cells at the time point examined (Figure 6, B and D). To further explore this, we performed a differentiation signature enrichment analysis using VIPER (33). Combination treatment did not reverse gene sets associated with luminal (6, 34) or neuroendocrine (13) differentiation in most models (Supplemental Table 19). The one exception was the LTL331R PDX, in which combination treatment did significantly reduce enrichment of a neuroendocrine differentiation gene signature (Supplemental Table 19). This is consistent with the observed downregulation of *ASCL1*, which is a master regulator of neuroendocrine differentiation (35), and upregulation of *REST*, which functions to repress neuroendocrine differentiation (29) (Supplemental Table 12). Overall, these results suggest combination treatment modulates important survival pathways induced by *TP53/RB1* loss or castration in lineage plasticity tumors but does not reverse the lineage plasticity phenotype.

## Discussion

Loss of *TP53*/*RB1* is associated with lineage plasticity, also known as transdifferentiation, in prostate cancer (5–7). Experimental evidence indicates that loss of these tumor suppressors may activate a stemness/neuronal gene program while concurrently repressing epithelial differentiation (5, 6). Our results further support *TP53*/*RB1* as critical suppressors of lineage plasticity and provide molecular insights into epigenetic changes underlying these effects. Our multiomic approach identified global shifts in chromatin accessibility and DNA methylation that impact key contributors to lineage plasticity in prostate cancer (i.e., loss of epithelial differentiation with activation of an EMT, stemness, and neuronal program). Importantly, we confirmed that many of the gene sets modulated by *TP53*/*RB1* loss in our models were highly enriched in patient tumors with *TP53*/*RB1* loss, demonstrating the clinical relevance of the pathways identified. Moreover, we identified a rational co-targeting approach for these tumors — combined BETi and DNMTi — which produced superior antitumor activity compared with either agent alone.

Alternative differentiation programs are linked to the activity of BET bromodomain proteins (8, 16, 17, 19). We and others have previously shown BET bromodomain proteins drive lineage plasticity survival programs associated with neuronal, stemness, and EMT gene activation in treatment-induced NEPC, which can be effectively blocked through BETi (8, 19). Consistent with these prior findings, our current results demonstrate the lineage plasticity program induced by *TP53*/*RB1* loss is highly enriched in genes associated with stemness and EMT and can also be blocked by BETi, further supporting BET proteins as key drivers of lineage plasticity. We also observed suppression of castration-induced lineage plasticity genes activated in the LTL331R (26) cell line or PDX and NCI-H660 cell line with BETi. ZEN-suppressed lineage plasticity genes in the LTL331R (26) models were not significantly enriched in any particular gene set category, suggesting BETi targets lineage plasticity genes across a wide variety of pathways rather than a few discrete processes in this model. In contrast, castration-induced lineage plasticity genes associated with neuronal regulation and cell adhesion were blocked with BETi in NCI-H660. Indeed, cell adhesion is a key process governing growth and maintenance of epithelial differentiation and is commonly dysregulated during cancer progression (36). Notably, the principal BETi we used in our studies — ZEN-3694 — showed antitumor activity, including in NEPC tumors, in a prior phase Ib clinical trial (8, 9). However, patient tumor progression was universal (8), demonstrating the need to develop rational BETi combinations.

Loss of epithelial differentiation is a hallmark of lineage plasticity in prostate cancer (3) and is promoted by *TP53*/*RB1* loss (5, 6). DNMTs are upregulated with lineage plasticity (10, 37), and there are widespread DNA methylation changes in NEPCs separating them from adenocarcinomas (13). Our multiomic profiling revealed extensive DNA methylation changes associated with suppressing epithelial differentiation and promoting EMT/stemness after *TP53*/*RB1* loss. DNMTi treatment promotes epithelial differentiation, suppresses tumorigenesis, and prolongs survival in *TP53*/*RB1*-inactivated TRAMP tumors (10, 11), consistent with our results in TKO and DKOcr298 cell lines with single-agent dAZA. DNMTi also suppressed the growth of NEPC models in vivo that coincided with downregulation of NEPC-associated genes (14). Similarly, we observed single-agent dAZA treatment in LTL331R PDXs blocked tumor growth and suppressed lineage plasticity–associated genes (e.g., *SOX2, ASCL1,* *PROX1*) in vivo. Our findings suggest abrogation of key lineage plasticity gene sets contributes to single-agent dAZA antitumor activity. However, single-agent DNMTi treatment has shown limited efficacy in prior clinical trials in CRPC (38, 39), demonstrating the need to develop combinatorial DNMTi strategies.

We hypothesized that combining BETi and DNMTi would exert greater antitumor effects than either single agent alone because of the unique pathways modulated by each drug that were important for lineage plasticity tumor survival. Our results confirm this hypothesis, demonstrating that combination treatment more effectively suppressed tumor growth in both *TP53*/*RB1*-deficient stem-like and NEPC models. This enhanced antitumor effect was mediated, at least in part, by inhibition of the cell cycle and induction of apoptosis. Furthermore, we demonstrated in vitro using colony formation assays and in vivo using implanted tumor models that long-term combination treatment versus single-agent treatment results in greater tumor control despite breaks in DNMTi drug administration. Notably, combination treatment was well-tolerated with little observed toxicity in vivo, consistent with results from a recent phase I clinical trial in patients with myelodysplastic syndromes (40). These results motivate and provide a framework for combined BETi and DNMTi dosing schedules for future prostate cancer clinical trials. Interestingly, in our experiments, combination treatment did not suppress tumor growth to a greater extent than single agents in the adenocarcinoma models we examined. This suggests cells that have undergone lineage plasticity may be particularly sensitive to BETi and DNMTi combination treatment.

Transcriptional profiling revealed that combination treatment affected a broad spectrum of gene sets. Across all models tested, combination treatment abrogated the expression of numerous genes induced by *TP53*/*RB1* loss or castration-induced NEPC transdifferentiation and modulated key cancer pathways, including those related to hypoxia, DNA damage response, EMT, cell cycle, and apoptosis. Notably, gene expression changes observed with single agents were largely preserved with combination therapy, and we did not find evidence of a synergistic interaction using the Bliss independence model (22), which indicated an additive effect of treatment. The modulation of gene expression induced by combination treatment did not appear to result from further loss of DNA methylation versus dAZA alone but likely reflects the regulation of distinct genes by BETi and DNMTi. Our differentiation signature analysis and histological examination of treated cell lines and PDXs suggest the BETi and DNMTi combination did not revert the cell phenotype to a more luminal, or epithelial, differentiation state. Rather, combination treatment may block pathways important for cell survival in cells exhibiting a lineage plasticity or transdifferentiated phenotype. Interestingly, dAZA single-agent treatment had modest effects on gene expression despite significantly impairing NEPC tumor viability. This suggests dAZA treatment targets a subset of genes critical for NEPC cell survival. Importantly, although dAZA requires incorporation into genomic DNA during the S-phase (41), its antitumor efficacy across all the models we examined was not abrogated with concurrent ZEN treatment.

There are several limitations of our findings. Given the marked heterogeneity of advanced prostate cancer (4), it is unsurprising that combination treatment influenced different pathways in the models we examined, likely reflecting diverse epigenetic states of the models used. Nevertheless, our data suggest a dependency of both BET bromodomain proteins and DNMTs for survival of tumors exhibiting lineage plasticity and demonstrate the worthiness of cotargeting these factors. Notably, combination treatment was not curative. It is possible higher doses of BETi and DNMTi may be required for more complete antitumor activity, which may be possible given the tolerability we observed. Further studies are also necessary to understand adaptive resistance mechanisms whose suppression may further augment the efficacy of combination treatment. Altogether, our findings support the BETi and DNMTi combination as a safe and promising therapeutic approach to combat aggressive subsets of prostate cancer with *TP53*/*RB1* loss, including NEPC.

## Methods

### Sex as a biological variable.

Our study exclusively examined male mice because the disease modeled is only relevant in males.

### Cell lines and PDX.

SKO, DKO, DKOcr105, DKOcr298, and TKO cell lines were described previously (5). SKO, DKO, DKOcr105, and TKO cells were cultured in DMEM/high glucose (Gibco, 11995-065) with L-glutamine (Gibco, 35050-061), sodium pyruvate (Gibco, 11360-070), and 10% FBS (Corning, MT35010CV). DKOcr298 was cultured in phenol red–free DMEM/high glucose (Gibco, 31053-028) with L-glutamine (Gibco, 35050-061), sodium pyruvate (Gibco, 11360-070), and 10% charcoal-stripped FBS (MilliporeSigma, F6765). NCI-H660 cells (23) (CRL-5813) were purchased from ATCC and cultured according to their recommendation. LNCaP_FGC cells were purchased from ATCC (CRL-1740). V16D (28) was a gift from A. Zoubeidi (University of British Columbia, Vancouver, British Columbia, Canada). LNCaP_FGC and V16D cells were grown in RPMI1640 (Gibco, 11875-093) supplemented with 10% FBS (Corning, MT35010CV). The LTL331R cell line was derived from LTL331R PDX (26) and grown in RPMI1640 (Gibco, 11875-093) with 1× insulin, transferrin, selenium, and ethanolamine solution (ITS-X; Gibco, 51500-056) plus 5% FBS (Corning, MT35010CV). HCT116 DKO (42) was a gift from B. Vogelstein (Johns Hopkins Medicine, Baltimore, Maryland, USA). All models were validated with STR DNA fingerprinting by Genetica Cell Line Testing (a LabCorp brand) and routinely tested for mycoplasma contamination using a MycoAlert Mycoplasma Detection kit (Lonza, LT07-318).

### Drug information.

ZEN-3694 was obtained from Zenith Epigenetics. JQ1 (HY-13030) and 5-aza-2’-deoxycytidine (decitabine) (HY-A0004) were obtained from MedChemExpress. All drugs were dissolved in DMSO, and DMSO was used as a vehicle control for in vitro treatment studies.

### Gene set ORA.

For multiomic integrative studies, functional enrichment analysis of DEGs was performed using clusterProfiler package v4.9.0.002 (43). DEGs were defined as those with fold-change greater than 1.5 or less than –1.5 and an FDR less than 0.05. Functional annotation and gene categorization were performed using Gene Ontology (GO) terms (44, 45). To enhance annotation coverage, gene sets were retrieved from the Molecular Signatures Database (MSigDB) using the msigdbr package v7.5.1 (46). Additional annotation was obtained from the org.Mm.eg.db package v3.16.0 (https://bioconductor.org/packages/release/data/annotation/html/org.Mm.eg.db.html). ORA was conducted by comparing the DEG list to a background set of all expressed genes identified in the RNA-Seq dataset. Statistical significance was assessed using hypergeometric testing, and *P* values were adjusted for multiple testing using the Benjamini-Hochberg method, with a significance threshold of FDR less than 0.1. The ORA for ZEN plus dAZA combination treatment gene expression analysis was performed using WebGestaltR version 0.4.6 (47–50), using protein-coding genes with adjusted *P* less than 0.05 and absolute shrunkLFC greater than log_2_(1.5) or less than –log_2_(1.5). The enrichDatabaseFile (.gmt) files for the various gene sets (Hallmark, Biological Process, and Chemical and Genetic Perturbations) were downloaded from the GSEA MSigDB (46) version 2022.1, and “genome” was used as the reference set.

### Differentiation signature enrichment analysis.

Differentiation signature enrichment was performed with VIPER v1.34.0 (33) as previously described (51) using luminal (6, 34) and neuroendocrine (13) phenotypic signatures.

### BETi dose response.

Cells were treated for 72 hours in biological triplicate with a 7-point, 5-fold dilution series from 10 μM ZEN-3694 or 10 μM JQ1 dissolved in DMSO. Cell viability was assessed using the CellTiter-Glo assay (Promega, G7570) using vehicle-treated cells for normalization and fitted to a logistic curve as described previously (52).

### MSPCR.

Genomic DNA from 3 biological replicates was extracted using AllPrep RNA/DNA mini kits (QIAGEN, 80204) and bisulfite-treated using the EZ DNA Methylation kit (Zymo Research, D5001) according to the manufacturer’s instructions using 500–1000 ng of DNA. MSPCR reactions were performed in 25 μL total volume using 1.25 U of Platinum Taq polymerase (Invitrogen, 15966005), 200 μM dNTPs (Invitrogen, 18427088), 500 nM forward and reverse primers (IDT; primer sequences detailed in Supplemental Table 20; *GSTP1* and *RARB2* primers were described previously; refs. 53, 54), and 30–50 ng of bisulfite-treated DNA as template. MSPCR reactions were carried out with the following conditions: 94°C for 2 minutes (denaturation); 35 cycles of 94°C for 15 seconds (disassociation); 60°C for 15 seconds (annealing); 68°C for 10 seconds (extension); 68°C for 30 seconds (final extension); and hold at 4°C. PCR amplicons were run on a 3% agarose gel and scanned using a ChemiDoc MP Imaging System (Bio-Rad) for analysis. Negative control reactions were performed using only water as template. Densitometry analysis was performed using ImageJ (NIH) version 1.54p (55).

### Long-term BETi and DNMTi treatment experiments/colony formation assays.

Cells were plated and pretreated with vehicle, 500 nM ZEN-3694, 100 nM dAZA, or combination ZEN plus dAZA daily for 72 hours in biological triplicate. After the 72-hour daily treatments, cells were harvested and counted using trypan blue exclusion and a Countess 3 cell counter (Invitrogen), and equal numbers of pretreated cells were plated for continued ZEN-3694 treatment. For suspension cell lines (NCI-H660 and LTL331R), cells were continually grown in suspension and dosed with 500 nM ZEN or ZEN-vehicle, depending on what they were treated with during the 72-hour pretreatment, every 72 hours for a total of 6 days. Cells were then harvested and counted using trypan blue exclusion as a readout for cell viability. For adherent cell lines (TKO and DKOcr298), equal numbers of pretreated cells were trypsinized, harvested, and plated for colony formation assays on 10 cm dishes in technical triplicates for every biological replicate for 24 hours, for a total of 9 replicates per treatment group. After plating for 24 hours, cells were treated with either 500 nM ZEN-3694 or ZEN-vehicle, depending on what they were treated with during the 72-hour pretreatment, every 72 hours for the indicated time. Colonies were then fixed and stained by adding 6% glutaraldehyde (Sigma-Aldrich, G6257)/0.05% crystal violet (Fisher Chemical, C58125) solution and incubating for 30 minutes (56). Colony formation assays were scanned using a ChemiDoc MP Imaging System (Bio-Rad). Colonies were counted using ImageJ (NIH) version 1.54p (55) by setting the signal threshold to 30–150 and using the Analyze Particles function with the following parameters: size: 35–infinity; circularity: 0.35–1.00.

### EdU incorporation assay.

Cell proliferation was measured by EdU incorporation using Click-iT Plus EdU Alexa Fluor 647 Flow Cytometry Assay kit (Thermo Fisher Scientific, C10634) according to manufacturer’s instructions. Briefly, cells were treated with vehicle, 500 nM ZEN, 100 nM dAZA, or the combination daily for 48 hours (TKO/DKOcr298) or 72 hours (LTL331R/NCI-H660) in biological triplicates and then pulsed with 10 μM EdU for 1 hour (TKO), 2 hours (DKOcr298), or 3 hours (LTL331R/NCI-H660). Cells were then fixed, permeabilized, and stained with Click-iT Plus detection cocktail. The cells were analyzed by LSRFortessa II (BD Biosciences) for the percentage of EdU-positive cells. Cells without EdU incorporation that were processed as above were used to gate the EdU-positive population. The percentage of EdU-positive cells was analyzed using FlowJo version 10.10.0 and plotted using GraphPad Prism version 10.2.3.

### Apoptosis assay.

Apoptosis was measured using annexin V (BD Biosciences, 550475) according to the manufacturer’s instructions. Briefly, cells were treated with vehicle, 500 nM ZEN, 100 nM dAZA, or the combination daily for 48 hours (TKO/DKOcr298) or 72 hours (LTL331R/NCI-H660) in biological triplicates and then trypsinized, harvested, and stained with 5 μL APC-conjugated annexin V and 5 μL propidium iodide (Sigma-Aldrich, P4864) for 15 minutes. Cells were analyzed by LSRFortessa II (BD Biosciences) for APC (annexin V) or propidium iodide signal. Unstained and single-stained (annexin V-APC or propidium iodide) cells were used to gate stained populations. The percentage of apoptotic cells was analyzed using FlowJo version 10.10.0 and plotted using GraphPad Prism version 10.2.3.

### Synergy analysis.

A potential synergistic effect of combination treatment was determined through the Bliss independence model (22). The model assumes the 2 drugs are independently effective, and the combination effect is calculated with the formula: IAB = IA + IB – IA × IB, where IA and IB are the inhibitory effect of a single drug at a given concentration. When the difference between the experimentally determined combination and the expected effect (IAB) is between –0.1 and 0.1, it is deemed additive (–0.1 < Bliss Index < 0.1); greater than 0.1, it is deemed synergistic (Bliss Index > 0.1); lower than –0.1, it is deemed antagonistic (Bliss Index < –0.1). The synergy analysis was performed using 6 biological replicates per cell line/treatment condition treated daily for 72 hours.

### IHC.

IHC was performed on 4 μm FFPE tissue sections. All IHC steps were performed on a Ventana Discovery Ultra automatic staining platform. Briefly, after deparaffinization, heat-induced epitope retrieval was performed using cell conditioning media CC1 and CC2. Sections were incubated with primary antibodies against Ki-67 (Roche, 790-4286) or cleaved PARP (cl-PARP) (Cell Signaling Technology, 5625S human; Cell Signaling Technology, 94885S mouse). Ventana Discovery OmniMap detection kit (anti-rabbit: 760-4311, anti-mouse: 760-4310) and Ventana ChromoMap DAB kit (Roche, 760-159) was used to develop the IHC signal. Counterstaining was performed using hematoxylin (hematoxylin II, Ventana Medical Systems, Roche, 790-2208). For estimation of the proliferative index, 500 tumor cells across 5 hotspot areas from 3 biological replicates were assessed for Ki-67 positivity and expressed as a percentage. cl-PARP-positive cells from 3 biological replicates were expressed as number of positive cells/10 high-power field. IHC images were captured using Zeiss Axioimager 2 brightfield upright microscope and Zeiss Axiocam 705 color camera.

### In vivo BETi and DNMTi antitumor activity.

All experiments were performed using 6- to 8-week-old male athymic homozygous nude Foxn1^nu^ mice (The Jackson Laboratory, 002019). For TKO studies, 2 × 10^5^ TKO cells were prepared in a 1:1 mixture of growth media and Matrigel (Corning, 356234) and subcutaneously injected into mouse flanks. For LTL331R PDX (26) studies, tumors were subcutaneously implanted into mouse flanks. When tumors reached an average volume of 150 mm^3^ for TKO or 200 mm^3^ for LTL331R, mice were randomly assigned to one of the following treatment groups: control, ZEN-3694, dAZA, or combination ZEN-3694 and dAZA. ZEN-3694 (a proprietary formulation from Zenith Epigenetics, Calgary, Alberta, Canada) was administered daily via oral gavage at a dose of 50 mg/kg. dAZA 0.4mg/kg (TKO) or 0.8 mg/kg (LTL331R) was administered in PBS daily for 5 days followed by a 9-day holiday. Tumor volumes and body weights were measured twice per week. Animals were euthanized before treatment completion if tumors reached the humane endpoint. All remaining animals were euthanized on day 30 of both TKO and LTL331R PDX studies. Blood samples were collected from each animal on day 30 of both studies, and complete blood cell counts were performed by the University of Michigan ULAM Pathology Core. Bone marrow was isolated from harvested femurs at the indicated time point using 500 μL PBS flushed through the bone.

### Statistics.

For analyses of cell viability, gene expression, and IHC, statistical significance was assessed using 1-way ANOVA with Dunnett’s or Benjamini-Hochberg correction, with adjusted *P* value less than 0.05 considered significant. ORA was conducted using a hypergeometric test followed by Benjamini-Hochberg correction, applying an FDR threshold of less than 0.1 to identify significantly overrepresented gene sets among DEGs. In vivo tumor growth and body weight measurements were analyzed using 2-way ANOVA with Tukey’s multiple-comparison test, using a significance threshold of adjusted *P* less than 0.05. Except for animal studies, experiments were not randomized.

### Study approval.

All animal studies were conducted in accordance with the guidelines of the University of Michigan’s IACUC, which approved these studies (protocol PRO00011266).

### Data availability.

Raw sequencing and processed data generated in this study have been deposited in the NCBI’s Gene Expression Omnibus under the accession codes GSE309033 and GSE309034 (RNA-Seq), GSE311400 (ATAC-Seq), GSE311321 and GSE311403 (ERRBS), and GSE311404 (ChIP-Seq). Cancer Dependency Map data can be accessed via https://depmap.org/portal Raw data associated with all graphs in the figures can be accessed in the Supporting Data Values file. Unedited scans of agarose gels used in figures are provided in the unedited gel images file.

## Author contributions

WKS, DF, AK, ZD, JAY, and JJA designed the research. WKS, DF, AK, ZD, SC, CZ, ER, JAK, and JAY performed experiments. WKS, DF, AK, ZD, SC, CZ, ER, DK, OAS, JAK, CRA, HNB, and JAY acquired data. RM, SM, and AMU performed IHC evaluations. DF, KB, RGC, CC, FZ, YMH, ZX, RJR, and MC performed computational analyses. WKS, DF, AK, ZD, and JJA analyzed the data. PSN, LE, and YW provided cell line or PDX models. WKS, DF, and JJA wrote the manuscript. WKS, DF, AK, ZD, SC, ER, AMC, JAK, PSN, CM, MCH, LE, JAY, and JJA revised the manuscript. The order of the co–first authors was determined based on their relative contributions to this study. All authors read and approved the final manuscript.

## Conflict of interest

JJA has received consulting fees from Fortis Therapeutics and ORIC Pharmaceuticals and research support to his institution from Beactica Therapeutics, Zenith Epigenetics, and a National Comprehensive Cancer Network/Astellas Pharma Global Development Inc./Pfizer Inc. research award. MCH served as a paid consultant/received honoraria from Pfizer, K36, Genentech, and AstraZeneca and has received research funding from Merck, Novartis, Genentech, PromiCell, XYone Therapeutics, and Bristol Myers Squibb. PSN has served as a paid advisor to Genentech, AstraZeneca, and Vesto Therapeutics and received research support from Janssen for work unrelated to the present studies.

## Funding support

This work is the result of NIH funding, in whole or in part, and is subject to the NIH Public Access Policy. Through acceptance of this federal funding, the NIH has been given a right to make the work publicly available in PubMed Central. This content is solely the responsibility of the authors and does not necessarily represent the official views of the funders.

- National Cancer Institute (NCI) R01CA291986, R01CA251245, and R01CA282005 (to JJA).
- Michigan Prostate SPORE NCI P50CA186786 (to JJA).
- University of Michigan Rogel Cancer Center NCI P30CA046592 (to JJA).
- Joint Institute for Cancer Research Award (to JJA).
- Prostate Cancer Foundation Challenge Award (to JJA).
- The Allen Family and Smith Family (to JJA and AK).
- Sheppard Family Foundation Sheppard Scholar Award (to WKS).
- Prostate Cancer Foundation Young Investigator Award (to WKS).
- NCI P30CA046392 (to KB).
- Department of Defense Idea Award W81XWH2110539 (to ZX).
- National Institute of General Medical Sciences R01GM147365 (to ZX).
- A Silver Family Innovation Foundation Award (to ZX).
- Postdoctoral Fellowship of Portland Oral Health Research Training (PORT) program NIH T90DE030859 (to YMH).
- NIH P50CA097186, NIH P01CA298991, and NIH R01CA266452 (to PSN).
- Department of Defense PC230420 (to PSN).
- NCI R01CA252468 (to LE).

## Supplementary Material

Supplemental data

Unedited blot and gel images

Supplemental table 1

Supplemental table 10

Supplemental table 11

Supplemental table 12

Supplemental table 13

Supplemental table 14

Supplemental table 15

Supplemental table 16

Supplemental table 17

Supplemental table 18

Supplemental table 19

Supplemental table 2

Supplemental table 20

Supplemental table 3

Supplemental table 4

Supplemental table 5

Supplemental table 6

Supplemental table 7

Supplemental table 8

Supplemental table 9

Supporting data values

## Version 1 08/11/2026

In-Press Preview

## Version 2 09/22/2026

Electronic publication
