# Supplementary material for: Combined BET bromodomain and DNA methyltransferase inhibition targets critical survival pathways in transdifferentiated prostate cancer
Source: JCI Insight. 2026 Aug 11;11(18):e207543. doi: 10.1172/jci.insight.207543 (PMC13596728; doi:10.1172/jci.insight.207543)
Supplement: Supplementary file 1 [file jciinsight-11-207543-s145.pdf]

Supplemental Material

Combined BET bromodomain and DNMT inhibition targets critical survival pathways in transdifferentiated prostate cancer

William K. Storck,[1,2,†] Diana Flores,[1,2,†] Anbarasu Kumaraswamy,[1,2] Zhi Duan,[1,2] Shrabastee Chakraborty,[1,2] Chao Zhang,[1,2] Eva Rodansky,[1,2] Dhruv Khokhani,[1,2,3] Olivia A. Swaim,[1,2,3] Karan Bedi,[2,4] Raymond G. Cavalcante,[5] Canping Chen,[6,7] Faming Zhao,[6,7] Ya-Mei Hu,[6,7] Zheng Xia,[6,7] Ryan J. Rebernick,[8,9] Marcin Cieslik,[2,9,10] Rahul Mannan,[8,10] Somnath Mahapatra,[8,10] Arul M. Chinnaiyan,[2,8,9,10,11,12] Aaron M. Udager,[8,10] Joshua A. Kuleape,[1,2] Catherine R. Alumkal,[1,2] Hannah N. Beck,[1,2] Peter S. Nelson,[13,14,15,16] Colm Morrissey,[13] Michael C. Haffner,[14,15,16] Leigh Ellis,[17,18,19] Yuzhuo Wang,[20,21,22] Joel A. Yates,[1,2] and Joshi J. Alumkal[1,2,8,*]

Affiliations:
[1]Department of Internal Medicine, University of Michigan, Ann Arbor, Michigan, USA
[2]Rogel Cancer Center, University of Michigan, Ann Arbor, Michigan, USA
[3]College of Literature, Science, and the Arts, University of Michigan, Ann Arbor, Michigan, USA
[4]Department of Biostatistics, School of Public Health, University of Michigan, Ann Arbor, Michigan, USA
[5]Bioinformatics Core, University of Michigan, Ann Arbor, Michigan, USA
[6]Department of Biomedical Engineering, Oregon Health & Science University, Portland, Oregon, USA
[7]Knight Cancer Institute, Oregon Health & Science University, Portland, Oregon, USA
[8]Michigan Center for Translational Pathology, University of Michigan, Ann Arbor, Michigan, USA
[9]Department of Computational Medicine and Bioinformatics, University of Michigan, Ann Arbor, Michigan, USA
[10]Department of Pathology, University of Michigan, Ann Arbor, Michigan, USA
[11]Howard Hughes Medical Institute, University of Michigan, Ann Arbor, Michigan, USA
[12]Department of Urology, University of Michigan, Ann Arbor, Michigan, USA
[13]Department of Urology, University of Washington, Seattle, Washington, USA
[14]Division of Human Biology, Fred Hutchinson Cancer Center, Seattle, Washington, USA
[15]Division of Clinical Research, Fred Hutchinson Cancer Center, Seattle, Washington, USA
[16]Department of Laboratory Medicine and Pathology, University of Washington, Seattle, Washington, USA
[17]Center for Prostate Disease Research, Murtha Cancer Center Research Program, Department of Surgery, Uniformed Services University of the Health Sciences, Bethesda, Maryland, USA
[18]The Henry M. Jackson Foundation for the Advancement of Military Medicine, Inc., Bethesda, Maryland, USA
[19]Genitourinary Malignancies Branch, Center for Cancer Research, National Cancer Institute, Bethesda, Maryland, USA
[20]Vancouver Prostate Centre, Vancouver, British Columbia, Canada
[21]Department of Urologic Sciences, Faculty of Medicine, University of British Columbia, Vancouver, British Columbia, Canada
[22]BC Cancer Research Institute, BC Cancer, Vancouver, British Columbia, Canada
[†]These authors contributed equally
*Corresponding Author

Address correspondence to: Dr. Joshi J. Alumkal, Department of Internal Medicine, Hematology/Oncology, University of Michigan, 7312 Rogel Cancer Center, 1500 East Medical Center Dr, Ann Arbor, Michigan 48109, USA. Telephone: +1.734.936.9868; Email: jalumkal@med.umich.edu.

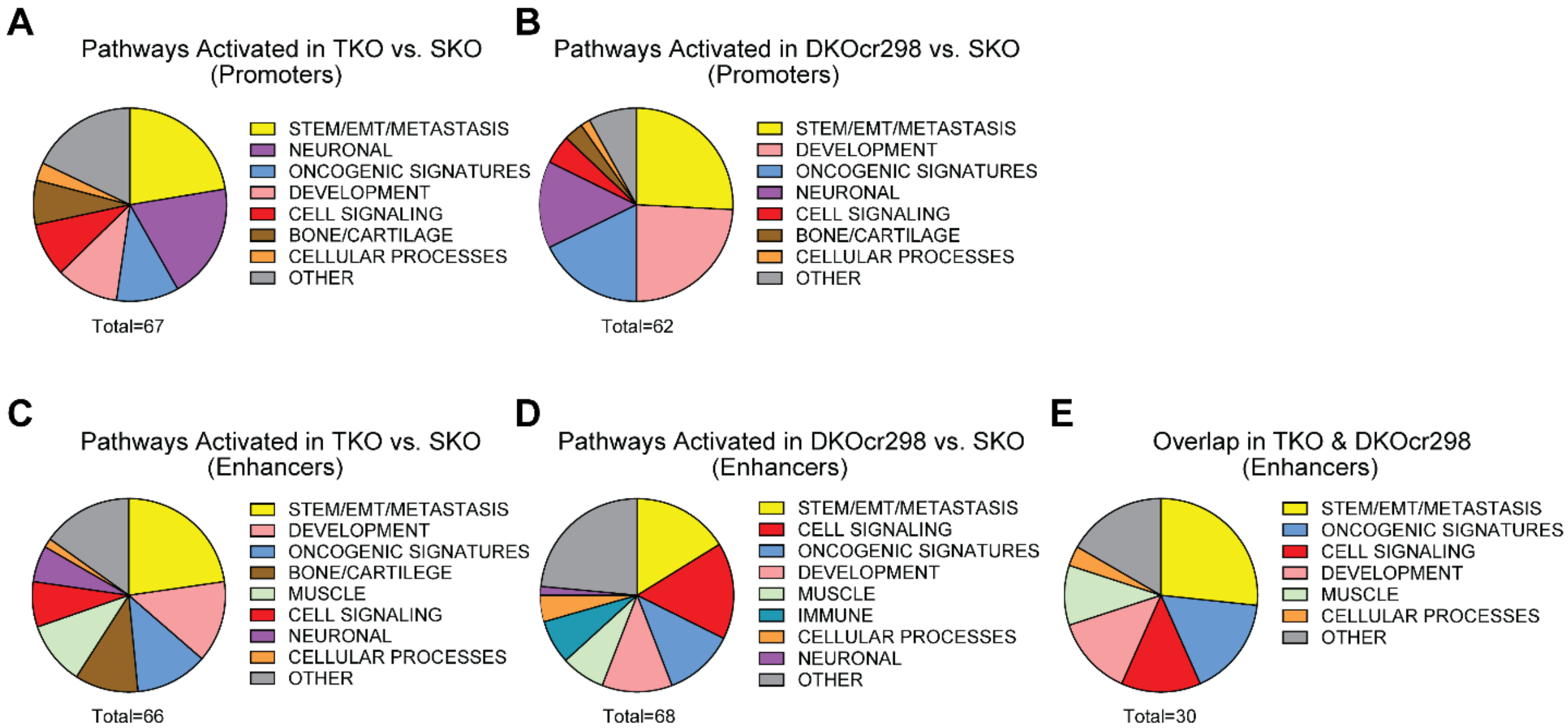

Supplemental Figure 1. *Trp53/Rb1* loss activates stemness, EMT, metastasis, and neuronal genes.

(**A** and **B**) Categorization of top activated gene sets (FDR < 0.1) from MSigDB Hallmark, CGP, and GOBP gene set collections in TKO (**A**) and DKOcr298 (**B**) cell lines that are enriched in activated genes with hyperaccessible promoter regions.

(**C** and **D**) Categorization of top activated gene sets (FDR < 0.1) from MSigDB Hallmark, CGP, and GOBP gene set collections in TKO (**C)** and DKOcr298 (**D**) cell lines that are enriched in activated genes with hyperaccessible enhancer regions.

(**E**) Categorization of top shared activated gene sets (FDR < 0.1) from MSigDB Hallmark, CGP, and GOBP gene set collections between TKO and DKOcr298 cell lines that are enriched in activated genes with hyperaccessible enhancer regions.

Statistical significance in **A**-**E** was determined using hypergeometric test followed by Benjamini-Hochberg correction.

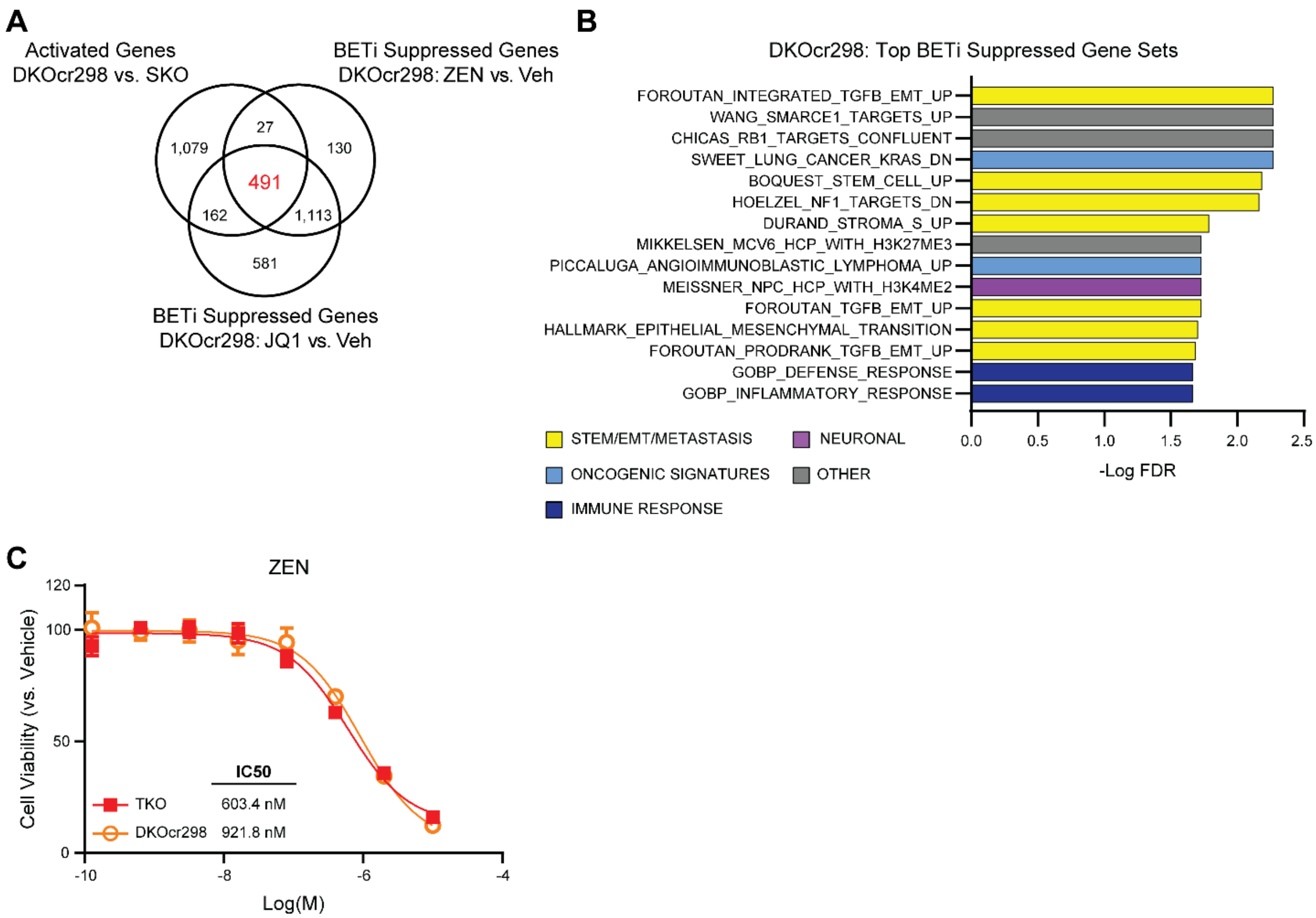

Supplemental Figure 2. BETi blocks induction of stemness, EMT, and metastasis pathways in *Trp53/Rb1*-deficient tumors and is growth suppressive.

(**A**) Venn diagram of genes activated by *Trp53*/*Rb1* loss with hyperaccessible promoter regions in DKOcr298 vs. SKO but suppressed (fold change < 1.5; *P*adj < 0.05) with 24-hr treatment with 1 μM of the BET bromodomain inhibitor ZEN-3694 (ZEN) or 24-hr treatment with 500 nM of the BET bromodomain inhibitor JQ1 in DKOcr298. (*n* = 3 for each treatment condition).

(**B**) Top gene sets (FDR < 0.1) from MSigDB Hallmark, CGP, and GOBP gene set collections enriched in the intersection of 491 genes are shown. Statistical significance was determined using hypergeometric test followed by Benjamini-Hochberg correction.

(**C**) Dose response viability curves of TKO and DKOcr298 treated with ZEN for 72 hrs measured by CTG (*n* = 4 for all treated samples). Calculated IC50 values are shown.

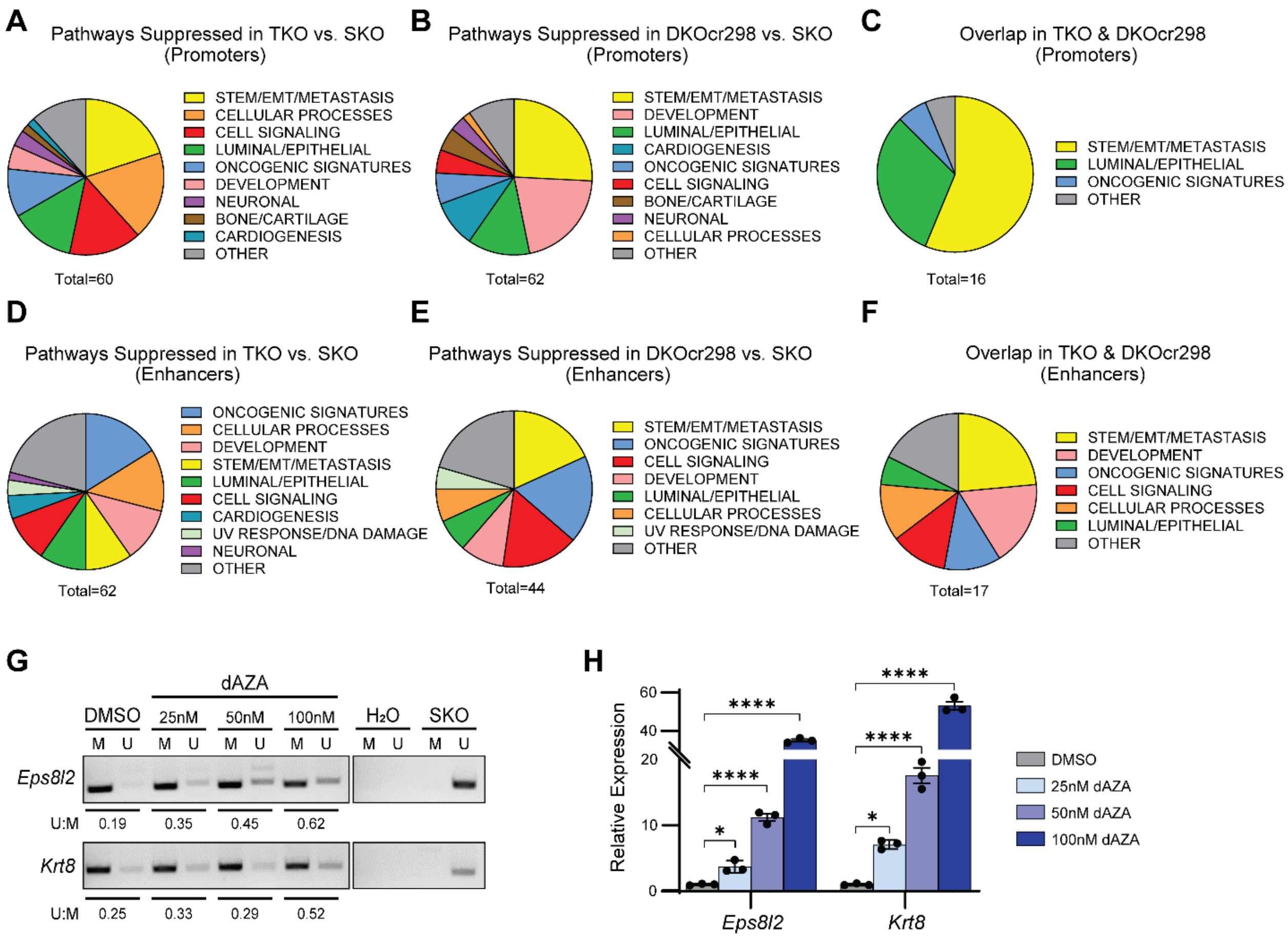

Supplemental Figure 3. ***Trp53/Rb1*** **loss leads to widespread chromatin hypoaccessibility and DNA methylation changes that suppress epithelial gene expression.**

(**A** and **B**) Categorization of top suppressed gene sets (FDR < 0.1) from MSigDB Hallmark, CGP, and GOBP gene set collections in TKO (**A**) and DKOcr298 (**B**) cell lines that are enriched in suppressed genes with hypoaccessible and hypermethylated promoter regions.

(**C**) Categorization of top shared suppressed gene sets (FDR < 0.1) from MSigDB Hallmark, CGP, and GOBP gene set collections between TKO and DKOcr298 cell lines that are enriched in suppressed genes with hypoaccessible and hypermethylated promoter regions.

(**D** and **E**) Categorization of top suppressed gene sets (FDR < 0.1) from MSigDB Hallmark, CGP, and GOBP gene set collections in TKO (**D**) and DKOcr298 (**E**) cell lines that are enriched in suppressed genes with hypoaccessible and hypermethylated enhancer regions.

(**F**) Categorization of top shared suppressed gene sets (FDR < 0.1) from MSigDB Hallmark, CGP, and GOBP gene set collections between TKO and DKOcr298 cell lines that are enriched for suppressed genes with hypoaccessible and hypermethylated enhancer regions.

Statistical significance in **A**-**F** was determined using hypergeometric test followed by Benjamini-Hochberg correction.

(**G**) Methylation-specific PCR (MSPCR) analysis of *Trp53*/*Rb1* loss-induced hypermethylated and silenced genes in DKOcr298 treated with increasing doses of the DNA methyltransferase inhibitor 5-aza-2'-deoxycytidine (dAZA). Cells were treated daily for 72 hrs. Methylated (M) and unmethylated (U) specific amplicons are shown for each indicated sample. The densitometry ratio of unmethylated to methylated products (U:M) is shown below their respective amplicon pairs. SKO is included as an unmethylated control. Representative samples from biological triplicates are shown for each condition.

(**H**) RNA-qPCR of *Trp53*/*Rb1* loss-induced hypermethylated and silenced genes in DKOcr298 with increasing doses of dAZA. RNAs used in this analysis were derived from the same samples as in (**G**). Data presented are the mean ± SD (n = 3 for all treated samples). Statistical significance was determined using one-way ANOVA with Dunnett's multiple-comparison test. *P < 0.05; ****P < 0.0001.

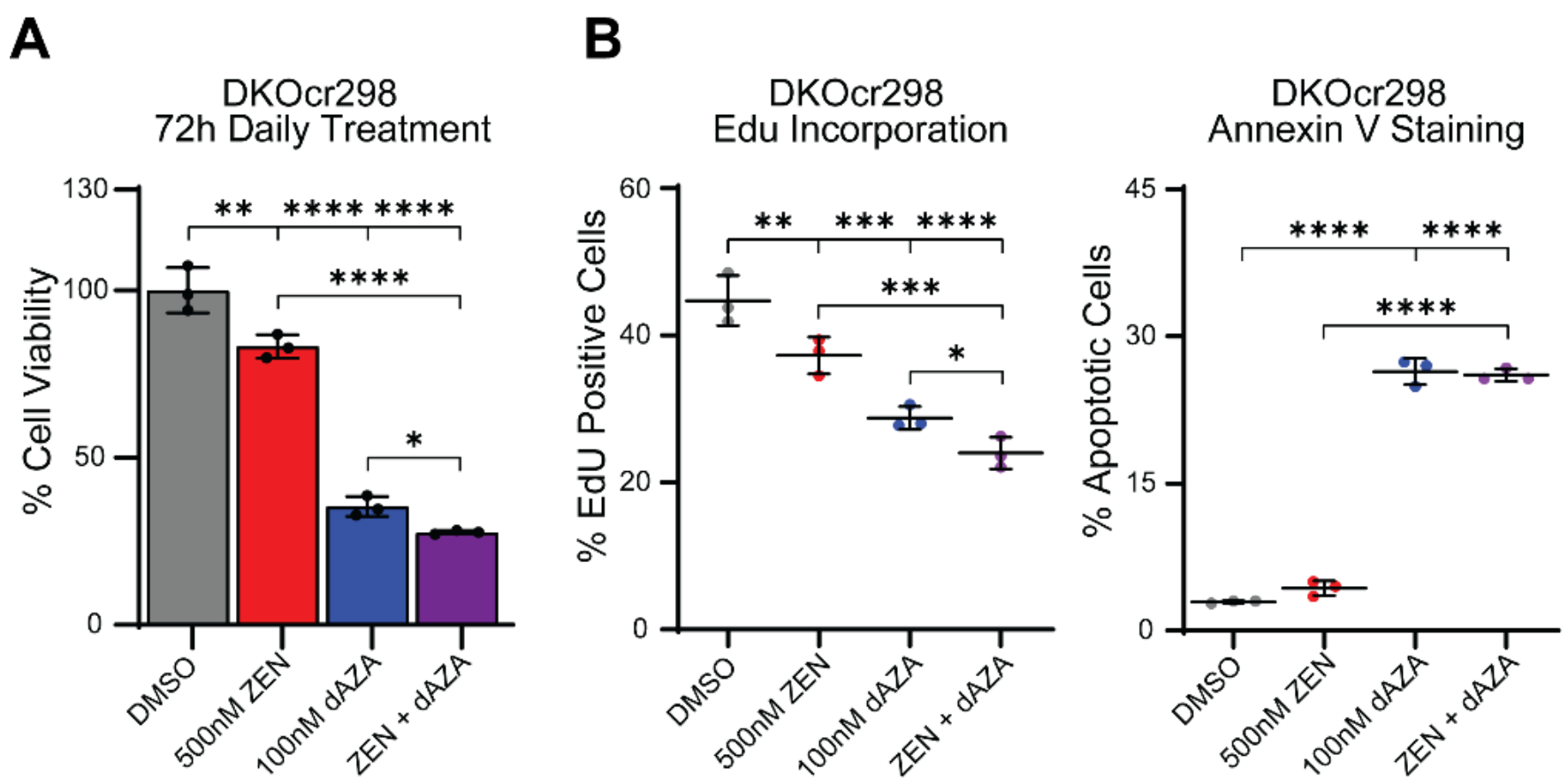

Supplemental Figure 4. BETi and DNMTi combination treatment leads to superior growth suppression in *Trp53/Rb1*-deficient cells in vitro.

(**A**) Cell count viability of DKOcr298 cells after 72-hr daily treatment with indicated dose of ZEN alone, dAZA alone, or ZEN + dAZA combination. Data presented are the mean ± SD (*n* = 3 for all treated samples). Statistical significance was determined using one-way ANOVA with Benjamini-Hochberg multiple testing correction. *$P < 0.05$; **$P < 0.01$; ****$P < 0.0001$.

(**B**) EdU incorporation and apoptosis assays using DKOcr298 cells after 48-hr daily treatment with indicated dose of ZEN alone, dAZA alone, or ZEN + dAZA combination. Data presented are the mean ± SD (*n* = 3 for all treated samples). Statistical significance was determined using one-way ANOVA with Benjamini-Hochberg multiple testing correction. *$P < 0.05$; **$P < 0.01$; ***$P < 0.001$; ****$P < 0.0001$.

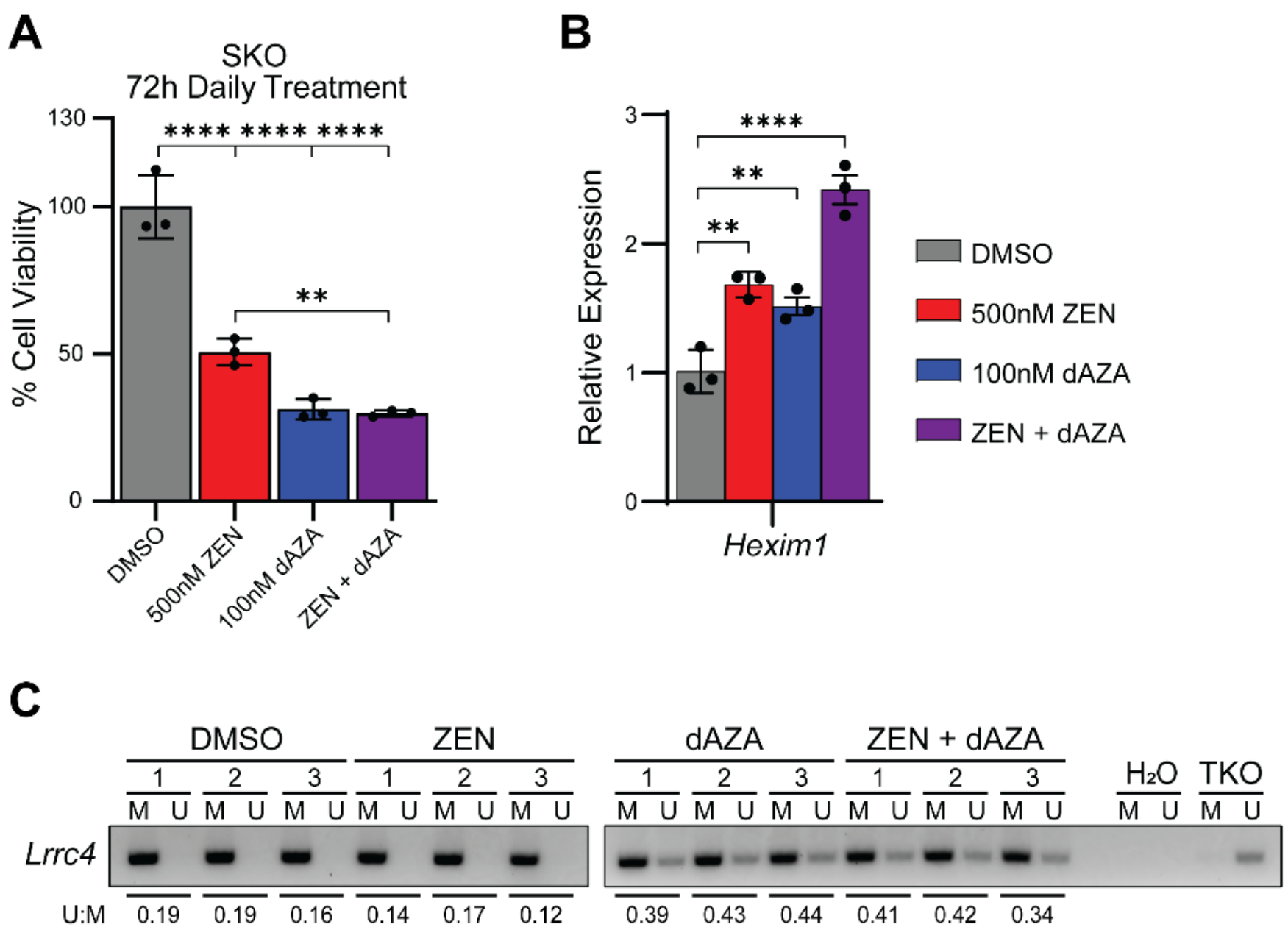

Supplemental Figure 5. ZEN + dAZA combination treatment in SKO.

(**A**) Cell count viability of SKO (*Pten*-null only) cells after 72-hr daily treatment with indicated dose of ZEN alone, dAZA alone, or ZEN + dAZA combination. Data presented are the mean ± SD (n = 3 for all treated samples). Statistical significance was determined using one-way ANOVA with Benjamini-Hochberg multiple testing correction. **$P < 0.01$; ****$P < 0.0001$.

(**B**) RNA-qPCR of BETi-activated gene *Hexim1* (1) in SKO treated with indicated dose of ZEN alone, dAZA alone, or ZEN + dAZA combination daily for 72 hrs. Data presented are the mean ± SD (n = 3 for all treated samples). Statistical significance was determined using one-way ANOVA with Dunnett's multiple-comparison test. **$P < 0.01$; ***$P < 0.001$.

(**C**) MSPCR analysis of the hypermethylated gene *Lrrc4* in SKO treated with indicated dose of ZEN alone, dAZA alone, or ZEN + dAZA combination daily for 72 hrs. Methylated (M) and unmethylated (U) specific amplicons are shown for each indicated sample. The densitometry ratio of unmethylated to methylated products (U:M) is shown below their respective amplicon pairs.

TKO is included as an unmethylated control. DNAs used in this analysis were derived from the same samples as in (**B**).

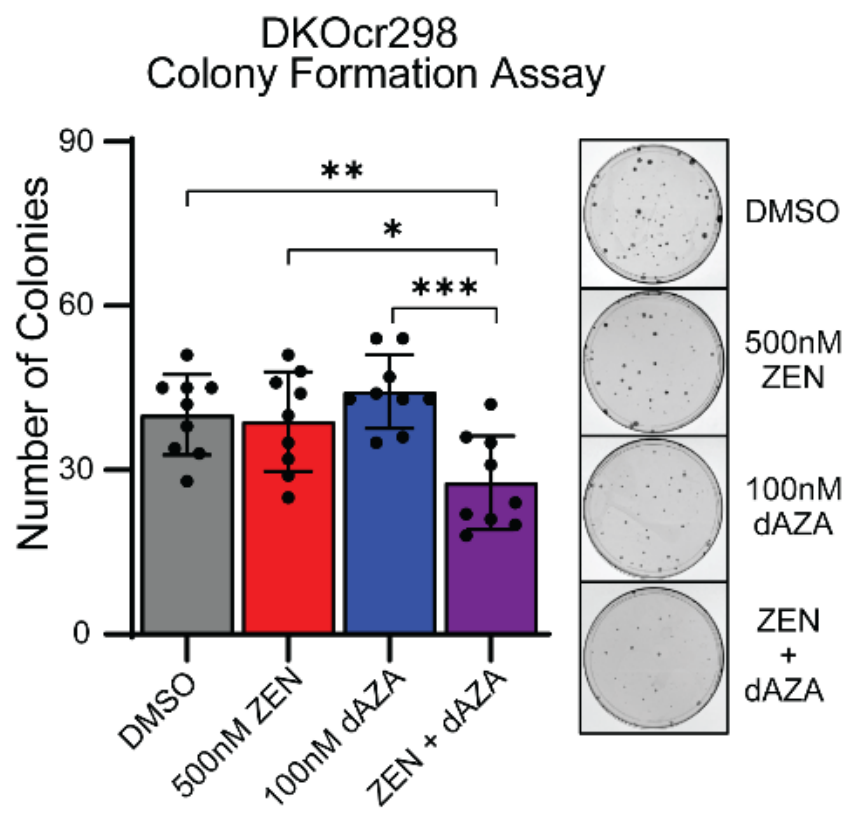

Supplemental Figure 6. Long term ZEN + dAZA combination treatment in DKOcr298.

DKOcr298 colony formation assay (CFA) colony counts after long term treatments. Cells were treated with indicated doses of ZEN, dAZA, or the combination for 72 hrs daily before plating followed by sustained ZEN or ZEN-vehicle treatment without dAZA for 11 days. Data presented are the mean ± SD (*n* = 9 for all treated samples). Statistical significance was determined one-way ANOVA with Benjamini-Hochberg multiple testing correction. *$P < 0.05$; **$P < 0.01$; ***$P < 0.001$. Representative CFA scans are shown on the right.

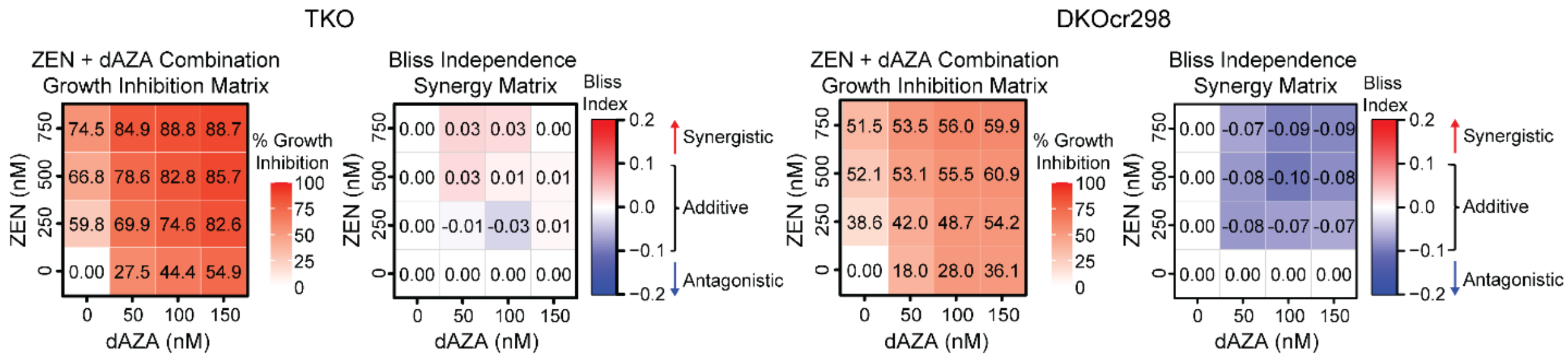

Supplemental Figure 7. ZEN + dAZA Bliss synergy analysis in TKO and DKOcr298.

Percent growth inhibition and Bliss synergy combination index matrices for 72-hr daily ZEN and dAZA treatments in TKO and DKOcr298 (> 0.1 synergistic; between 0.1 and -0.1 additive; < -0.1 antagonistic) (*n* = 6 for all treated samples). Percent growth inhibition and Bliss synergy index values are shown within each respective intersection.

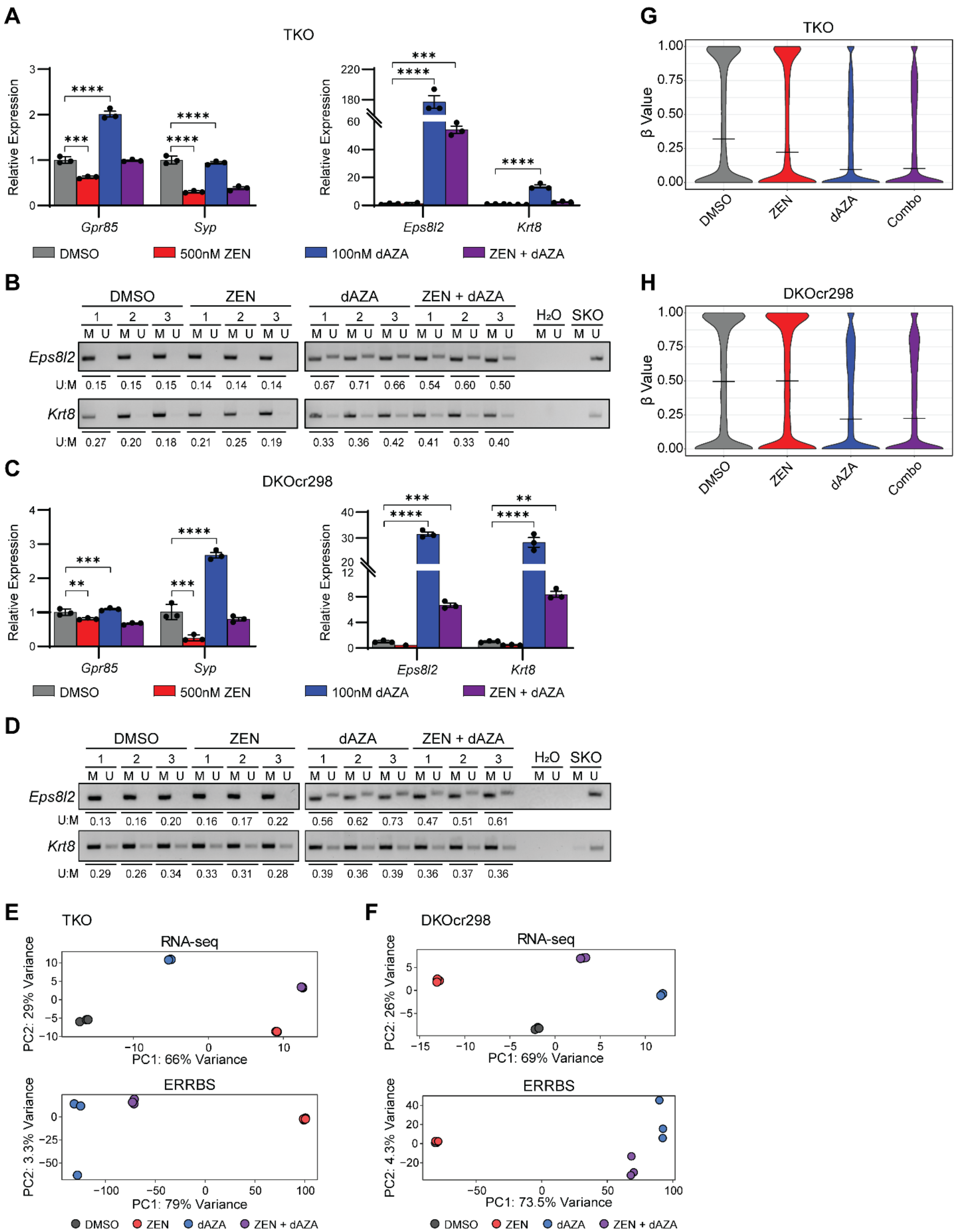

A
TKO
Relative Expression
Gpr85
Syp
Eps8l2
Krt8
DMSO
500nM ZEN
100nM dAZA
ZEN + dAZA
B
DMSO
ZEN
dAZA
ZEN + dAZA
H2O
SKO
Eps8l2
Krt8
U:M
0.15 0.15 0.15 0.14 0.14 0.14 0.67 0.71 0.66 0.54 0.60 0.50
0.27 0.20 0.18 0.21 0.25 0.19 0.33 0.36 0.42 0.41 0.33 0.40
C
DKOcr298
D
0.13 0.16 0.20 0.16 0.17 0.22 0.56 0.62 0.73 0.47 0.51 0.61
0.29 0.26 0.34 0.33 0.31 0.28 0.39 0.36 0.39 0.36 0.37 0.36
E
TKO
RNA-seq
PC2: 29% Variance
PC1: 66% Variance
ERRBS
PC2: 3.3% Variance
PC1: 79% Variance
DMSO
ZEN
dAZA
ZEN + dAZA
F
DKOcr298
RNA-seq
PC2: 26% Variance
PC1: 69% Variance
ERRBS
PC2: 4.3% Variance
PC1: 73.5% Variance
G
TKO
β Value
DMSO
ZEN
dAZA
Combo
H
DKOcr298

Supplemental Figure 8. Gene expression and DNA methylation changes from ZEN + dAZA combination treatment in TKO and DKOcr298.

(**A**) RNA-qPCR of BETi-suppressed genes (*Gpr85; Syp*) and dAZA-activated genes (*Eps8l2*; *Krt8*) in TKO treated with indicated dose of ZEN alone, dAZA alone, or ZEN + dAZA combination daily for 72 hrs. Data presented are the mean ± SD (n = 3 for all treated samples). Statistical significance was determined using one-way ANOVA with Dunnett's multiple-comparison test. ***P < 0.001; ****P < 0.0001.

(**B**) MSPCR analysis of *Trp53*/*Rb1* loss-induced hypermethylated and silenced genes in TKO treated with indicated dose of ZEN alone, dAZA alone, or ZEN + dAZA combination daily for 72 hrs. Methylated (M) and unmethylated (U)-specific amplicons are shown for each indicated sample. The densitometry ratio of unmethylated to methylated products (U:M) is shown below their respective amplicon pairs. SKO is included as an unmethylated control. DNAs used in this analysis were derived from the same samples as in (**A**).

(**C**) RNA-qPCR of BETi-suppressed genes (*Gpr85*; *Syp*) and dAZA-activated genes (*Eps8l2*; *Krt8*) in DKOcr298 treated with indicated dose of ZEN alone, dAZA alone, or ZEN + dAZA combination daily for 72 hrs. Data presented are the mean ± SD (n = 3 for all treated samples). Statistical significance was determined using one-way ANOVA with Dunnett's multiple-comparison test. ***P < 0.001; ****P < 0.0001.

(**D**) MSPCR analysis of *Trp53*/*Rb1* loss-induced hypermethylated and silenced genes in DKOcr298 treated with indicated dose of ZEN alone, dAZA alone, or ZEN + dAZA combination daily for 72 hrs. Methylated (M) and unmethylated (U)-specific amplicons are shown for each indicated sample. The densitometry ratio of unmethylated to methylated products (U:M) is shown

below their respective amplicon pairs. SKO is included as an unmethylated control. DNAs used in this analysis were derived from the same samples as in (**C**).

(**E** and **F**) PCA plots of RNA-seq data and ERRBS data of TKO (**E**) or DKOcr298 (**F**) cells treated with DMSO, 500 nM ZEN, 100 nM dAZA, or combination ZEN + dAZA daily for 72 hrs. The single agent ZEN treated samples are overlapping the DMSO vehicle control samples in the ERRBS PCA plot (*n* = 3 for all treated samples).

(**G** and **H**) Violin plots showing global DNA methylation β values for TKO (**G**) or DKOcr298 (**H**) treated with DMSO, 500 nM ZEN, 100 nM dAZA, or combination ZEN + dAZA daily for 72 hrs. The median is indicated by a solid line in each plot.

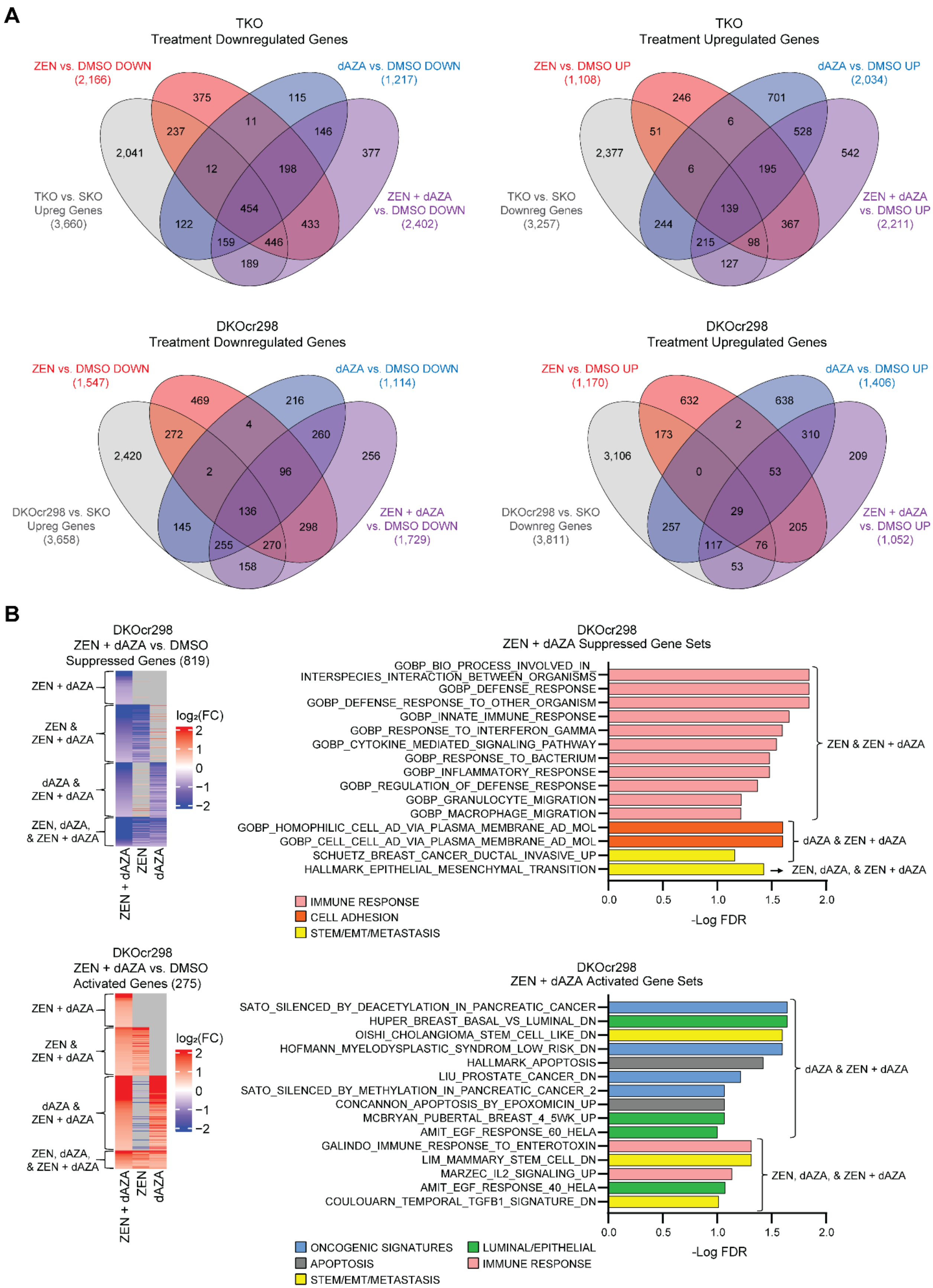
A
TKO
Treatment Downregulated Genes
ZEN vs. DMSO DOWN
(2,166)
dAZA vs. DMSO DOWN
(1,217)
375
115
237
11
146
2,041
377
12
198
TKO vs. SKO
Upreg Genes
(3,660)
454
ZEN + dAZA
vs. DMSO DOWN
(2,402)
122
433
159
446
189
TKO
Treatment Upregulated Genes
ZEN vs. DMSO UP
(1,108)
dAZA vs. DMSO UP
(2,034)
246
701
51
6
528
2,377
542
6
195
TKO vs. SKO
Downreg Genes
(3,257)
139
ZEN + dAZA
vs. DMSO UP
(2,211)
244
367
215
98
127
DKOcr298
Treatment Downregulated Genes
ZEN vs. DMSO DOWN
(1,547)
dAZA vs. DMSO DOWN
(1,114)
469
216
272
4
260
2,420
256
2
96
DKOcr298 vs. SKO
Upreg Genes
(3,658)
136
ZEN + dAZA
vs. DMSO DOWN
(1,729)
145
298
255
270
158
DKOcr298
Treatment Upregulated Genes
ZEN vs. DMSO UP
(1,170)
dAZA vs. DMSO UP
(1,406)
632
638
173
2
310
3,106
209
0
53
DKOcr298 vs. SKO
Downreg Genes
(3,811)
29
ZEN + dAZA
vs. DMSO UP
(1,052)
257
205
117
76
53
B
DKOcr298
ZEN + dAZA vs. DMSO
Suppressed Genes (819)
ZEN + dAZA
ZEN &
ZEN + dAZA
dAZA &
ZEN + dAZA
ZEN, dAZA,
& ZEN + dAZA
log₂(FC)
2
1
0
−1
−2
ZEN + dAZA
ZEN
dAZA
DKOcr298
ZEN + dAZA Suppressed Gene Sets
GOBP_BIO_PROCESS_INVOLVED_IN_
INTERSPECIES_INTERACTION_BETWEEN_ORGANISMS
GOBP_DEFENSE_RESPONSE
GOBP_DEFENSE_RESPONSE_TO_OTHER_ORGANISM
GOBP_INNATE_IMMUNE_RESPONSE
GOBP_RESPONSE_TO_INTERFERON_GAMMA
GOBP_CYTOKINE_MEDIATED_SIGNALING_PATHWAY
GOBP_RESPONSE_TO_BACTERIUM
GOBP_INFLAMMATORY_RESPONSE
GOBP_REGULATION_OF_DEFENSE_RESPONSE
GOBP_GRANULOCYTE_MIGRATION
GOBP_MACROPHAGE_MIGRATION
GOBP_HOMOPHILIC_CELL_AD_VIA_PLASMA_MEMBRANE_AD_MOL
GOBP_CELL_CELL_AD_VIA_PLASMA_MEMBRANE_AD_MOL
SCHUETZ_BREAST_CANCER_DUCTAL_INVASIVE_UP
HALLMARK_EPITHELIAL_MESENCHYMAL_TRANSITION
ZEN & ZEN + dAZA
dAZA & ZEN + dAZA
ZEN, dAZA, & ZEN + dAZA
0.0
0.5
1.0
1.5
2.0
-Log FDR
IMMUNE RESPONSE
CELL ADHESION
STEM/EMT/METASTASIS
DKOcr298
ZEN + dAZA vs. DMSO
Activated Genes (275)
ZEN + dAZA
ZEN &
ZEN + dAZA
dAZA &
ZEN + dAZA
ZEN, dAZA,
& ZEN + dAZA
log₂(FC)
2
1
0
−1
−2
ZEN + dAZA
ZEN
dAZA
DKOcr298
ZEN + dAZA Activated Gene Sets
SATO_SILENCED_BY_DEACETYLATION_IN_PANCREATIC_CANCER
HUPER_BREAST_BASAL_VS_LUMINAL_DN
OISHI_CHOLANGIOMA_STEM_CELL_LIKE_DN
HOFMANN_MYELODYSPLASTIC_SYNDROM_LOW_RISK_DN
HALLMARK_APOPTOSIS
LIU_PROSTATE_CANCER_DN
SATO_SILENCED_BY_METHYLATION_IN_PANCREATIC_CANCER_2
CONCANNON_APOPTOSIS_BY_EPOXOMICIN_UP
MCBRYAN_PUBERTAL_BREAST_4_5WK_UP
AMIT_EGF_RESPONSE_60_HELA
GALINDO_IMMUNE_RESPONSE_TO_ENTEROTOXIN
LIM_MAMMARY_STEM_CELL_DN
MARZEC_IL2_SIGNALING_UP
AMIT_EGF_RESPONSE_40_HELA
COULOUARN_TEMPORAL_TGFB1_SIGNATURE_DN
dAZA & ZEN + dAZA
ZEN, dAZA, & ZEN + dAZA
0.0
0.5
1.0
1.5
2.0
-Log FDR
ONCOGENIC SIGNATURES
APOPTOSIS
STEM/EMT/METASTASIS
LUMINAL/EPITHELIAL
IMMUNE RESPONSE

Supplemental Figure 9. Lineage plasticity genes reversed with ZEN + dAZA combination treatment in TKO and DKOcr298

(**A**) Venn diagrams comparing (left) genes significantly upregulated (fold change > 1.5; *P*adj < 0.05) with *Trp53*/*Rb1* loss in TKO or DKOcr298 cells vs. genes significantly downregulated (fold change < -1.5; *P*adj < 0.05) in ZEN single agent treated, dAZA single agent treated, or ZEN + dAZA combination treated TKO or DKOcr298 cells and (right) genes significantly downregulated (fold change < -1.5; *P*adj < 0.05) with *Trp53*/*Rb1* loss in TKO or DKOcr298 cells vs. genes significantly upregulated (fold change > 1.5; *P*adj < 0.05) in ZEN single agent treated, dAZA single agent treated, or ZEN + dAZA combination treated TKO or DKOcr298 cells.

(**B**) Representative top gene sets (FDR < 0.1) comprised of *Trp53*/*Rb1* loss-modulated genes that are reversed with ZEN + dAZA combination treatment in DKOcr298. (Left) Heatmaps depicting the relative differential gene expression in single agent or combination treated vs. vehicle treated control samples for *Trp53*/*Rb1* loss-modulated genes that are reversed with treatment. (Right) Gene set ORA on genes modulated by *Trp53*/*Rb1* loss-induced lineage plasticity that were reversed by combination treatment only (ZEN + dAZA), ZEN alone or combination treatment (ZEN & ZEN + dAZA), dAZA alone or combination treatment (dAZA & ZEN + dAZA), or either single agent alone or combination treatment (ZEN, dAZA, & ZEN + dAZA). Statistical significance was determined using hypergeometric test followed by Benjamini-Hochberg correction.

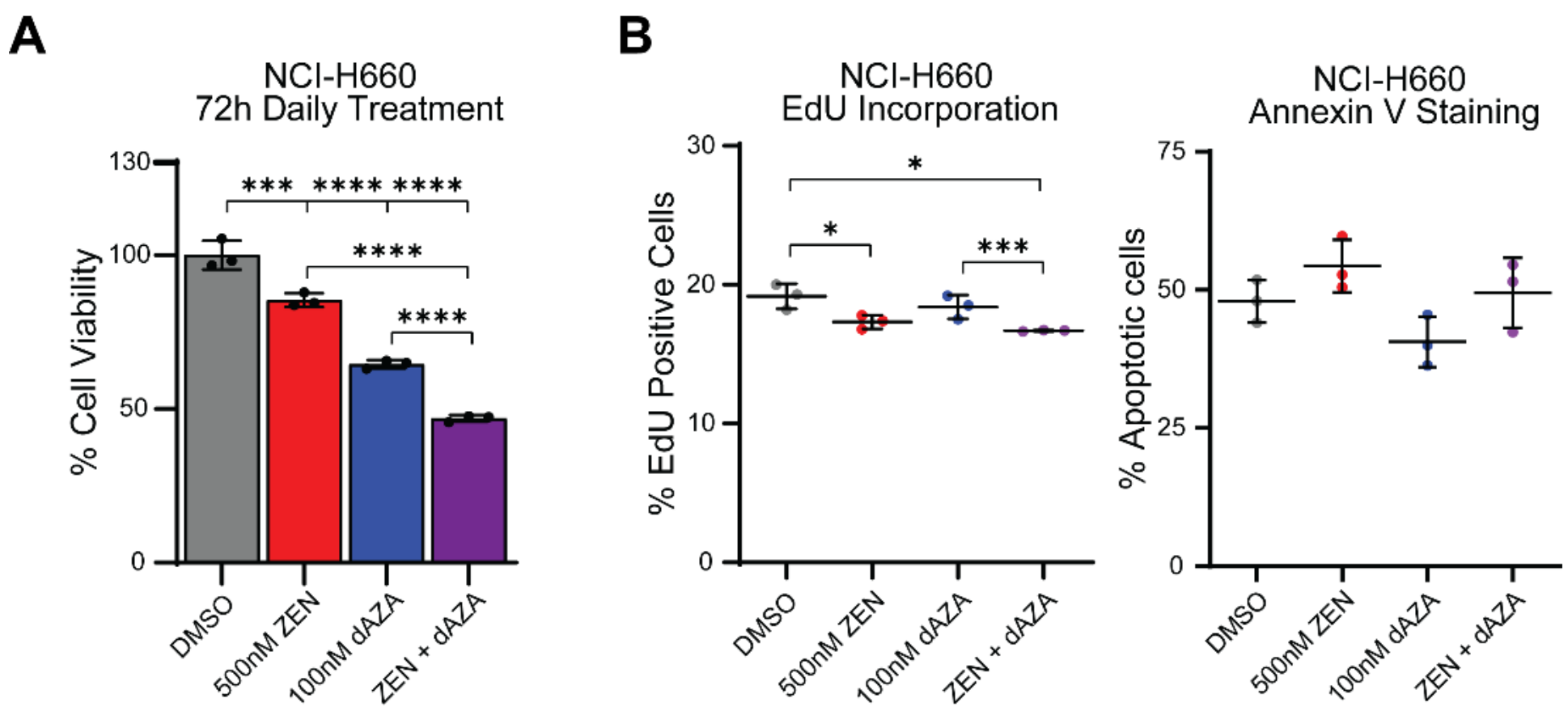

Supplemental Figure 10. ZEN + dAZA combination treatment in NCI-H660.

(**A**) Cell count viability of NCI-H660 (2) cells after 72-hr daily treatment of indicated dose of ZEN alone, dAZA alone, or ZEN + dAZA combination. Data presented are the mean ± SD (n = 3 for all treated samples). Statistical significance was determined using one-way ANOVA with Benjamini-Hochberg multiple testing correction. ***$P < 0.001$; ****$P < 0.0001$.

(**B**) EdU incorporation and apoptosis assays using NCI-H660 (2) cells after 72-hr daily treatment with indicated dose of ZEN alone, dAZA alone, or ZEN + dAZA combination. Data presented are the mean ± SD (n = 3 for all treated samples). Statistical significance was determined using one-way ANOVA with Benjamini-Hochberg multiple testing correction. *$P < 0.05$; ***$P < 0.001$.

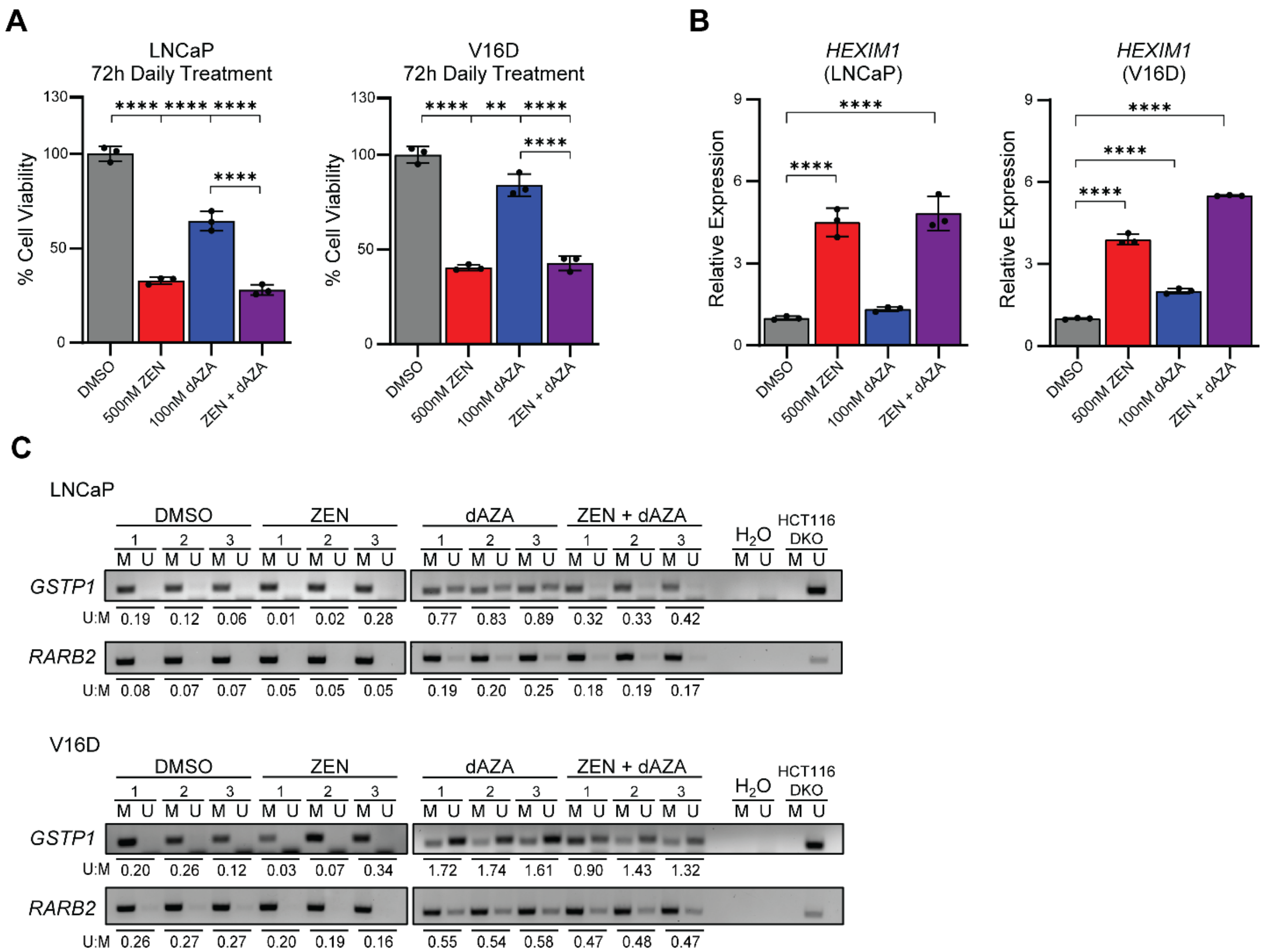

Supplemental Figure 11. ZEN + dAZA combination treatment in *TP53*/*RB1*-intact adenocarcinoma cell lines.

(**A**) Cell count viability of LNCaP or V16D (3) cells after 72-hr daily treatment with indicated dose of ZEN alone, dAZA alone, or ZEN + dAZA combination. Data presented are the mean ± SD (*n* = 3 for all treated samples). Statistical significance was determined using one-way ANOVA with Benjamini-Hochberg multiple testing correction. ***P* < 0.01; *****P* < 0.0001.

(**B**) RNA-qPCR of BETi-activated gene *HEXIM1* (1) in LNCaP or V16D (3) treated with indicated dose of ZEN alone, dAZA alone, or ZEN + dAZA combination daily for 72 hrs. Data presented are

the mean ± SD (n = 3 for all treated samples). Statistical significance was determined using one-way ANOVA with Dunnett's multiple-comparison test. ****$P < 0.0001$.

(**C**) MSPCR analysis of the hypermethylated genes *GSTP1* (4) and *RARB2* (5) in LNCaP or V16D (3) treated with indicated dose of ZEN alone, dAZA alone, or ZEN + dAZA combination daily for 72 hrs. Methylated (M) and unmethylated (U) specific amplicons are shown for each indicated sample. The densitometry ratio of unmethylated to methylated products (U:M) is shown below their respective amplicon pairs. HCT116 DKO (6) is included as an unmethylated control.

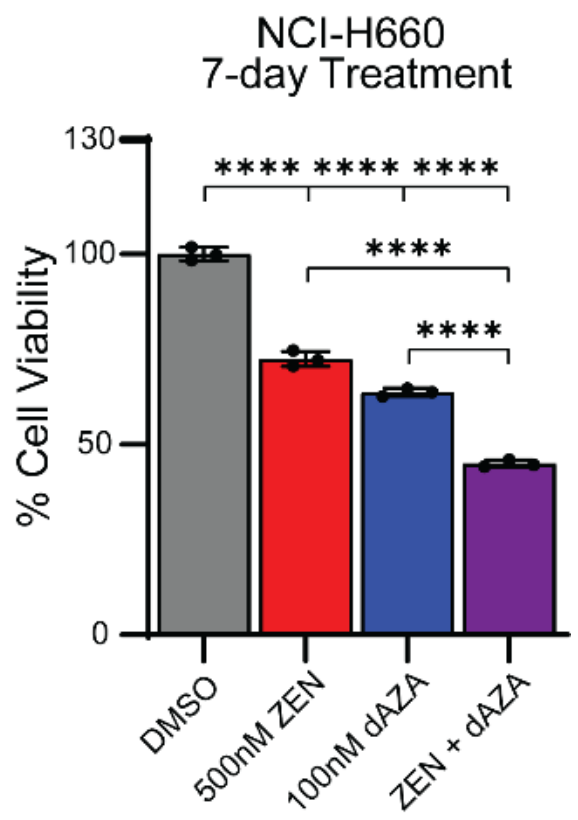

Supplemental Figure 12. Long term ZEN + dAZA combination treatment in NCI-H660.

Cell count viability of NCI-H660 (2) cells after long term treatments. Cells were treated with indicated doses of ZEN, dAZA, or the combination for 72 hrs daily before plating followed by sustained ZEN or ZEN-vehicle treatment without dAZA for 7 days. Data presented are the mean ± SD (n = 3 for all treated samples). Statistical significance was determined using one-way ANOVA with Benjamini-Hochberg multiple testing correction. ****P < 0.0001.

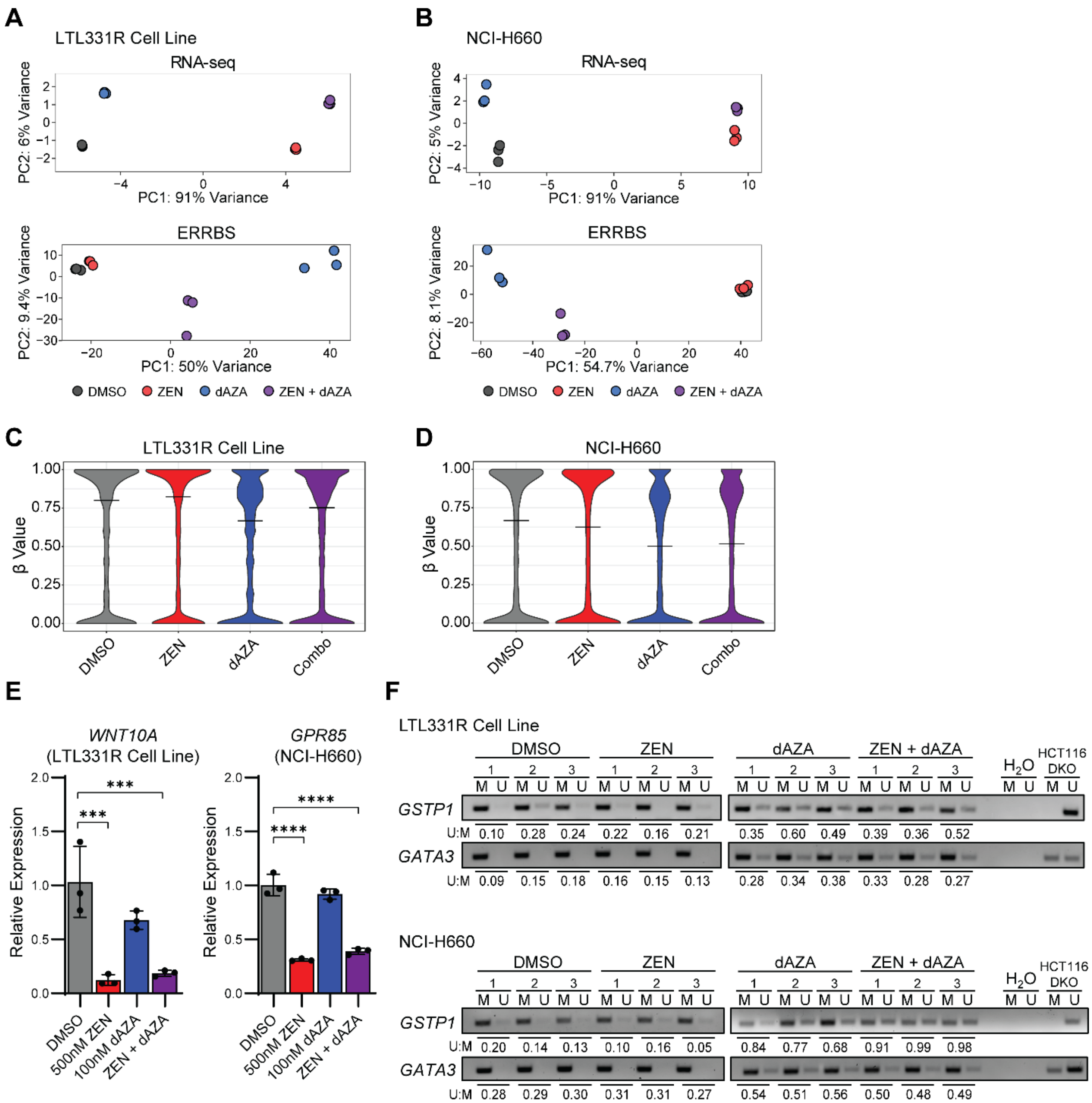

Supplemental Figure 13. Gene expression and DNA methylation changes from ZEN + dAZA combination treatment in LTL331R and NCI-H660 cell lines.

(**A** and **B**) PCA plots of RNA-seq data and ERRBS data of LTL331R (7) (**A**) or NCI-H660 (2) (**B**) cells treated with DMSO, 500 nM ZEN, 100 nM dAZA, or combination ZEN + dAZA daily for 72

hrs. The single agent ZEN treated NCI-H660 (2) samples are overlapping the DMSO vehicle control NCI-H660 samples in the ERRBS PCA plot (*n* = 3 for all treated samples).

(**C** and **D**) Violin plots showing global DNA methylation β values for LTL331R (7) (**C**) or NCI-H660 (2) (**D**) cells treated with DMSO, 500 nM ZEN, 100 nM dAZA, or combination ZEN + dAZA daily for 72 hrs. The median is indicated by a solid line in each plot.

(**E**) (Left) RNA-qPCR of BETi-suppressed genes in LTL331R (7) or NCI-H660 (2) cells treated with indicated dose of ZEN alone, dAZA alone, or ZEN + dAZA combination daily for 72 hrs. Data presented are the mean ± SD (*n* = 3 for all treated samples). Statistical significance was determined using one-way ANOVA with Dunnett's multiple-comparison test. ***$P < 0.001$; ****$P < 0.0001$.

(**F**) MSPCR analysis of hypermethylated genes in LTL331R (7) or NCI-H660 (2) cells treated with indicated dose of ZEN alone, dAZA alone, or ZEN + dAZA combination daily for 72 hrs. Methylated (M) and unmethylated (U) specific amplicons are shown for each indicated sample. The densitometry ratio of unmethylated to methylated products (U:M) is shown below their respective amplicon pairs. HCT116 DKO (6) is included as an unmethylated control.

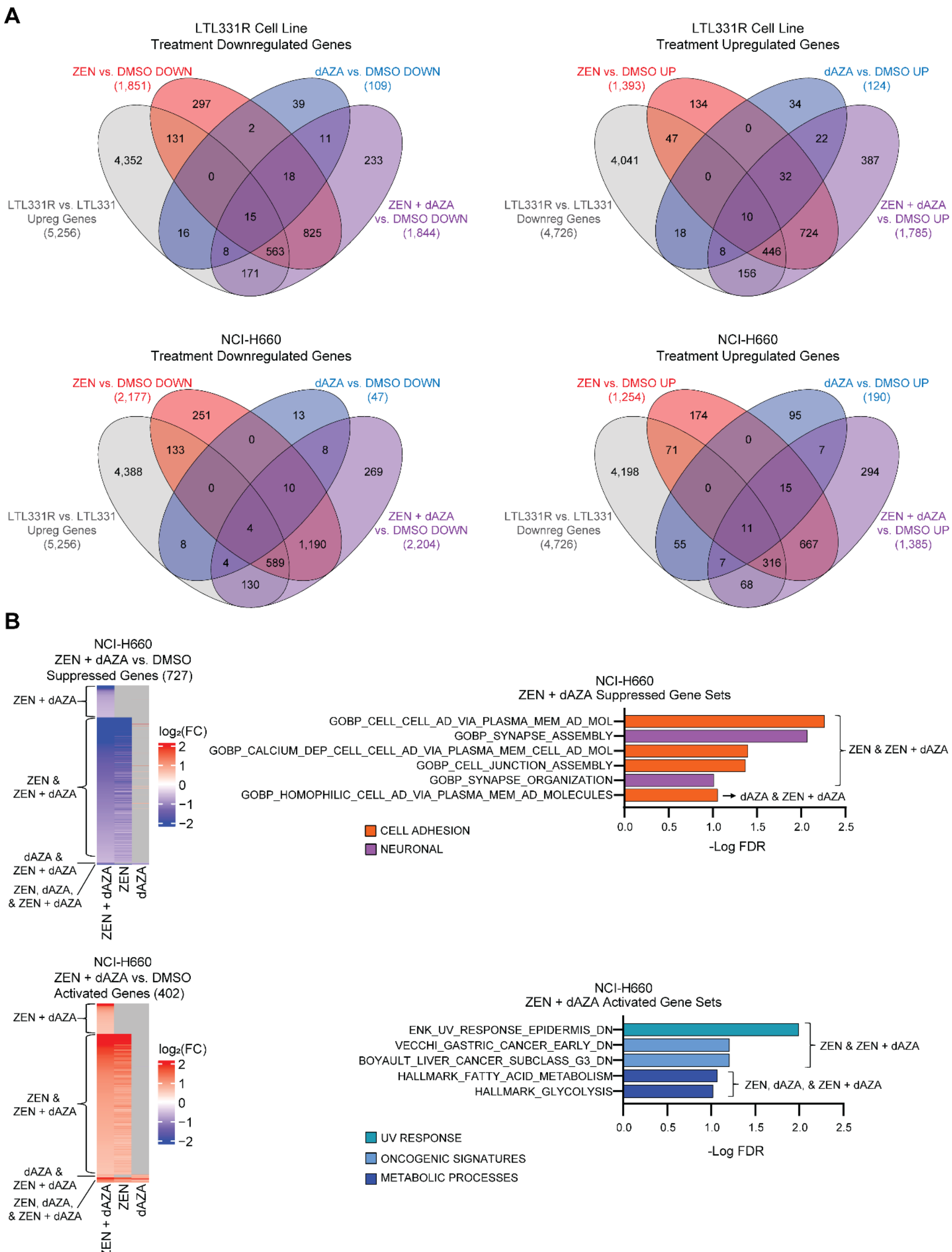
A
LTL331R Cell Line
Treatment Downregulated Genes
ZEN vs. DMSO DOWN (1,851)
dAZA vs. DMSO DOWN (109)
LTL331R vs. LTL331 Upreg Genes (5,256)
ZEN + dAZA vs. DMSO DOWN (1,844)
297
39
131
2
11
4,352
0
18
233
15
16
825
8
563
171
LTL331R Cell Line
Treatment Upregulated Genes
ZEN vs. DMSO UP (1,393)
dAZA vs. DMSO UP (124)
LTL331R vs. LTL331 Downreg Genes (4,726)
ZEN + dAZA vs. DMSO UP (1,785)
134
34
47
0
22
4,041
0
32
387
10
18
724
8
446
156
NCI-H660
Treatment Downregulated Genes
ZEN vs. DMSO DOWN (2,177)
dAZA vs. DMSO DOWN (47)
LTL331R vs. LTL331 Upreg Genes (5,256)
ZEN + dAZA vs. DMSO DOWN (2,204)
251
13
133
0
8
4,388
0
10
269
4
8
1,190
4
589
130
NCI-H660
Treatment Upregulated Genes
ZEN vs. DMSO UP (1,254)
dAZA vs. DMSO UP (190)
LTL331R vs. LTL331 Downreg Genes (4,726)
ZEN + dAZA vs. DMSO UP (1,385)
174
95
71
0
7
4,198
0
15
294
11
55
667
7
316
68
B
NCI-H660
ZEN + dAZA vs. DMSO
Suppressed Genes (727)
ZEN + dAZA
ZEN & ZEN + dAZA
dAZA & ZEN + dAZA
ZEN, dAZA, & ZEN + dAZA
ZEN + dAZA
ZEN
dAZA
log₂(FC)
2
1
0
−1
−2
NCI-H660
ZEN + dAZA Suppressed Gene Sets
GOBP_CELL_CELL_AD_VIA_PLASMA_MEM_AD_MOL
GOBP_SYNAPSE_ASSEMBLY
GOBP_CALCIUM_DEP_CELL_CELL_AD_VIA_PLASMA_MEM_CELL_AD_MOL
GOBP_CELL_JUNCTION_ASSEMBLY
GOBP_SYNAPSE_ORGANIZATION
GOBP_HOMOPHILIC_CELL_AD_VIA_PLASMA_MEM_AD_MOLECULES
ZEN & ZEN + dAZA
dAZA & ZEN + dAZA
0.0
0.5
1.0
1.5
2.0
2.5
-Log FDR
CELL ADHESION
NEURONAL
NCI-H660
ZEN + dAZA vs. DMSO
Activated Genes (402)
ZEN + dAZA
ZEN & ZEN + dAZA
dAZA & ZEN + dAZA
ZEN, dAZA, & ZEN + dAZA
ZEN + dAZA
ZEN
dAZA
log₂(FC)
2
1
0
−1
−2
NCI-H660
ZEN + dAZA Activated Gene Sets
ENK_UV_RESPONSE_EPIDERMIS_DN
VECCHI_GASTRIC_CANCER_EARLY_DN
BOYAULT_LIVER_CANCER_SUBCLASS_G3_DN
HALLMARK_FATTY_ACID_METABOLISM
HALLMARK_GLYCOLYSIS
ZEN & ZEN + dAZA
ZEN, dAZA, & ZEN + dAZA
0.0
0.5
1.0
1.5
2.0
2.5
-Log FDR
UV RESPONSE
ONCOGENIC SIGNATURES
METABOLIC PROCESSES

Supplemental Figure 14. Lineage plasticity genes reversed with ZEN + dAZA combination treatment in LTL331R and NCI-H660 cell lines.

(**A**) Venn diagrams comparing (left) genes significantly upregulated (fold change > 1.5; *P*adj < 0.05) with castration-induced lineage plasticity (LTL331R vs. LTL331 (7)) vs. genes significantly downregulated (fold change < -1.5; *P*adj < 0.05) in ZEN single agent treated, dAZA single agent treated, or ZEN + dAZA combination treated LTL331R (7) or NCI-H660 (2) cells and (right) genes significantly downregulated (fold change < -1.5; *P*adj < 0.05) with castration-induced lineage plasticity (LTL331R vs. LTL331 (7)) vs. genes significantly upregulated (fold change > 1.5; *P*adj < 0.05) in ZEN single agent treated, dAZA single agent treated, or ZEN + dAZA combination treated LTL331R (7) or NCI-H660 (2) cells.

(**B**) Representative top gene sets (FDR < 0.1) comprised of castration-induced lineage plasticity modulated genes that are reversed with ZEN + dAZA combination treatment in NCI-H660 (2) cells. (Left) Heatmaps depicting the relative differential gene expression in single agent or combination treated vs. vehicle treated control samples for castration-induced lineage plasticity modulated genes that are reversed with treatment. (Right) Gene set ORA on genes modulated by castration-induced lineage plasticity that were reversed by combination treatment only (ZEN + dAZA), ZEN alone or combination treatment (ZEN & ZEN + dAZA), dAZA alone or combination treatment (dAZA & ZEN + dAZA), or either single agent alone or combination treatment (ZEN, dAZA, & ZEN + dAZA). Statistical significance was determined using hypergeometric test followed by Benjamini-Hochberg correction.

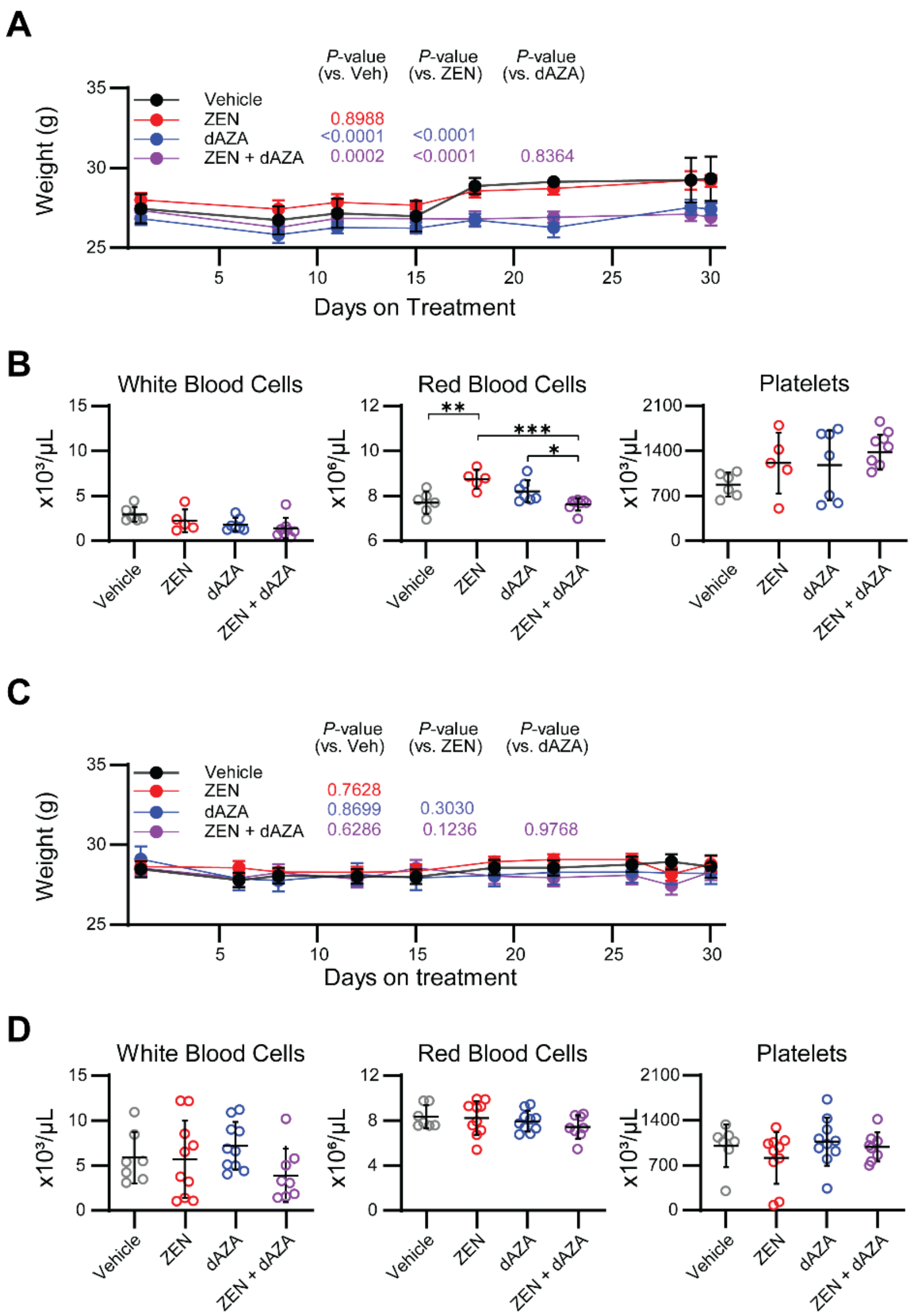

Supplemental Figure 15. ZEN + dAZA combination treatment is well tolerated in vivo.

(**A**) Body weight measurements for LTL331R (7) PDX-bearing mice. Data shown are the mean ± SD (Vehicle, *n* = 7; ZEN, *n* = 6; dAZA, *n* = 7; ZEN + dAZA, *n* = 8). Statistical significance was determined using two-way ANOVA followed by Tukey's multiple comparison test.

(**B**) Blood cell counts from time of LTL331R (7) tumor harvest. Data shown are the mean ± SD (Vehicle, *n* = 6; ZEN, *n* = 5; dAZA, *n* = 7; ZEN + dAZA, *n* = 8). Statistical significance was

determined using one-way ANOVA with Benjamini-Hochberg multiple testing correction. *$P < 0.05$; **$P < 0.01$; ***$P < 0.001$.

(**C**) Body weight measurements for TKO tumor-bearing mice for the duration of the study. Data shown are the mean ± SD (Vehicle, $n = 11$; ZEN, $n = 11$; dAZA, $n = 10$; ZEN + dAZA, $n = 11$). Statistical significance was determined using two-way ANOVA followed by Tukey's multiple comparison test.

(**D**) Blood cell counts from time of TKO tumor harvest. Data shown are the mean ± SD (Vehicle $n = 7$; ZEN $n = 10$; dAZA $n = 10$; ZEN + dAZA $n = 8$). Statistical significance was determined using one-way ANOVA with Benjamini-Hochberg multiple testing correction.

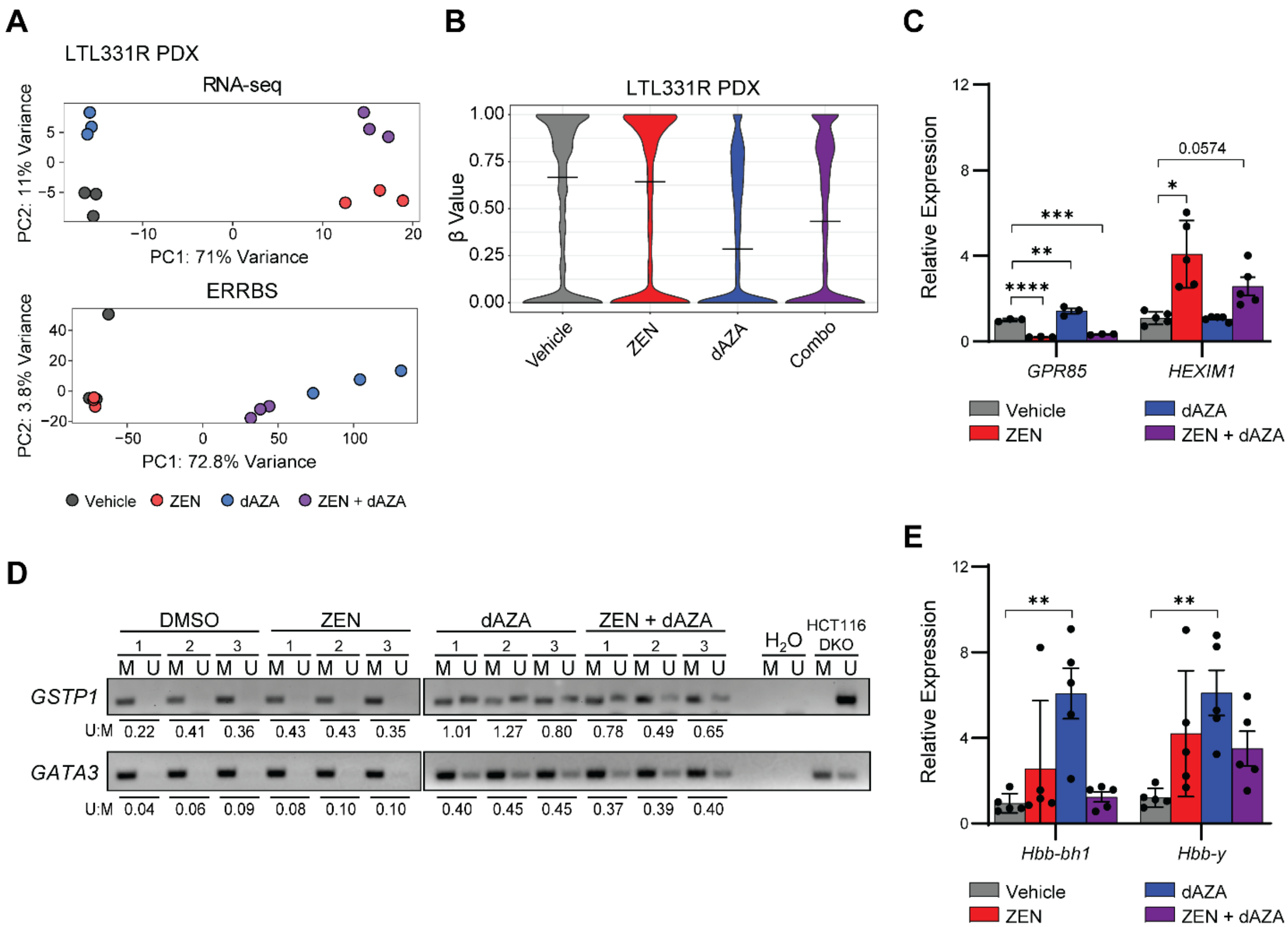

Supplemental Figure 16. ZEN + dAZA combination treatment target engagement in LTL331R PDX.

(**A**) PCA plots of RNA-seq data and ERRBS data of LTL331R (7) PDX treated with vehicle, ZEN, dAZA, or combination ZEN + dAZA. The single agent ZEN treated samples are overlapping the vehicle control samples in the ERRBS PCA plot (*n* = 3 for all treated samples).

(**B**) Violin plots showing global DNA methylation β values for LTL331R (7) PDX treated with vehicle, ZEN, dAZA, or combination ZEN + dAZA. The median is indicated by a solid line.

(**C**) RNA-qPCR of BETi-suppressed genes in LTL331R (7) PDXs treated with ZEN alone, dAZA alone, or ZEN + dAZA combination. Data presented are the mean ± SD (*n* = 5 for each treatment

condition). Statistical significance was determined using one-way ANOVA with Dunnett's multiple-comparison test. ***$P < 0.001$; ****$P < 0.0001$.

(**D**) MSPCR analysis of hypermethylated genes in LTL331R (7) PDX treated with ZEN alone, dAZA alone, or ZEN + dAZA combination. Methylated (M) and unmethylated (U) specific amplicons are shown for each indicated sample. The densitometry ratio of unmethylated to methylated products (U:M) is shown below their respective amplicon pairs. HCT116 DKO (6) is included as an unmethylated control.

(**E**) RNA-qPCR of fetal globin genes in bone marrow from ZEN, dAZA, or ZEN + dAZA combination treated mice at study end point. Data presented are the mean ± SD ($n = 5$ for each treatment condition). Statistical significance was determined using one-way ANOVA with Dunnett's multiple-comparison test. **$P < 0.01$.

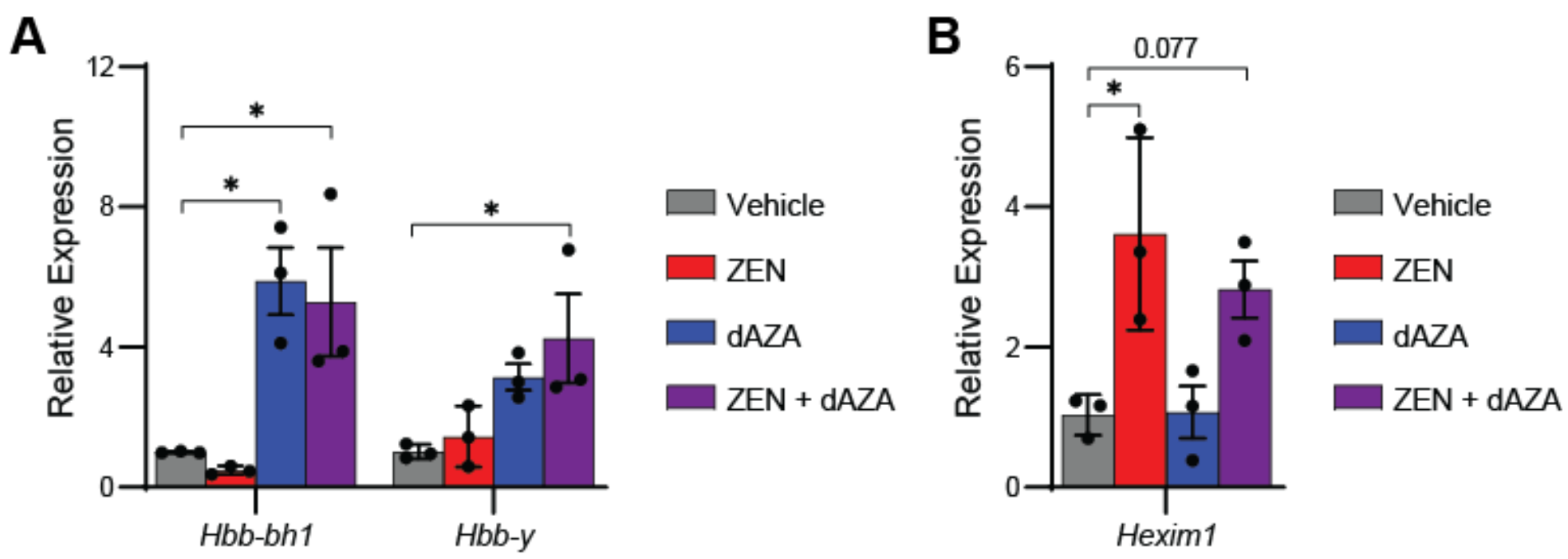

Supplemental Figure 17. ZEN + dAZA combination treatment target engagement in TKO tumor-bearing mice.

(**A**) RNA-qPCR of fetal globin genes in bone marrow from ZEN, dAZA, or ZEN + dAZA combination treated mice five days after treatment start. Data presented are the mean ± SD (n = 3 for all treated samples). Statistical significance was determined using one-way ANOVA with Dunnett's multiple-comparison test. $^*P < 0.05$.

(**B**) *Hexim1* (1) RNA-qPCR in TKO tumors harvested five days after treatment start. Data presented are the mean ± SD (n = 3 for all treated samples). Statistical significance was determined using one-way ANOVA with Dunnett's multiple-comparison test. $^*P < 0.05$.

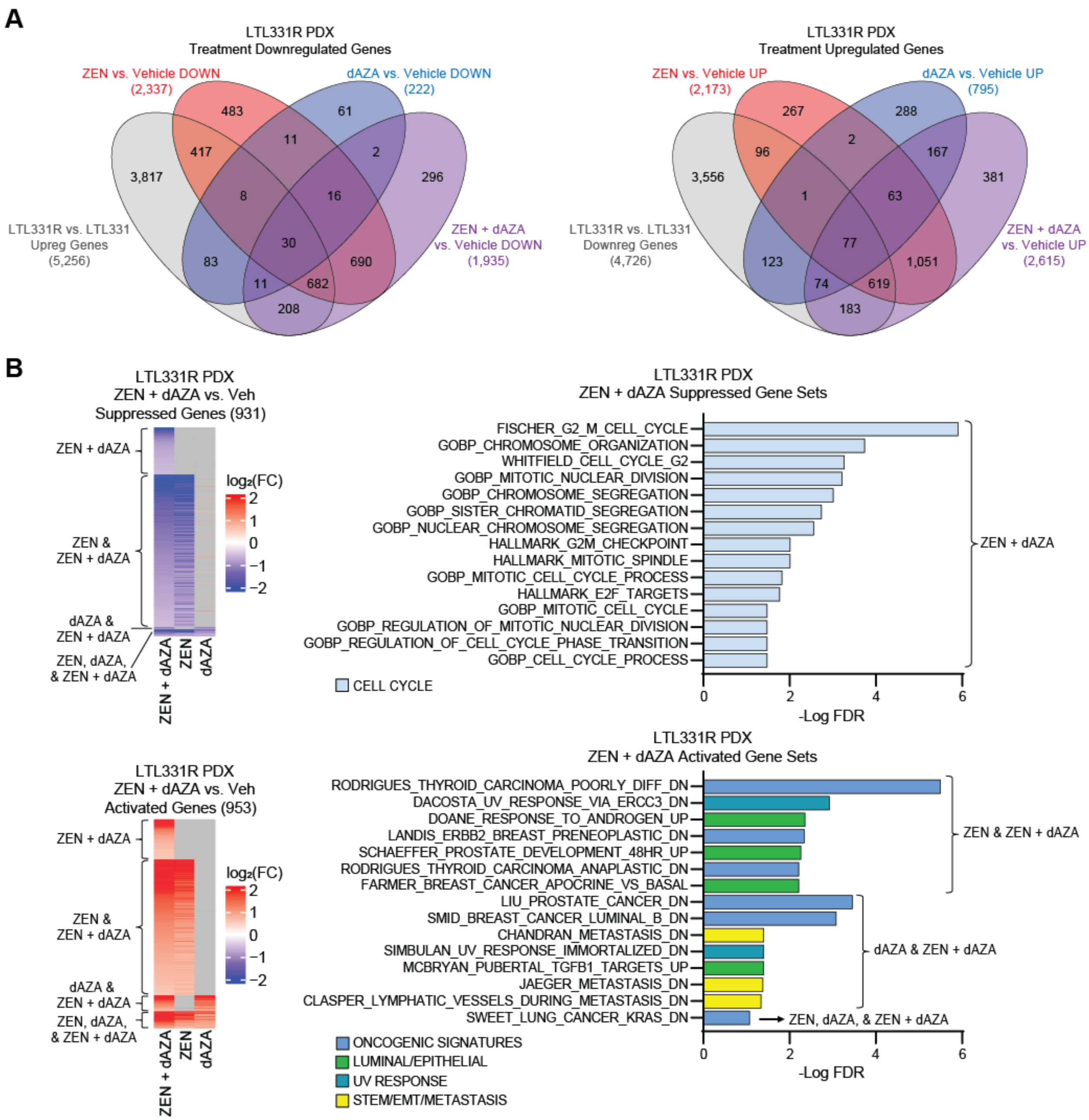

Supplemental Figure 18. Lineage plasticity genes reverse with ZEN + dAZA combination treatment in LTL331R PDX.

(**A**) Venn diagrams comparing (left) genes significantly upregulated (fold change > 1.5; *P*adj < 0.05) with castration-induced lineage plasticity (LTL331R vs. LTL331 (7)) vs. genes significantly downregulated (fold change < -1.5; *P*adj < 0.05) in ZEN single agent treated, dAZA single agent

treated, or ZEN + dAZA combination treated LTL331R (7) PDXs and (right) genes significantly downregulated (fold change < -1.5; *P*adj < 0.05) with castration-induced lineage plasticity (LTL331R vs. LTL331 (7)) vs. genes significantly upregulated (fold change > 1.5; *P*adj < 0.05) in ZEN single agent treated, dAZA single agent treated, or ZEN + dAZA combination treated LTL331R (7) PDXs.

(**B**) (Left) Heatmaps depicting the relative differential gene expression in single agent or combination treated vs. vehicle treated control tumors for castration-induced lineage plasticity modulated genes reversed with treatment. (Right) Gene set ORA (FDR < 0.1) on genes modulated by castration-induced lineage plasticity reversed by combination treatment only (ZEN + dAZA), ZEN alone or combination treatment (ZEN & ZEN + dAZA), dAZA alone or combination treatment (dAZA & ZEN + dAZA), or either single agent alone or combination treatment (ZEN, dAZA, & ZEN + dAZA). Statistical significance was determined using hypergeometric test followed by Benjamini-Hochberg correction.

Supplemental Methods

ATAC-sequencing

ATAC-seq libraries were prepared from whole cell suspensions from three biological replicates per cell line by the University of Michigan Epigenomics Core as described previously (8). ATAC-seq libraries were washed using AMPure XP SPRI beads (Beckman Coulter A63881) and Qiagen MinElute columns (Qiagen 28004), and final concentrations were measured using a Qubit HS dsDNA kit (Invitrogen Q33231). Library quality assessment was performed using a TapeStation HS D1000 kit (Screentape: Agilent 5067-5584; Reagents: Agilent 5067-5585). Final libraries were pooled and quantified using a KAPA Illumina Library quantification kit (KAPA Biosystems KK4824) followed by paired-end 150 bp sequencing using a NovaSeq 6000 platform (Illumina). Overall quality of each sequencing sample was assessed using FastQC version 0.11.8. Adaptors were removed from sequencing reads using TrimGalore version 0.4.5 and cutadapt version 1.15 with the following parameters: –nextera -e 0.1 –stringency 6 –length 20 –nextseq 20. Trimmed reads were aligned to GRCm38 using Bowtie2 version 2.3.4.1 (9) with the following parameters: -X 2000 –no-mixed –no-discordant, and default multi-seed length of 20 bp with 0 mismatches. Duplicate reads were marked using Picard version 2.20.2. Duplicate reads and low-quality alignments were removed using samtools version 1.2 (10) with the following parameters: -q 10 -F 1024. Reads that overlapped blacklisted regions (ENCODE Blacklist Regions) were removed with bedtools version 2.28.0 (11). Sample-wise peaks were called using macs2 version 2.1.2 (12). Peaks over all samples were merged using bedops version 2.4.36 (13) for principal component analysis. Regions of differential chromatin accessibility were identified using the edgeR R Bioconductor package version 3.28.1 (14) and annotated to promoter or enhancer regions with annotatr R version 1.12.1 (15).

RNA Preparation, RNA-qPCR, and RNA-seq

Total RNA was extracted from three biological replicates per cell line/condition tested unless stated otherwise using Qiagen AllPrep RNA/DNA mini kits (Qiagen 80204). To generate cDNA for RT-qPCR, 1 µg of RNA was reverse-transcribed using a High-Capacity cDNA Reverse Transcription kit (Life Technologies 4368814). 10 µL singleplex RNA-qPCR reactions contained 1x Taqman Universal PCR Master Mix (Applied Biosystems 4304437), 1x Taqman probe (Supplemental Table 18), and 10 ng RNA-equivalent cDNA. RNA-qPCR reactions were performed using a Quantstudio 3 or a Quantstudio 12K Flex thermocycler (Applied Biosystems) with the following parameters: 50˚C for 2 mins; 95˚C for 10 mins (denaturation); and 40 cycles of 95˚C for 15 sec (disassociation) and 60˚C for 1 min (annealing/extension/read). Data were analyzed using Design and Analysis Software version 1.5.2 (Life Technologies) using *GAPDH* as an endogenous control.

Library preparation and sequencing were performed as previously described (16) using three biological replicates. Libraries were barcoded, pooled, and sequenced with 150 bp paired-end cycles using a 300 cycle S4 flow cell on a NovaSeq 6000 platform (Illumina) or 150 bp paired-end cycles using a 300 cycle 10B flow cell on a NovaSeq X platform (Illumina). Adaptors were trimmed using Cutadapt version 2.3. Quality of each sequencing sample was assessed with FastQC version 0.11.8. Reads were aligned to GRCm38 using STAR version 2.7.8a (17) and read count estimates were performed using RSEM version 1.3.3 (18). Alignment options followed ENCODE standards for RNA-seq (17). Differential gene expression analyses were performed using DESeq2 version 1.36.0 (19). Annotation data from ENSEMBL 102 were used and genes were annotated with Entrez Gene IDs and text descriptions. Gene set enrichment analysis (GSEA) version 4.3.2 (20) was performed as previously described (21). Differential gene expression Venn diagrams were generated using ggvenn version 0.1.10. Heatmaps with differentially expressed genes across treatments were plotted using the ComplexHeatmap package version 2.20.0.

ERRBS

Genomic DNA from treated samples (*n* = 3) was extracted using Qiagen AllPrep RNA/DNA mini kits (Qiagen 80204). ERRBS library preparation was performed as previously described (22). Briefly, 25-75 ng of genomic DNA was digested with the MspI restriction enzyme for 16-18 hours and then purified by phenol-chloroform extraction followed by ethanol precipitation with glycogen and sodium acetate. The resulting DNA was resuspended in 10 mM Tris-Cl, pH 8.5 and processed for end repair, A-tailing, and ligation of hairpin adapters with methylated cytosines. Ligated DNA was run on a 1.8% agarose gel in TBE buffer, and fragments 100-200 bp long and 200-300 bp long were separately purified using a Qiagen Gel Extraction kit (Qiagen 28704). Proper fragment size and adaptor ligation were verified by PCR. Libraries were bisulfite converted using the EZ DNA Methylation kit (Zymo D5001) with the following changes: samples were incubated with the following thermocycler parameters: 55 cycles – 95˚C for 30 secs, 50˚C for 15 min; hold at 4˚C. The bisulfite-converted libraries were cleaned up according to the manufacturer's instructions, eluted in 40 µL of nuclease-free water, and then amplified using Roche High Fidelity FastStart (Roche 3553400001) with the following parameters: 94˚C for 5 mins (denaturation); 18 cycles – 94˚C for 20 sec (disassociation), 60˚C for 30 sec (annealing), 72˚C for 60 sec (extension); 72˚C for 60 sec (final extension); hold at 4˚C. Final library cleanup was performed using AMPure XP SPRI beads (Beckman Coulter A63881) using the following protocol: beads were brought to room temperature with gentle agitation for 30 mins. A 1.2x volume (for short fragments) and a 1.1x volume (for long fragments) of SPRI beads were added to each library. PCR products were purified according to the manufacturer's instructions using 500 µL of 80% ethanol for washes. Libraries were eluted in 33 µL of 10 mM Tris-Cl, pH 8.5 and quantified using a Qubit HS dsDNA kit (Invitrogen Q33231). Library quality was assessed using a TapeStation HS D1000 kit (Screentape: Agilent 5067-5584; Reagents: Agilent 5067-5585). Libraries were sequenced with 100 cycle sequencing on an SP flow cell using a NovaSeq 6000 platform (Illumina) or 300 cycle sequencing on a 10B flow cell using a NovaSeq X platform (Illumina). The overall quality of each

sequenced sample was assessed using FastQC version 0.11.8. Adaptors were removed from sequencing reads using TrimGalore version 0.4.5 with the following parameters: –adapter AGATCGGAAGAGC -e 0.1 –stringency 6 –length 20 –nextseq 20 –rrbs. Sequencing reads were aligned to GRCm38 with Bismark version 0.22.1 (23) and Bowtie2 version 2.3.4.1 (9) using default settings (multi-seed length of 20 bp with no mismatches). Alignments below a MAPQ threshold were removed with samtools version 1.2 (10) with the following parameters: -q 10. Methylation rates were called using MethylDackel version 0.4.0 with the parameters: -d 5 -D 2000 -mergeContext. Differential methylation analysis was performed using the methylSig R version 0.99.3 (24): CpGs with read coverage > 500 or < 10 were removed, and differential methylation was tested using the diff_methylsig() function. Differentially methylated CpGs were defined with a difference in β-value > 10 and FDR < 0.05. In order to generate violin plots showing global β values, ERRBS bedGraph files were processed using were processed using reshape2 version 1.4.4, ggplot2 version 3.5.1, and dplyr version 1.1.2. CpG-level β-values for each sample were calculated after removal of zero-coverage sites using the formula, β = methylated reads / total reads. CpG-wise mean β-values from triplicates were computed for each treatment group. Kernel density estimates of the full distribution of CpG-wise mean β-values in violin plots were generated using ggplot2 version 3.5.1.

Chromatin immunoprecipitation

ChIP experiments were performed as previously described (25) in duplicate using anti-H3K27Ac (Active Motif 39133) or rabbit IgG (Millipore 12-370). Briefly, cell suspensions were crosslinked using formaldehyde. Crosslinked cells were lysed, and the resulting chromatin was sheared using a Bioruptor Pico (Diagenode). 4 µg of antibody was added to immunoprecipitate chromatin from 4 million cells per ChIP sample and incubated overnight at 4˚C. The DNA-protein-antibody complexes were isolated and washed using 30 µL of resuspended protein A/G magnetic beads (Thermo Scientific 26162). Samples, including input chromatin, were then incubated at 65˚C

overnight to de-crosslink, and then treated with RNaseA (Thermo Scientific EN0531) at 37˚C for 1 hr followed by treatment with Proteinase K (Invitrogen AM2546) at 55˚C for 2 hrs. Captured DNA was purified using Qiagen MinElute columns (Qiagen 28004). ChIP sequencing libraries were prepped by the University of Michigan Epigenomic Core. Both input and immunoprecipitated DNA samples were quantified using a Qubit HS dsDNA kit (Invitrogen Q33231) and DNA fragment size was assessed using a TapeStation HS D1000 kit (Screentape: Agilent 5067-5584; Reagents: Agilent 5067-5585). Sequencing libraries were prepared with 10 ng DNA using a Swift Accel-NGS 2S Plus DNA kit (Swift Biosciences 21024) and a S2 Combinatorial Dual Indexing kit (Swift Biosciences 48096) according to the manufacturer's instructions. Final library amplification was performed using 6 cycles of PCR, and quality control of the libraries was performed using a Qplotubit HS dsDNA kit (Invitrogen Q33231) and a TapeStation HS D1000 kit (Screentape: Agilent 5067-5584; Reagents: Agilent 5067-5585). The libraries were quantified using a KAPA Illumina Library quantification kit (KAPA Biosystems KK4824) before paired-end 50 bp sequencing using an S1 flow cell on a NovaSeq 6000 platform (Illumina). Overall quality of each sequencing sample was assessed using FastQC version 0.11.8. Adaptors were trimmed using TrimGalore version 0.4.5 and cutadapt version 1.15. Trimmed sequencing reads were aligned to GRCm38 using Bowtie2 version 2.4.1 (9) using default parameters with the exception of the following flags: -X 2000. Alignments were filtered with samtools version 1.2 (10). Sample-wise peaks were called using macs2 version 2.1.2 (12) with flags: –format BAMPE –gsize mm. Peaks over all samples were merged using bedops version 2.4.36 (13) for principal component analysis and unsupervised clustering to assess the similarity of the samples.

Author contributions

WKS, DF, AK, ZD, JAY, and JJA designed the research. WKS, DF, AK, ZD, SC, CZ, ER, JAK, and JAY performed experiments. WKS, DF, AK, ZD, SC, CZ, ER, DK, OAS, JAK, CRA, HNB, and JAY acquired data. RM, SM, and AU performed IHC evaluations. DF, KB, RGC, CC, FZ, YMH, ZX,

RJR, and MC performed computational analyses. WKS, DF, AK, ZD, and JJA analyzed the data. PSN, LE, and YW provided cell line or PDX models. WKS, DF, and JJA wrote the manuscript. WKS, DF, AK, ZD, SC, ER, AC, JAK, PSN, CM, MCH, LE, JAY, and JJA revised the manuscript. The order of the co-first authors was determined based on their relative contributions to this study. All authors read and approved the final manuscript.

Supplemental References

1. Lin X, Huang X, Uziel T, Hessler P, Albert DH, Roberts-Rapp LA, et al. HEXIM1 as a Robust Pharmacodynamic Marker for Monitoring Target Engagement of BET Family Bromodomain Inhibitors in Tumors and Surrogate Tissues. *Mol Cancer Ther*. 2017;16(2):388-96.
2. van Bokhoven A, Varella-Garcia M, Korch C, Johannes WU, Smith EE, Miller HL, et al. Molecular characterization of human prostate carcinoma cell lines. *Prostate*. 2003;57(3):205-25.
3. Bishop JL, Thaper D, Vahid S, Davies A, Ketola K, Kuruma H, et al. The Master Neural Transcription Factor BRN2 Is an Androgen Receptor-Suppressed Driver of Neuroendocrine Differentiation in Prostate Cancer. *Cancer Discov*. 2017;7(1):54-71.
4. Cairns P, Esteller M, Herman JG, Schoenberg M, Jeronimo C, Sanchez-Cespedes M, et al. Molecular detection of prostate cancer in urine by GSTP1 hypermethylation. *Clin Cancer Res*. 2001;7(9):2727-30.
5. Florl AR, Steinhoff C, Muller M, Seifert HH, Hader C, Engers R, et al. Coordinate hypermethylation at specific genes in prostate carcinoma precedes LINE-1 hypomethylation. *Br J Cancer*. 2004;91(5):985-94.
6. Rhee I, Bachman KE, Park BH, Jair KW, Yen RW, Schuebel KE, et al. DNMT1 and DNMT3b cooperate to silence genes in human cancer cells. *Nature*. 2002;416(6880):552-6.
7. Lin D, Wyatt AW, Xue H, Wang Y, Dong X, Haegert A, et al. High fidelity patient-derived xenografts for accelerating prostate cancer discovery and drug development. *Cancer Res*. 2014;74(4):1272-83.
8. Corces MR, Trevino AE, Hamilton EG, Greenside PG, Sinnott-Armstrong NA, Vesuna S, et al. An improved ATAC-seq protocol reduces background and enables interrogation of frozen tissues. *Nat Methods*. 2017;14(10):959-62.

9. Langmead B, and Salzberg SL. Fast gapped-read alignment with Bowtie 2. *Nat Methods*. 2012;9(4):357-9.

10. Li H, Handsaker B, Wysoker A, Fennell T, Ruan J, Homer N, et al. The Sequence Alignment/Map format and SAMtools. *Bioinformatics*. 2009;25(16):2078-9.

11. Quinlan AR, and Hall IM. BEDTools: a flexible suite of utilities for comparing genomic features. *Bioinformatics*. 2010;26(6):841-2.

12. Zhang Y, Liu T, Meyer CA, Eeckhoute J, Johnson DS, Bernstein BE, et al. Model-based analysis of ChIP-Seq (MACS). *Genome Biol*. 2008;9(9):R137.

13. Neph S, Kuehn MS, Reynolds AP, Haugen E, Thurman RE, Johnson AK, et al. BEDOPS: high-performance genomic feature operations. *Bioinformatics*. 2012;28(14):1919-20.

14. McCarthy DJ, Chen Y, and Smyth GK. Differential expression analysis of multifactor RNA-Seq experiments with respect to biological variation. *Nucleic Acids Res*. 2012;40(10):4288-97.

15. Cavalcante RG, and Sartor MA. annotatr: genomic regions in context. *Bioinformatics*. 2017;33(15):2381-3.

16. Kim DH, Sun D, Storck WK, Welker Leng K, Jenkins C, Coleman DJ, et al. BET Bromodomain Inhibition Blocks an AR-Repressed, E2F1-Activated Treatment-Emergent Neuroendocrine Prostate Cancer Lineage Plasticity Program. *Clin Cancer Res*. 2021;27(17):4923-36.

17. Dobin A, Davis CA, Schlesinger F, Drenkow J, Zaleski C, Jha S, et al. STAR: ultrafast universal RNA-seq aligner. *Bioinformatics*. 2013;29(1):15-21.

18. Li B, and Dewey CN. RSEM: accurate transcript quantification from RNA-Seq data with or without a reference genome. *BMC Bioinformatics*. 2011;12:323.

19. Love MI, Huber W, and Anders S. Moderated estimation of fold change and dispersion for RNA-seq data with DESeq2. *Genome Biol*. 2014;15(12):550.

20. Subramanian A, Tamayo P, Mootha VK, Mukherjee S, Ebert BL, Gillette MA, et al. Gene set enrichment analysis: a knowledge-based approach for interpreting genome-wide expression profiles. *Proc Natl Acad Sci U S A*. 2005;102(43):15545-50.

21. Kumaraswamy A, Duan Z, Flores D, Zhang C, Sehrawat A, Hu YM, et al. LSD1 promotes prostate cancer reprogramming by repressing TP53 signaling independently of its demethylase function. *JCI Insight*. 2023;8(15).

22. Garrett-Bakelman FE, Sheridan CK, Kacmarczyk TJ, Ishii J, Betel D, Alonso A, et al. Enhanced reduced representation bisulfite sequencing for assessment of DNA methylation at base pair resolution. *J Vis Exp*. 2015(96):e52246.

23. Krueger F, and Andrews SR. Bismark: a flexible aligner and methylation caller for Bisulfite-Seq applications. *Bioinformatics*. 2011;27(11):1571-2.

24. Park Y, Figueroa ME, Rozek LS, and Sartor MA. MethylSig: a whole genome DNA methylation analysis pipeline. *Bioinformatics*. 2014;30(17):2414-22.

25. Nelson JD, Denisenko O, and Bomsztyk K. Protocol for the fast chromatin immunoprecipitation (ChIP) method. *Nat Protoc*. 2006;1(1):179-85.

26. Choi N, Park J, Lee JS, Yoe J, Park GY, Kim E, et al. miR-93/miR-106b/miR-375-CIC-CRABP1: a novel regulatory axis in prostate cancer progression. *Oncotarget.* 2015;6(27):23533-47.

27. Ren X, Chen X, Fang K, Zhang X, Wei X, Zhang T, et al. COL5A2 Promotes Proliferation and Invasion in Prostate Cancer and Is One of Seven Gleason-Related Genes That Predict Recurrence-Free Survival. *Front Oncol*. 2021;11:583083.

28. Mou D, Wu S, Chen Y, Wang Y, Dai Y, Tang M, et al. Roles of PEG10 in cancer and neurodegenerative disorder (Review). *Oncol Rep*. 2025;53(5).

29. Romero R, Chu T, Gonzalez Robles TJ, Smith P, Xie Y, Kaur H, et al. The neuroendocrine transition in prostate cancer is dynamic and dependent on ASCL1. *Nat Cancer*. 2024;5(11):1641-59.

30. Santos NJ, Mosele FC, Barquilha CN, Barbosa IC, Lima FO, Barbosa GO, et al. SLIT/ROBO Pathway and Prostate Cancer: Gene and Protein Expression and Their Prognostic Values. *Int J Mol Sci*. 2025;26(11).

31. Cassandri M, Smirnov A, Novelli F, Pitolli C, Agostini M, Malewicz M, et al. Zinc-finger proteins in health and disease. *Cell Death Discov*. 2017;3:17071.

32. Liu YY, Tanikawa C, Ueda K, and Matsuda K. INKA2, a novel p53 target that interacts with the serine/threonine kinase PAK4. *Int J Oncol*. 2019;54(6):1907-20.

33. Singer BB, Scheffrahn I, and Obrink B. The tumor growth-inhibiting cell adhesion molecule CEACAM1 (C-CAM) is differently expressed in proliferating and quiescent epithelial cells and regulates cell proliferation. *Cancer Res*. 2000;60(5):1236-44.

34. Ke ZB, You Q, Chen JY, Sun JB, Xue YT, Zhuang RB, et al. A radiation resistance related index for biochemical recurrence and tumor immune environment in prostate cancer patients. *Comput Biol Med*. 2022;146:105711.

35. Ribarska T, Goering W, Droop J, Bastian KM, Ingenwerth M, and Schulz WA. Deregulation of an imprinted gene network in prostate cancer. *Epigenetics*. 2014;9(5):704-17.

36. Park HW, Song MS, Sim HJ, Ryu PD, and Lee SY. The role of the voltage-gated potassium channel, Kv2.1 in prostate cancer cell migration. *BMB Rep*. 2021;54(2):130-5.

37. Adamo P, and Ladomery MR. The oncogene ERG: a key factor in prostate cancer. *Oncogene*. 2016;35(4):403-14.

38. Feng J, Zhang Y, Ren X, Li D, Fu H, Liu C, et al. Leucine-rich repeat containing 4 act as an autophagy inhibitor that restores sensitivity of glioblastoma to temozolomide. *Oncogene*. 2020;39(23):4551-66.

39. Jeter CR, Liu B, Lu Y, Chao HP, Zhang D, Liu X, et al. NANOG reprograms prostate cancer cells to castration resistance via dynamically repressing and engaging the AR/FOXA1 signaling axis. *Cell Discov*. 2016;2:16041.

40. Pham TT, Angus SP, and Johnson GL. MAP3K1: Genomic Alterations in Cancer and Function in Promoting Cell Survival or Apoptosis. *Genes Cancer*. 2013;4(11-12):419-26.

41. Xie M, Xie R, Xie S, Wu Y, Wang W, Li X, et al. Thioredoxin interacting protein (TXNIP) acts as a tumor suppressor in human prostate cancer. *Cell Biol Int*. 2020;44(10):2094-106.

42. Zheng Y, Lin ZY, Xie JJ, Jiang FN, Chen CJ, Li JX, et al. ARRDC3 Inhibits the Progression of Human Prostate Cancer Through ARRDC3-ITGbeta4 Pathway. *Curr Mol Med*. 2017;17(3):221-9.

43. Choezom D, Plum JM, Karuna MP, Danieli-Mackay A, Lenz C, Brockmeyer P, et al. The Ceramide-Dependent EV Secretome Differentially Affects Prostate Cancer Cell Migration. *Cells*. 2025;14(7).

44. Kim YJ, Yoon HY, Kim SK, Kim YW, Kim EJ, Kim IY, et al. EFEMP1 as a novel DNA methylation marker for prostate cancer: array-based DNA methylation and expression profiling. *Clin Cancer Res*. 2011;17(13):4523-30.

45. Guo H, Ci X, Ahmed M, Hua JT, Soares F, Lin D, et al. ONECUT2 is a driver of neuroendocrine prostate cancer. *Nat Commun*. 2019;10(1):278.

46. Mu P, Zhang Z, Benelli M, Karthaus WR, Hoover E, Chen CC, et al. SOX2 promotes lineage plasticity and antiandrogen resistance in TP53- and RB1-deficient prostate cancer. *Science*. 2017;355(6320):84-8.

47. Storck WK, May AM, Westbrook TC, Duan Z, Morrissey C, Yates JA, et al. The Role of Epigenetic Change in Therapy-Induced Neuroendocrine Prostate Cancer Lineage Plasticity. *Front Endocrinol (Lausanne)*. 2022;13:926585.

48. Bakht MK, Hayward JJ, Shahbazi-Raz F, Skubal M, Tamura R, Stringer KF, et al. Identification of alternative protein targets of glutamate-ureido-lysine associated with PSMA tracer uptake in prostate cancer cells. *Proc Natl Acad Sci U S A*. 2022;119(4).

49. Wang H, Liang S, Du X, Zhao G, Bai Y, Li J, et al. RAB26 promotes prostate cancer progression via the MAPK/ERK-TWIST1 signaling axis. *Genes Dis*. 2025;12(6):101689.

50. Santos NJ, Barquilha CN, Barbosa IC, Macedo RT, Lima FO, Justulin LA, et al. Syndecan Family Gene and Protein Expression and Their Prognostic Values for Prostate Cancer. *Int J Mol Sci*. 2021;22(16).

51. Qiu X, Pascal LE, Song Q, Zang Y, Ai J, O'Malley KJ, et al. Physical and Functional Interactions between ELL2 and RB in the Suppression of Prostate Cancer Cell Proliferation, Migration, and Invasion. *Neoplasia*. 2017;19(3):207-15.

52. Chang SN, Lee JM, Oh H, and Park JH. Glutathione Peroxidase 3 Inhibits Prostate Tumorigenesis in TRAMP Mice. *Prostate*. 2016;76(15):1387-98.

53. Cheng CY, Zhou Z, Stone M, Lu B, Flesken-Nikitin A, Nanus DM, et al. Membrane metalloendopeptidase suppresses prostate carcinogenesis by attenuating effects of gastrin-releasing peptide on stem/progenitor cells. *Oncogenesis*. 2020;9(3):38.

54. Corella AN, Cabiliza Ordonio MVA, Coleman I, Lucas JM, Kaipainen A, Nguyen HM, et al. Identification of Therapeutic Vulnerabilities in Small-cell Neuroendocrine Prostate Cancer. *Clin Cancer Res*. 2020;26(7):1667-77.

55. Xia G, Kumar SR, Masood R, Zhu S, Reddy R, Krasnoperov V, et al. EphB4 expression and biological significance in prostate cancer. *Cancer Res*. 2005;65(11):4623-32.

56. Mao L, Yang C, Wang J, Li W, Wen R, Chen J, et al. SATB1 is overexpressed in metastatic prostate cancer and promotes prostate cancer cell growth and invasion. *J Transl Med*. 2013;11:111.

57. Proenca CC, Gao KP, Shmelkov SV, Rafii S, and Lee FS. Slitrks as emerging candidate genes involved in neuropsychiatric disorders. *Trends Neurosci*. 2011;34(3):143-53.

58. Yamagata M. Structure and Functions of Sidekicks. *Front Mol Neurosci*. 2020;13:139.

59. Garrett SH, Sens MA, Shukla D, Flores L, Somji S, Todd JH, et al. Metallothionein isoform 1 and 2 gene expression in the human prostate: downregulation of MT-1X in advanced prostate cancer. *Prostate*. 2000;43(2):125-35.

60. Wang R, Du Y, Shang J, Dang X, and Niu G. PTPN14 acts as a candidate tumor suppressor in prostate cancer and inhibits cell proliferation and invasion through modulating LATS1/YAP signaling. *Mol Cell Probes*. 2020;53:101642.
61. Audet-Walsh E, Yee T, Tam IS, and Giguere V. Inverse Regulation of DHT Synthesis Enzymes 5alpha-Reductase Types 1 and 2 by the Androgen Receptor in Prostate Cancer. *Endocrinology*. 2017;158(4):1015-21.
62. Lin W, Zhang S, Gu C, Zhu H, and Liu Y. GLIPR2: a potential biomarker and therapeutic target unveiled - Insights from extensive pan-cancer analyses, with a spotlight on lung adenocarcinoma. *Front Immunol*. 2024;15:1280525.
63. Nouruzi S, Ganguli D, Tabrizian N, Kobelev M, Sivak O, Namekawa T, et al. ASCL1 activates neuronal stem cell-like lineage programming through remodeling of the chromatin landscape in prostate cancer. *Nat Commun*. 2022;13(1):2282.
64. Duan Z, Shi M, Kumaraswamy A, Lin D, Khokhani D, Wang Y, et al. PROX1 is an early driver of lineage plasticity in prostate cancer. *J Clin Invest*. 2025;135(11).
65. Zhang J, Chen Z, Mao Y, He Y, Wu X, Wu J, et al. ID2 Promotes Lineage Transition of Prostate Cancer through FGFR and JAK-STAT Signaling. *Cancers (Basel)*. 2024;16(2).
66. Maunsell HR, Ellis K, Kelley MW, and Driver EC. Lrrn1 Regulates Medial Boundary Formation in the Developing Mouse Organ of Corti. *J Neurosci*. 2023;43(29):5305-18.
67. Wang Y, Chen J, Gao WQ, and Yang R. METTL14 promotes prostate tumorigenesis by inhibiting THBS1 via an m6A-YTHDF2-dependent mechanism. *Cell Death Discov*. 2022;8(1):143.
68. Braadland PR, Ramberg H, Grytli HH, Urbanucci A, Nielsen HK, Guldvik IJ, et al. The beta(2)-Adrenergic Receptor Is a Molecular Switch for Neuroendocrine Transdifferentiation of Prostate Cancer Cells. *Mol Cancer Res*. 2019;17(11):2154-68.

69. Wei Q, Costanzi S, Liu QZ, Gao ZG, and Jacobson KA. Activation of the P2Y1 receptor induces apoptosis and inhibits proliferation of prostate cancer cells. *Biochem Pharmacol*. 2011;82(4):418-25.
