# Supplementary material for: Combined BET bromodomain and DNA methyltransferase inhibition targets critical survival pathways in transdifferentiated prostate cancer
Source: JCI Insight. 2026 Aug 11;11(18):e207543. doi: 10.1172/jci.insight.207543 (PMC13596728; doi:10.1172/jci.insight.207543)

Full unedited gel for Figure 3E

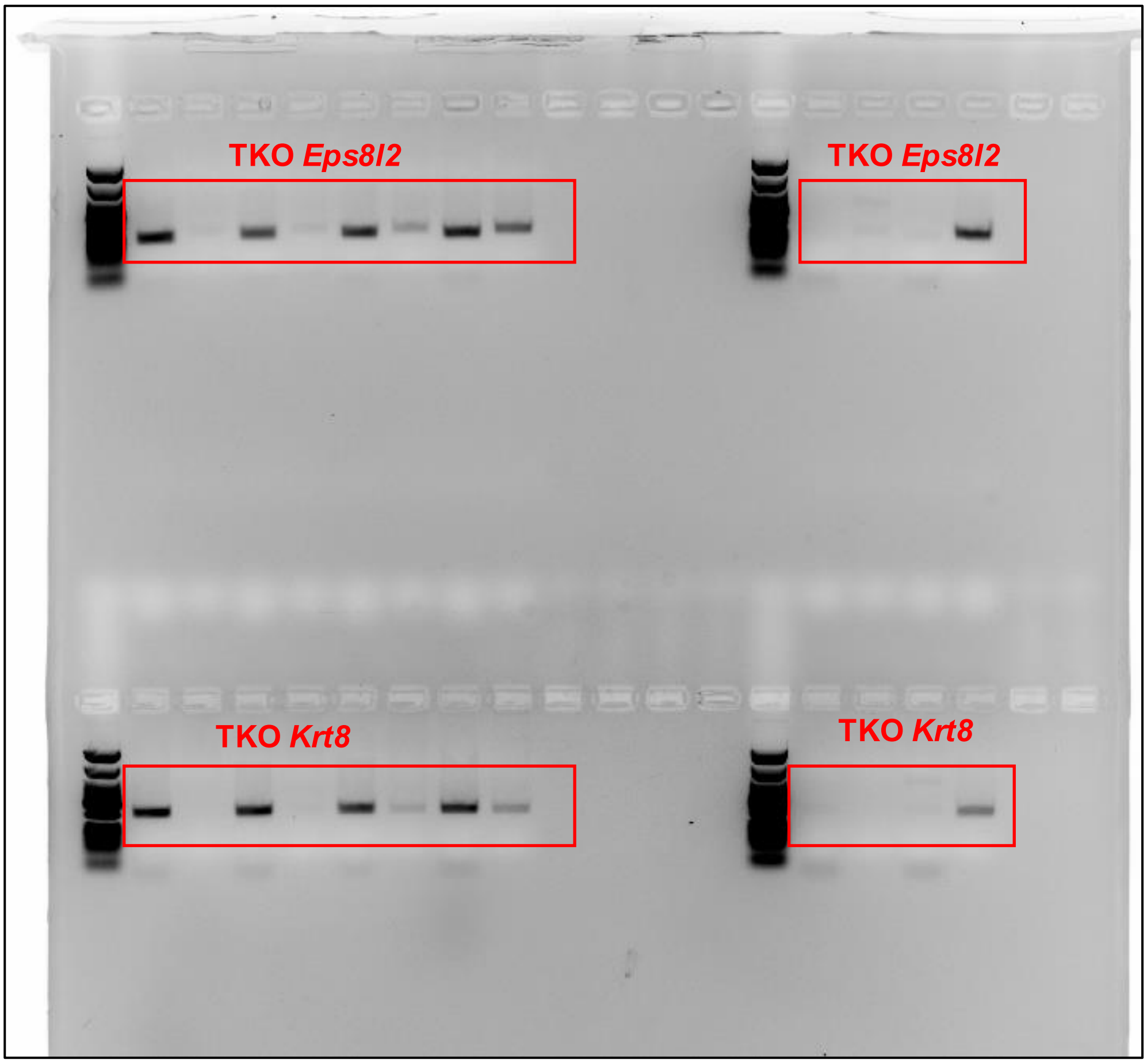

Full unedited gel for S.Figure 3G

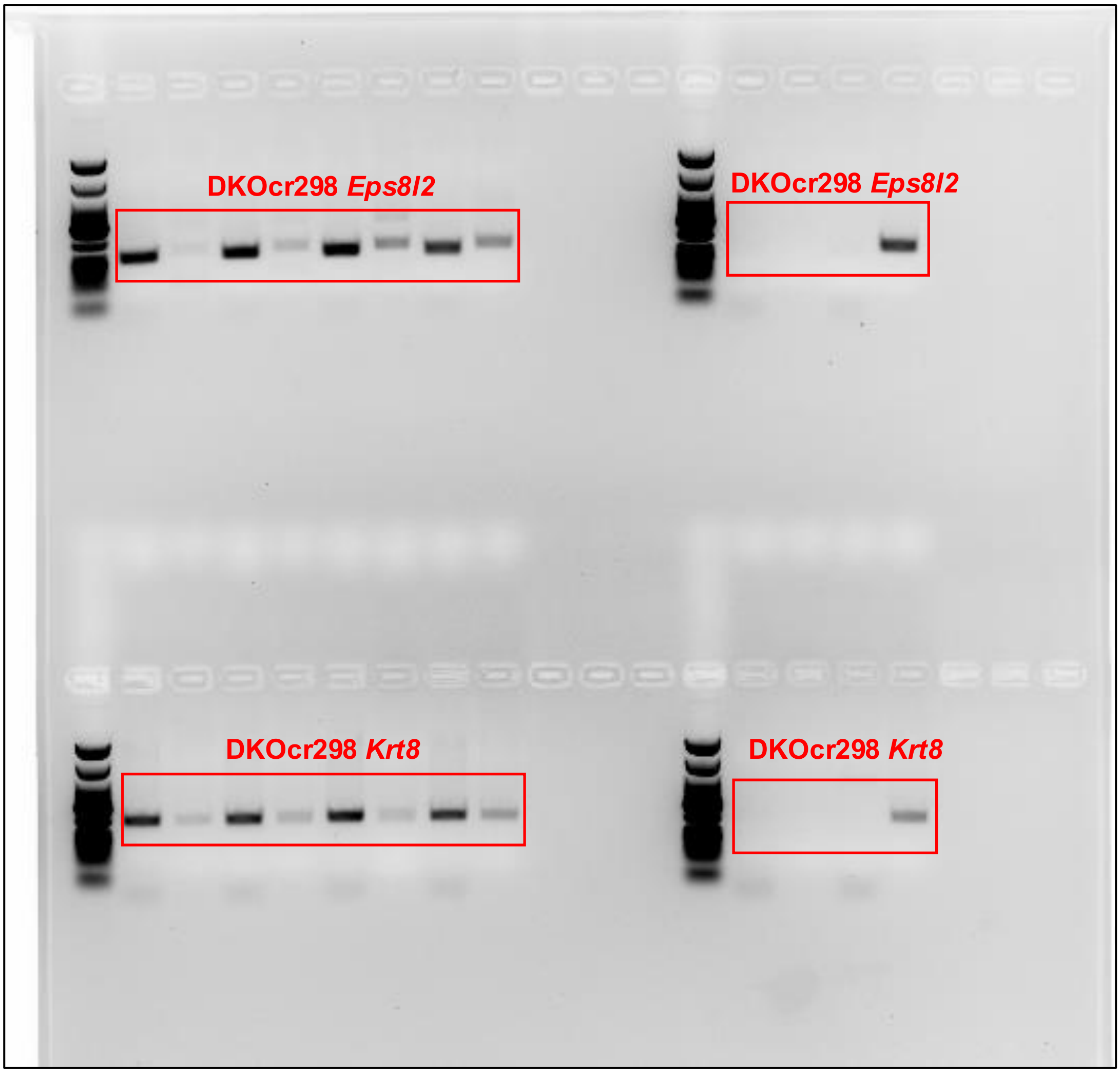

Full unedited gel for S.Figure 5C

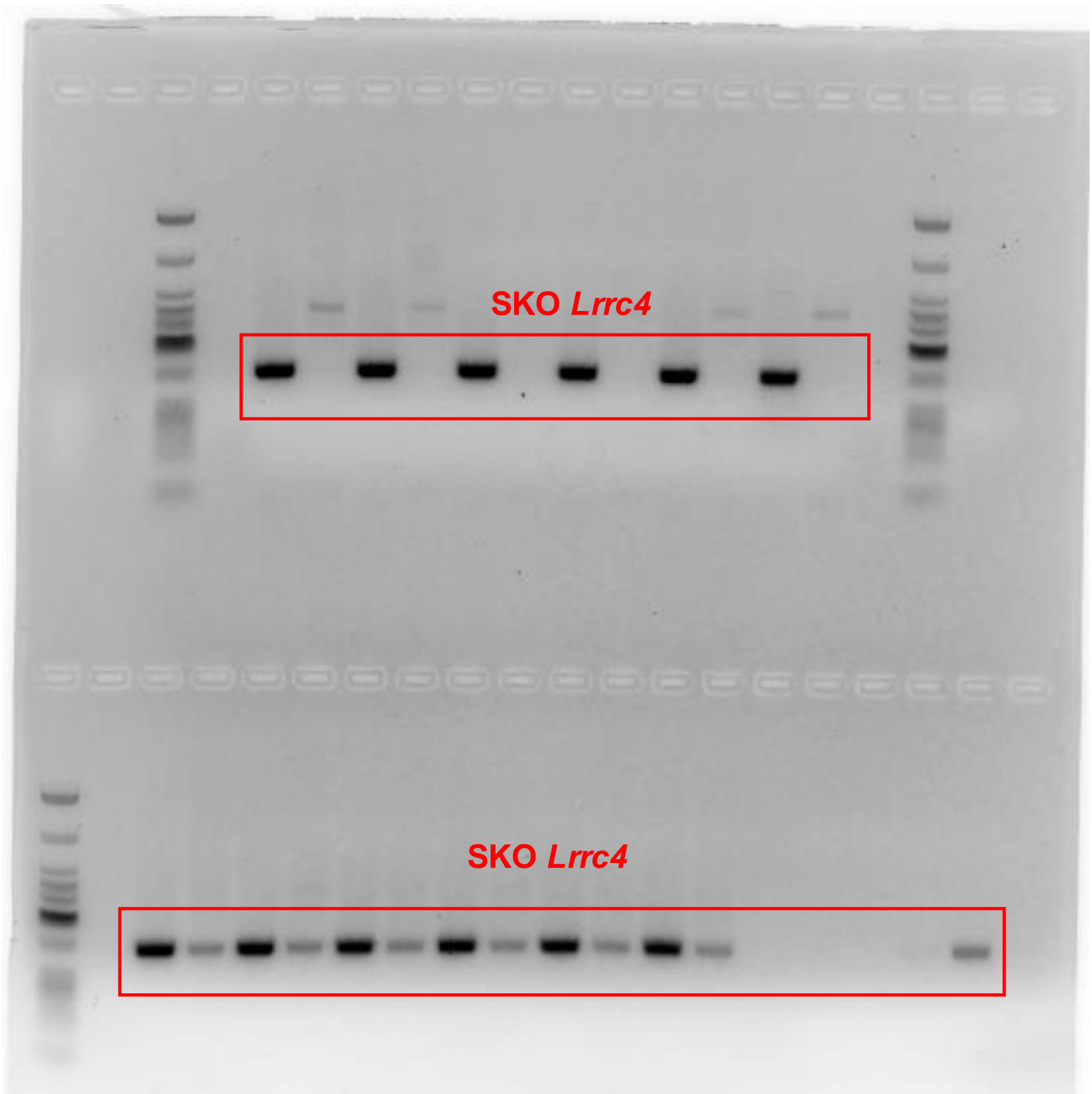

Full unedited gel for S.Figure 8B

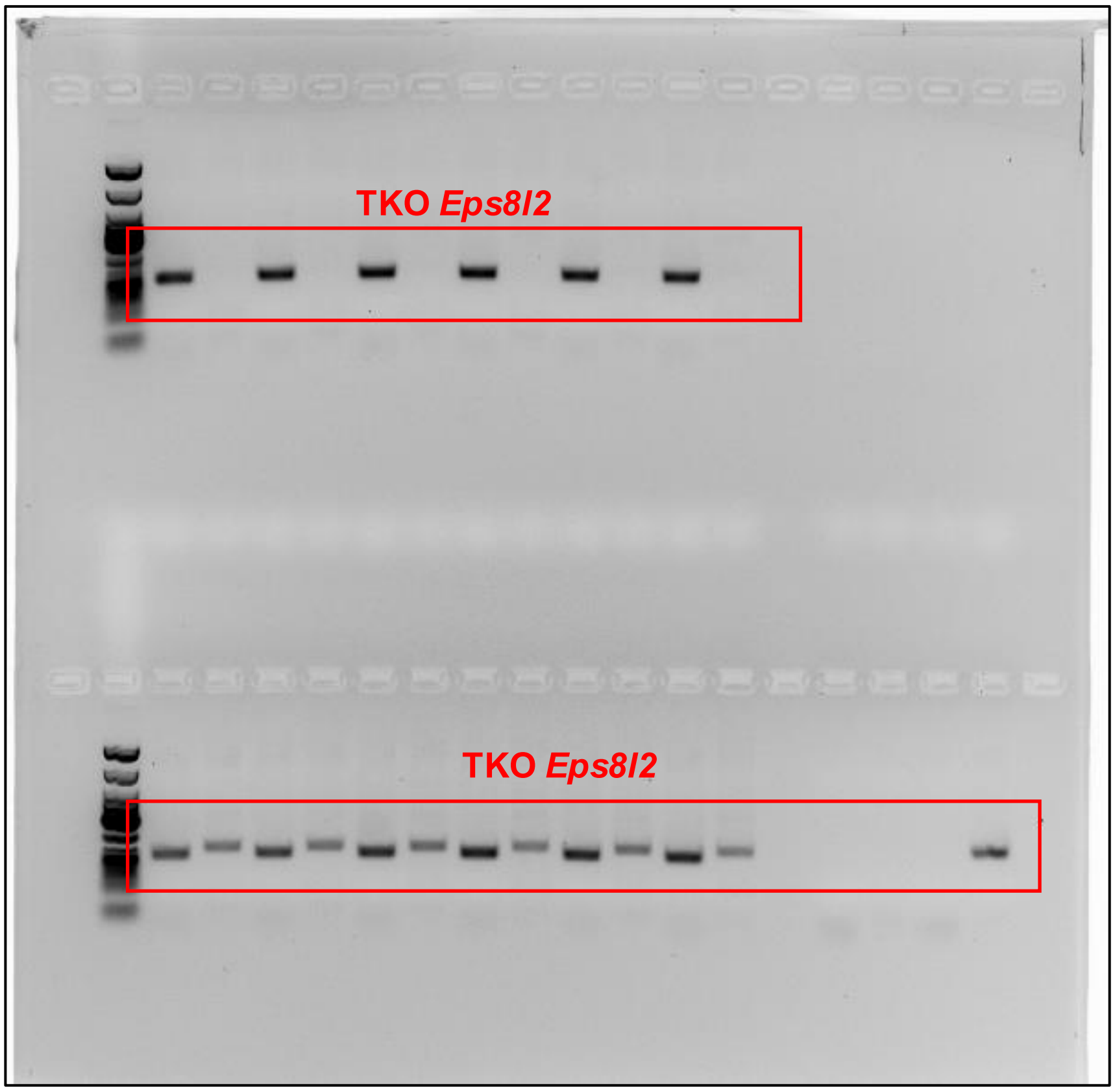

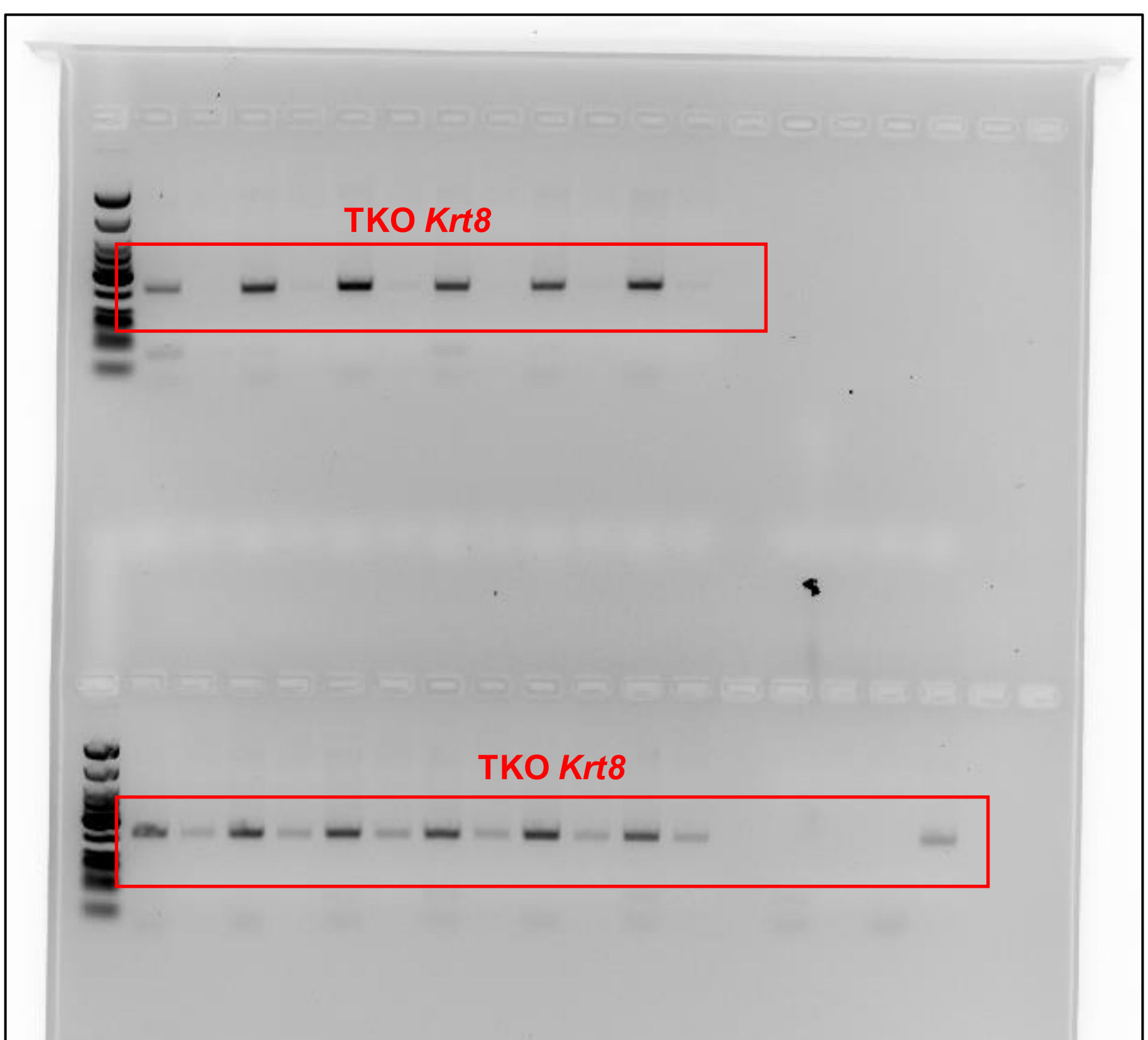

Full unedited gel for S.Figure 8D

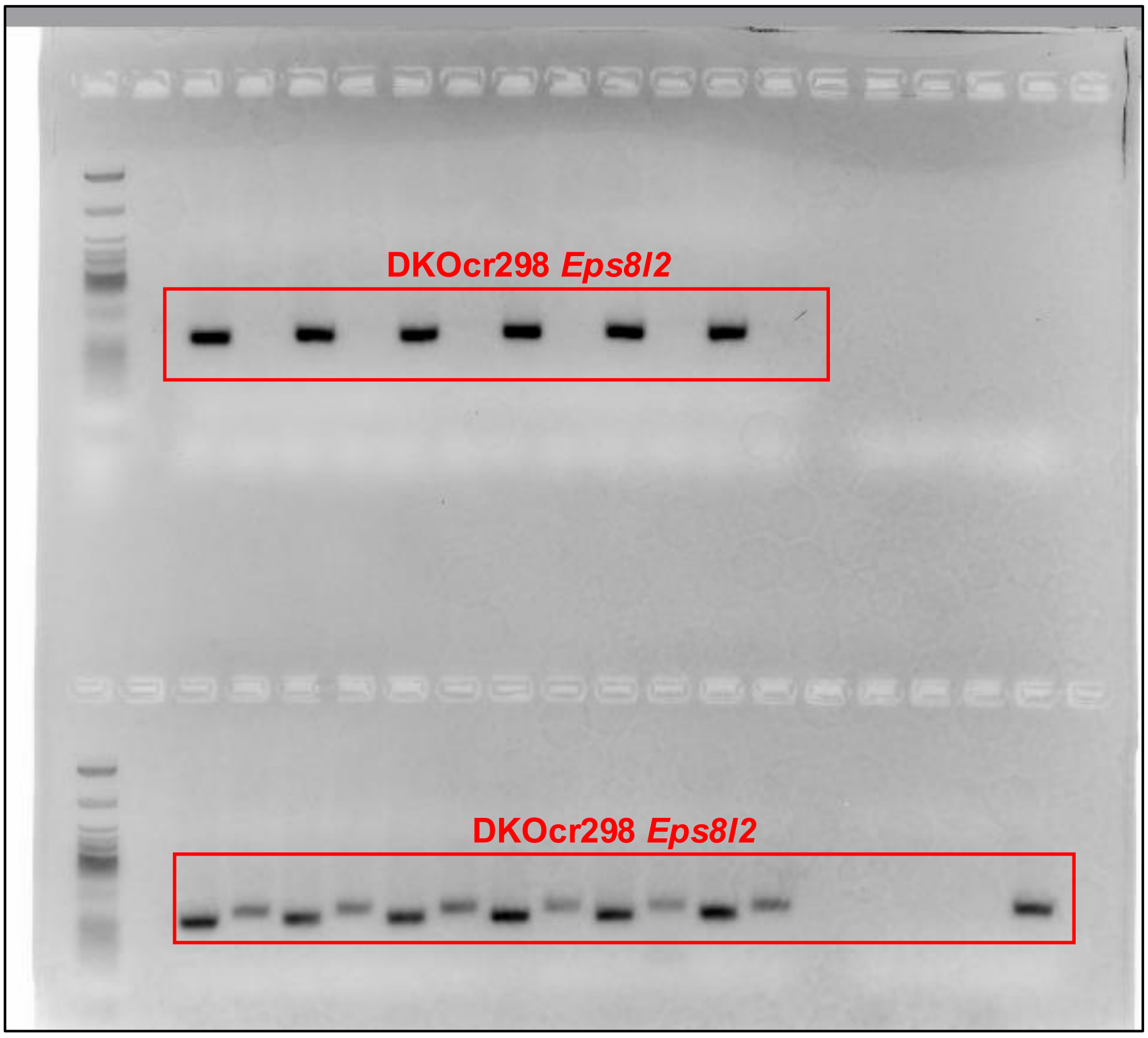

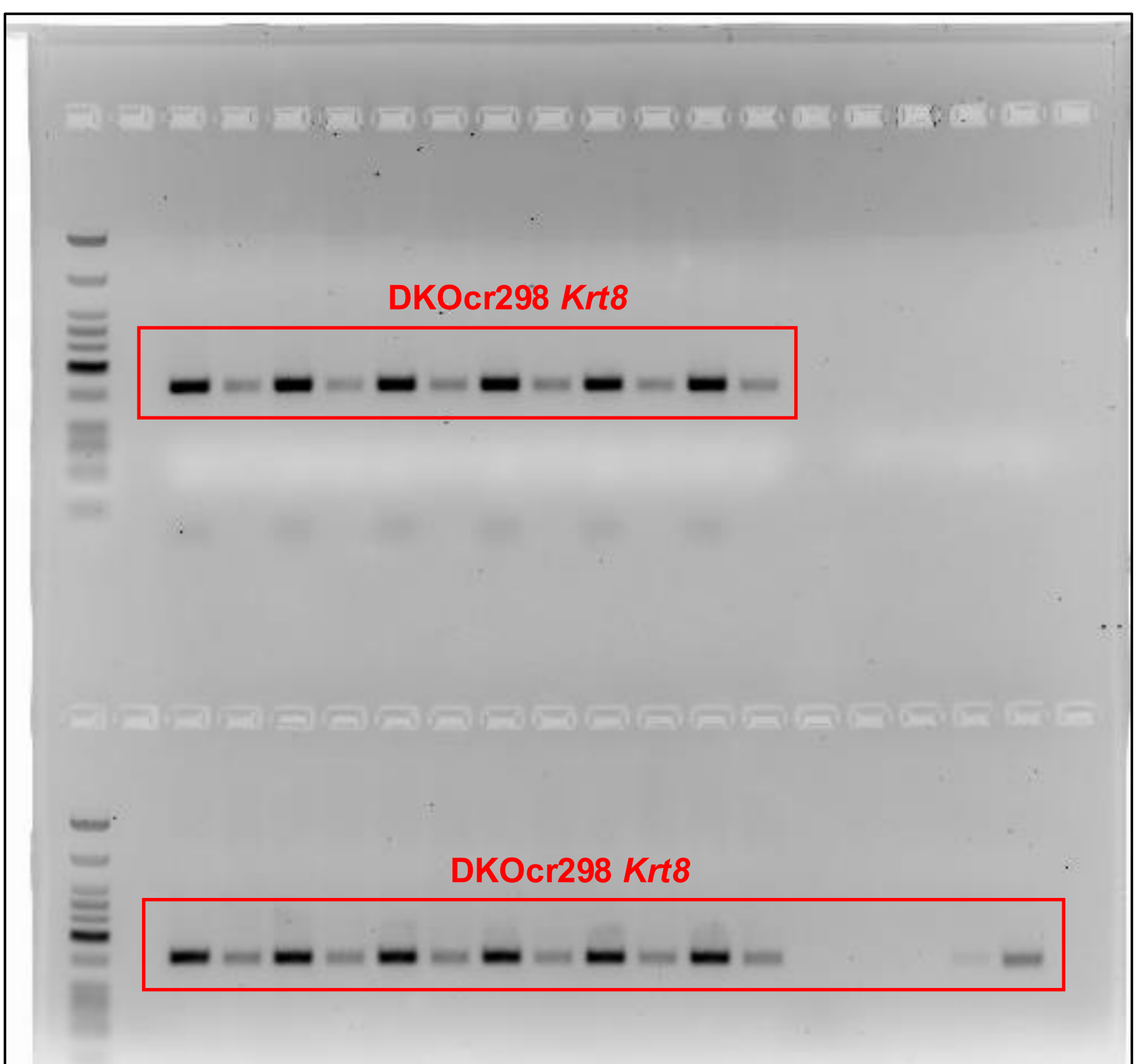

Full unedited gel for S.Figure 11C

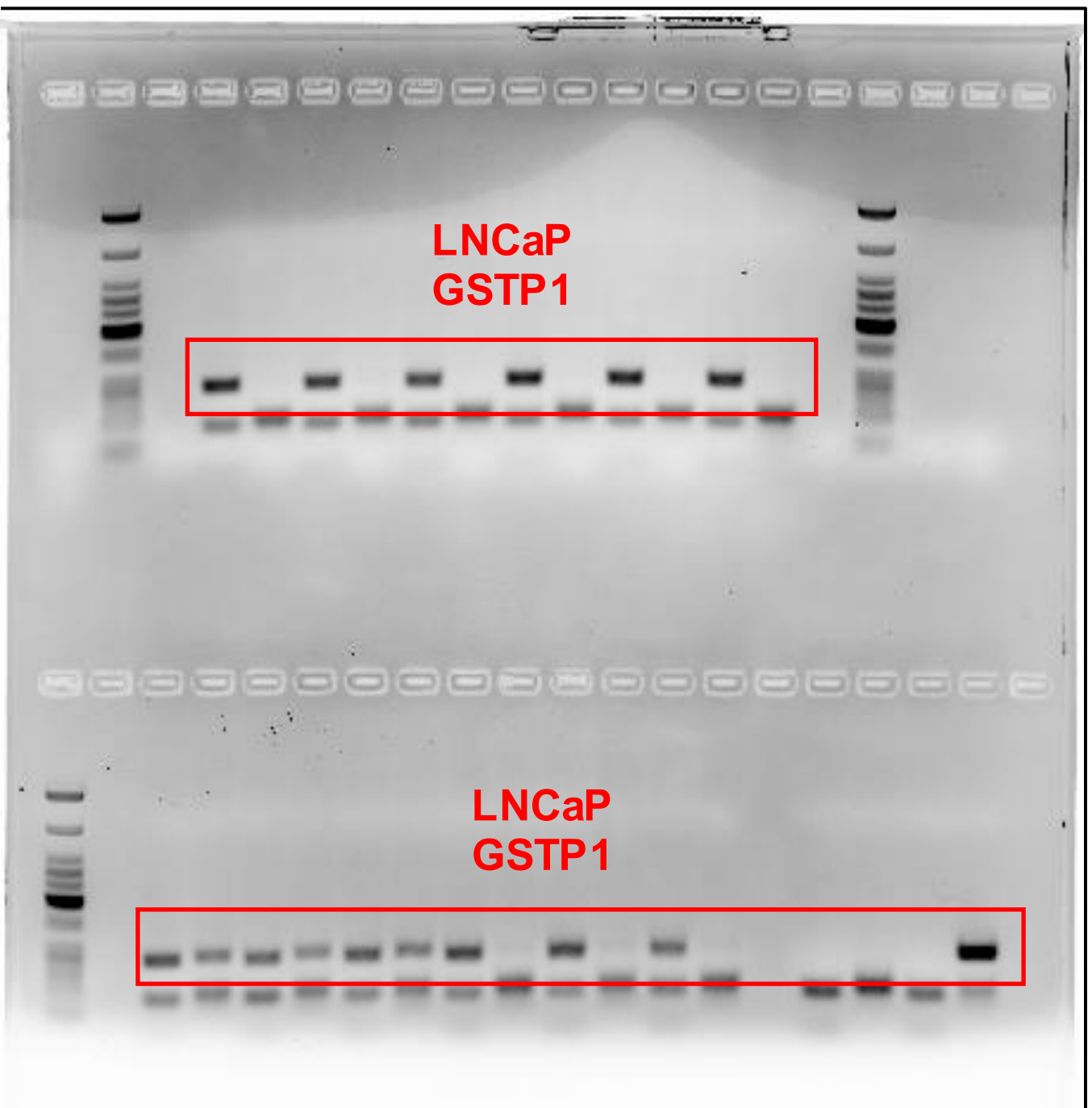

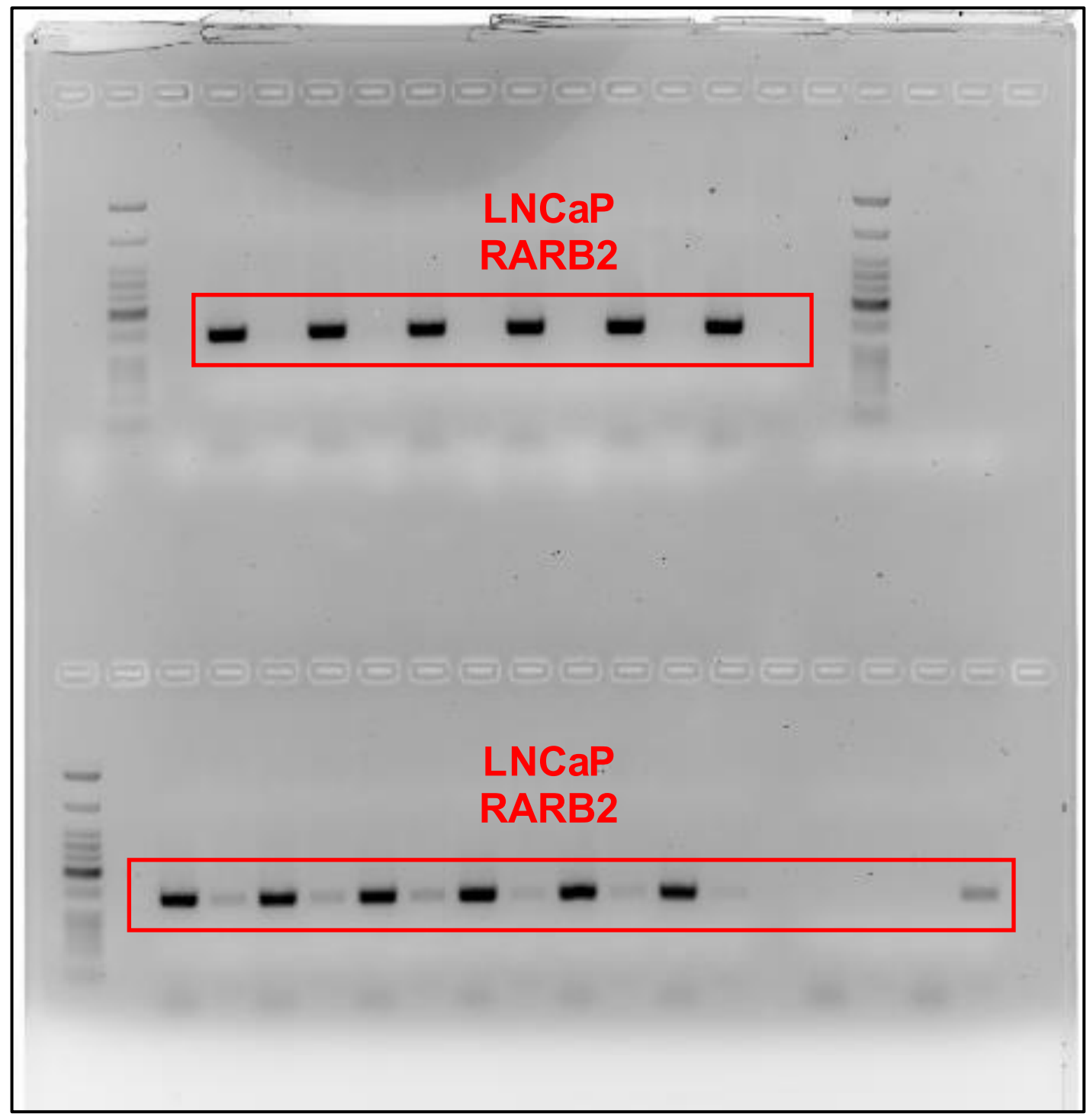

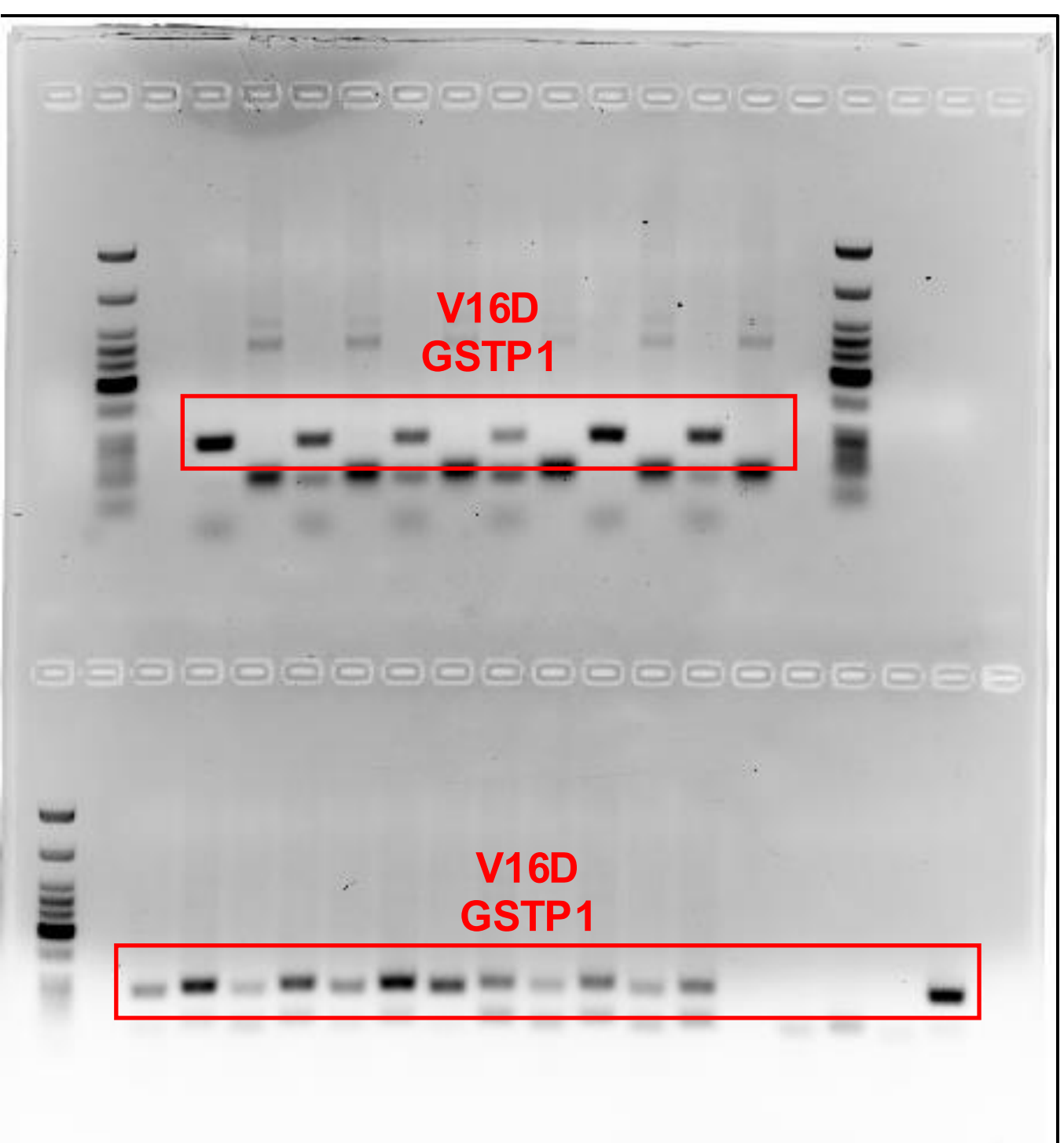

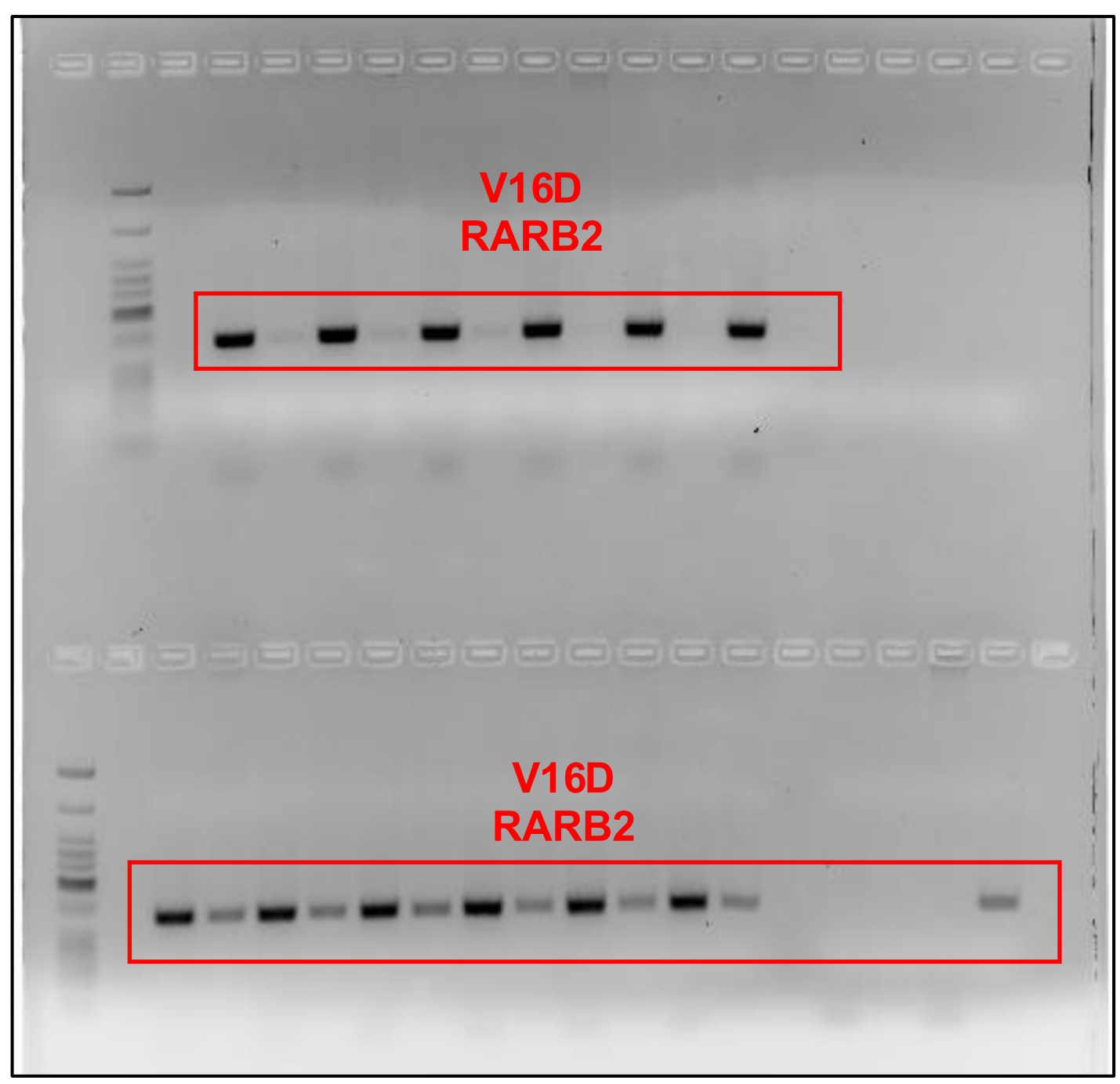

Full unedited gel for S.Figure 13F

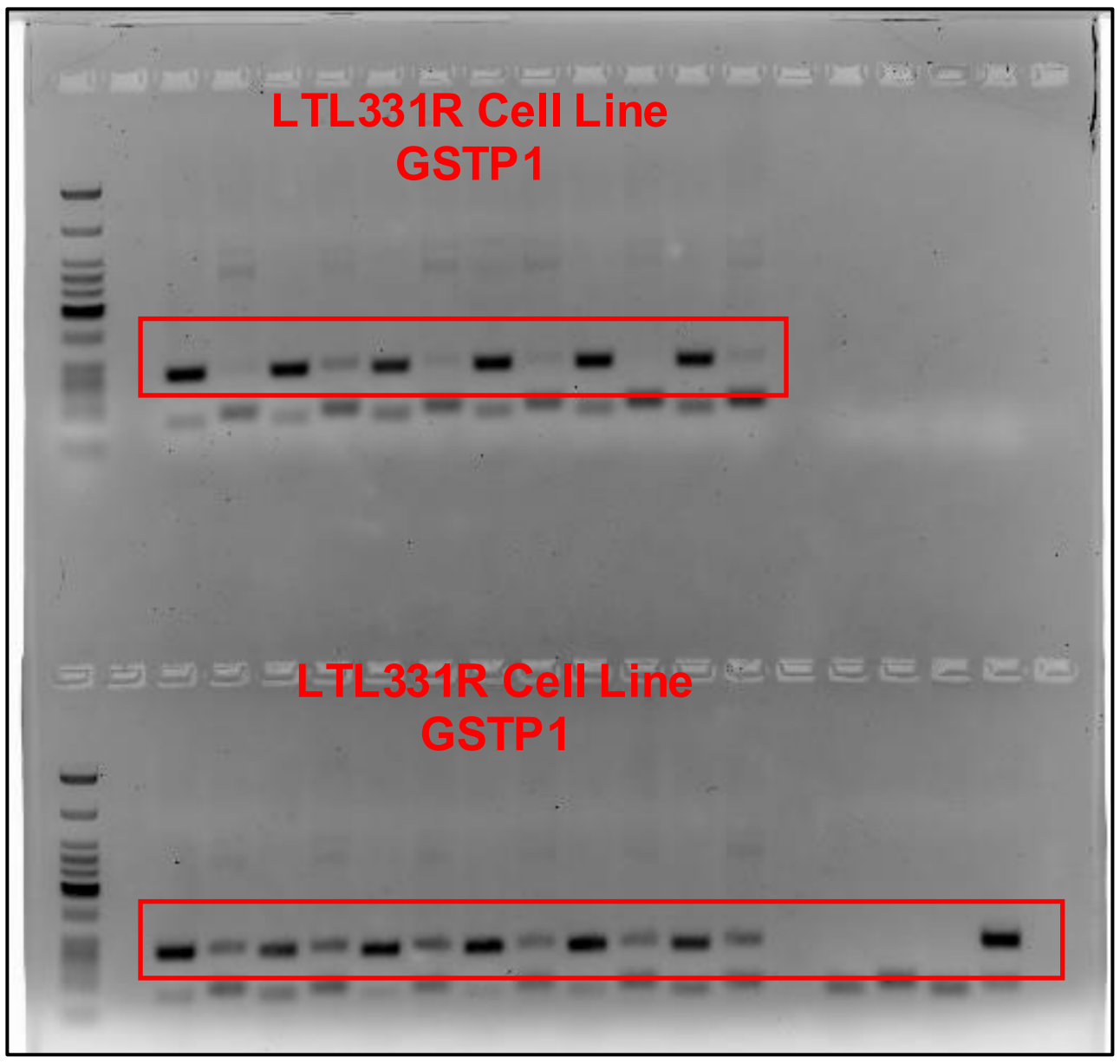

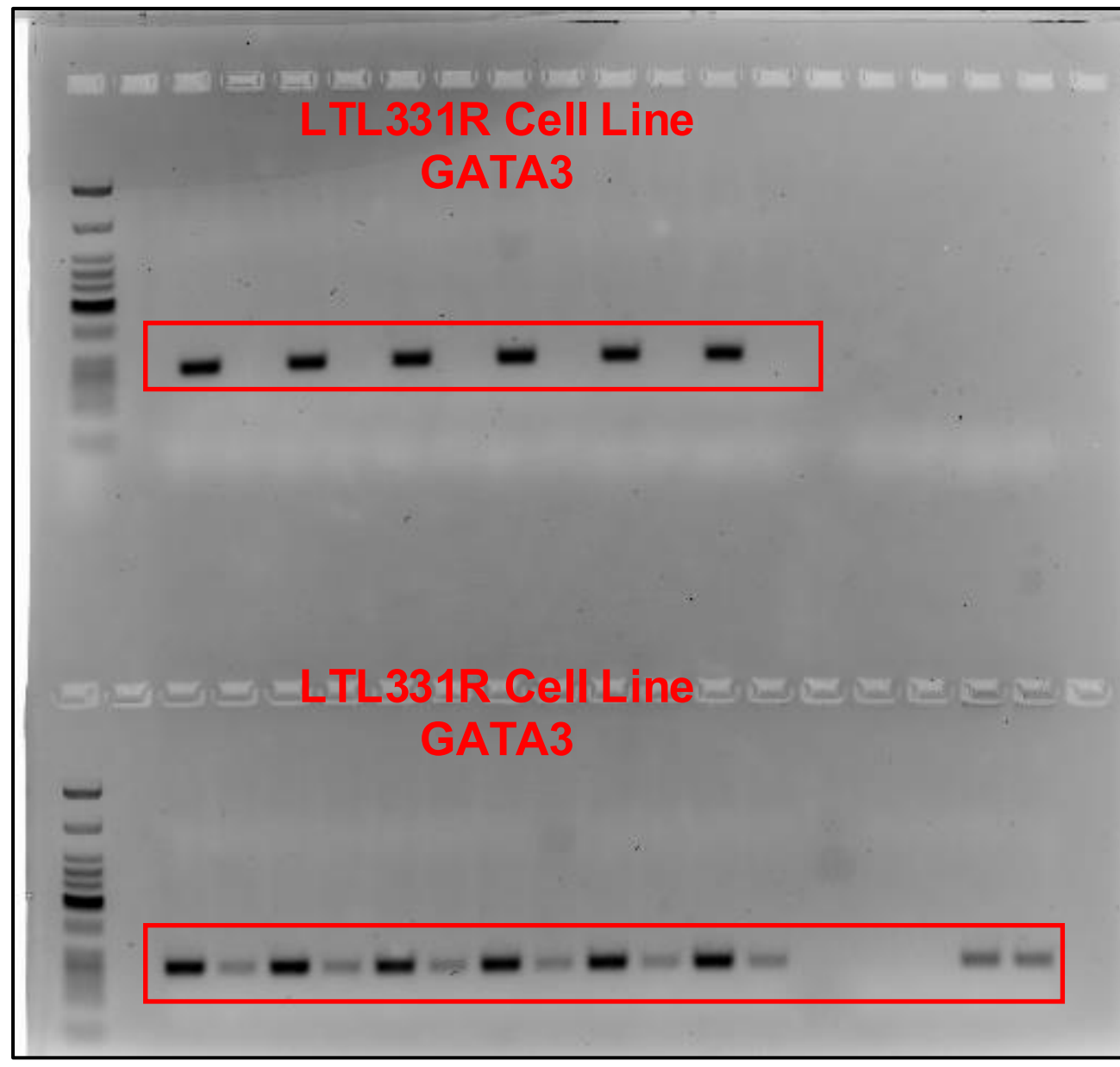

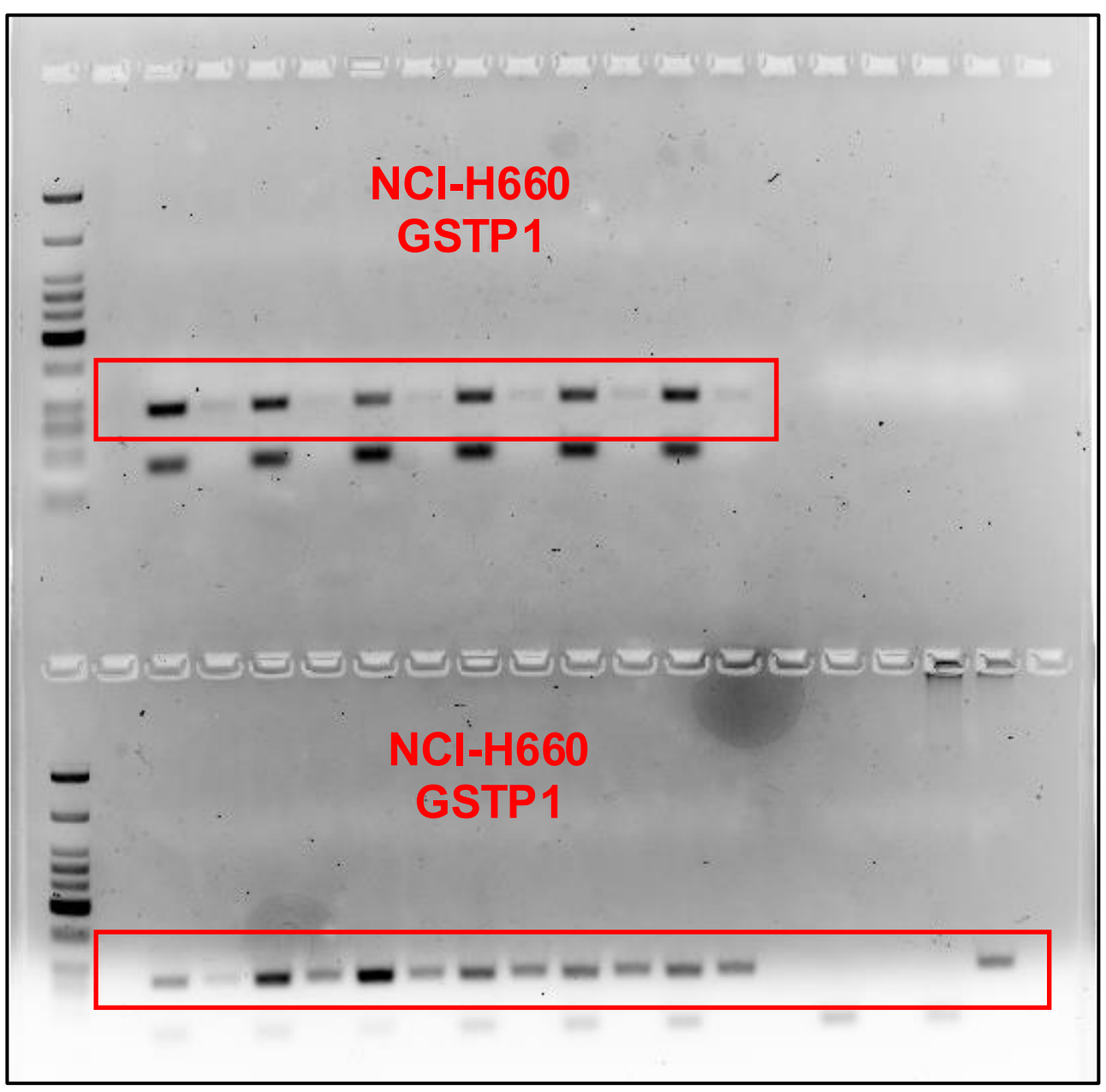

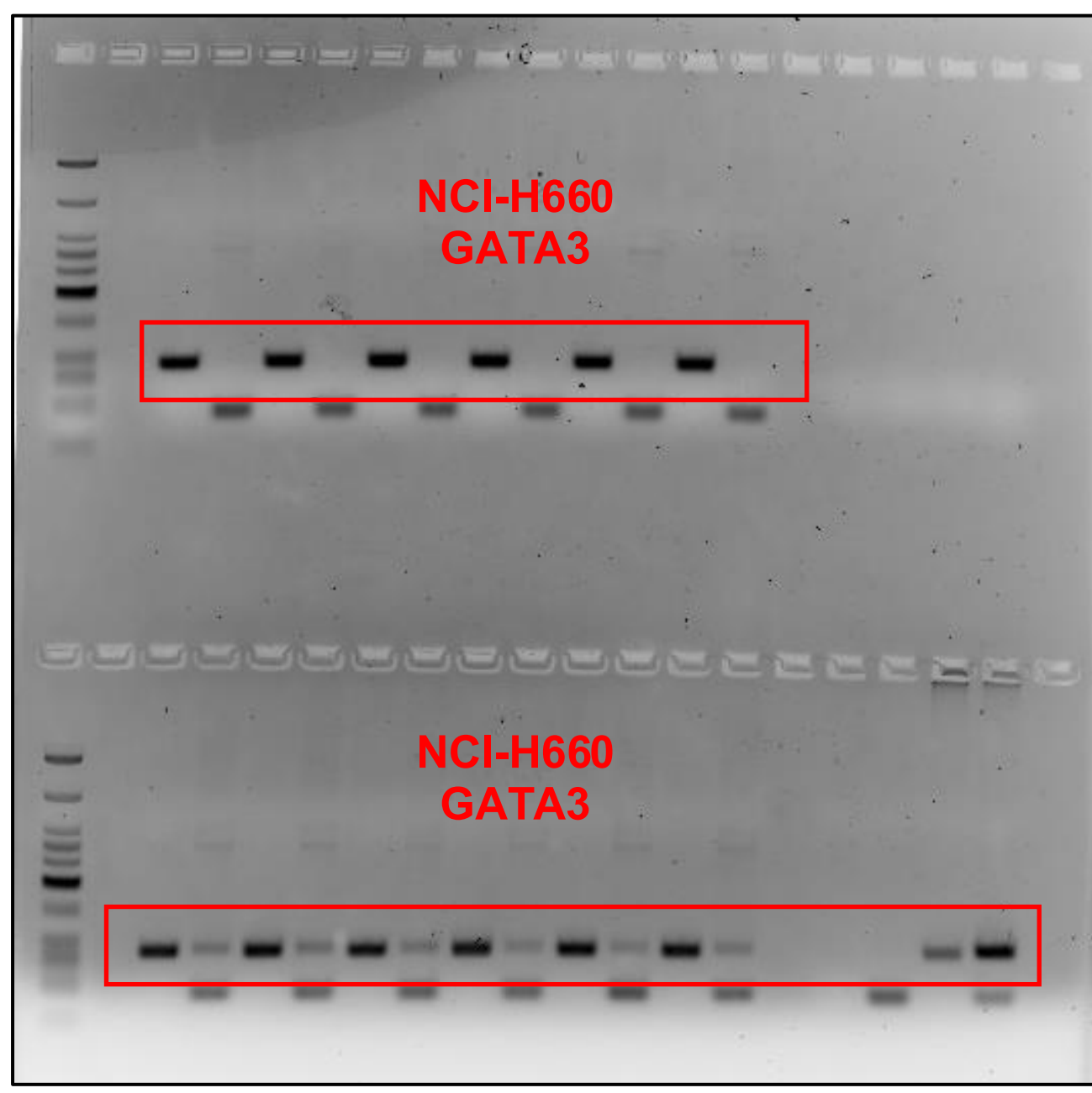

Full unedited gel for S.Figure 16D

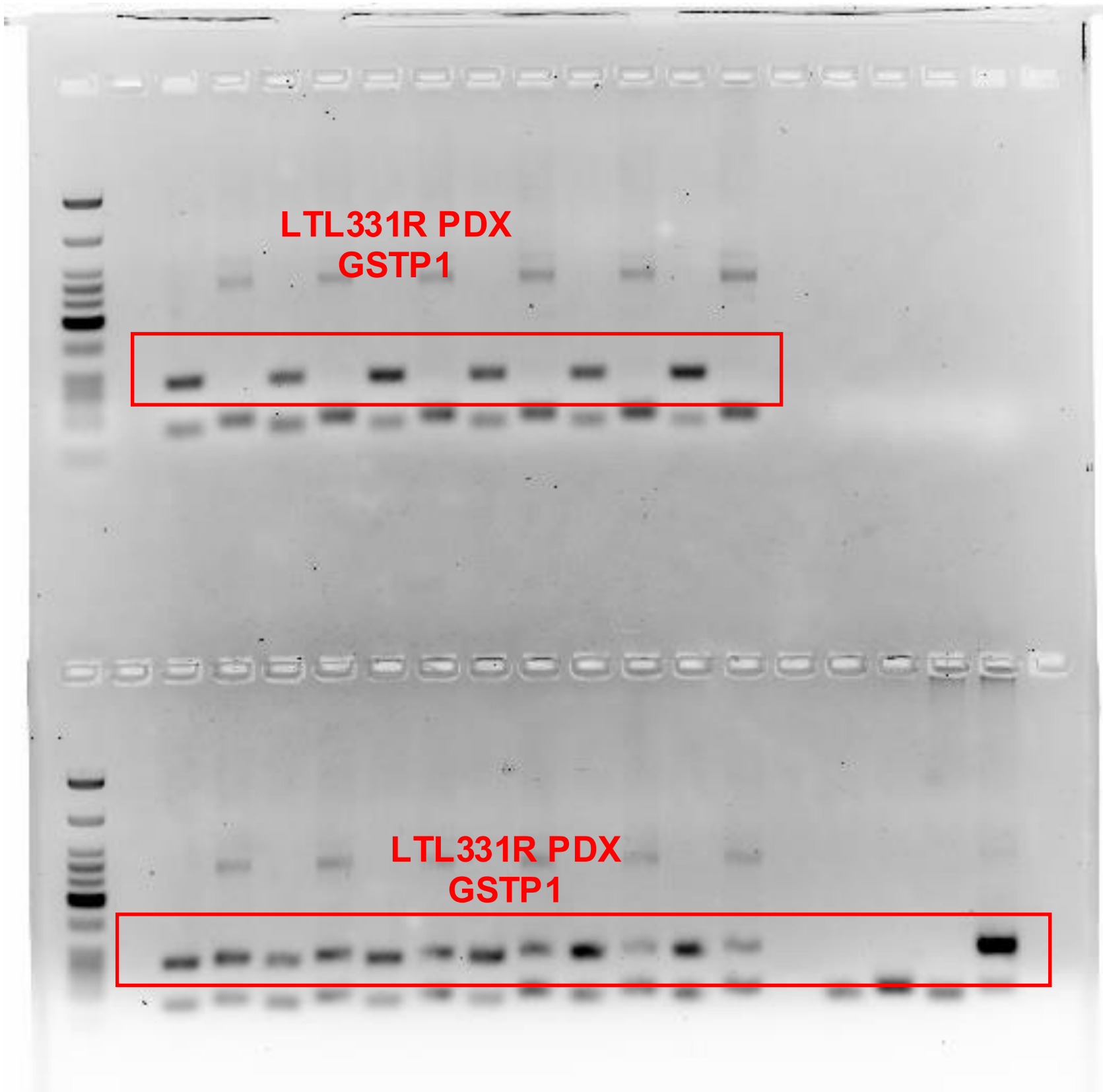

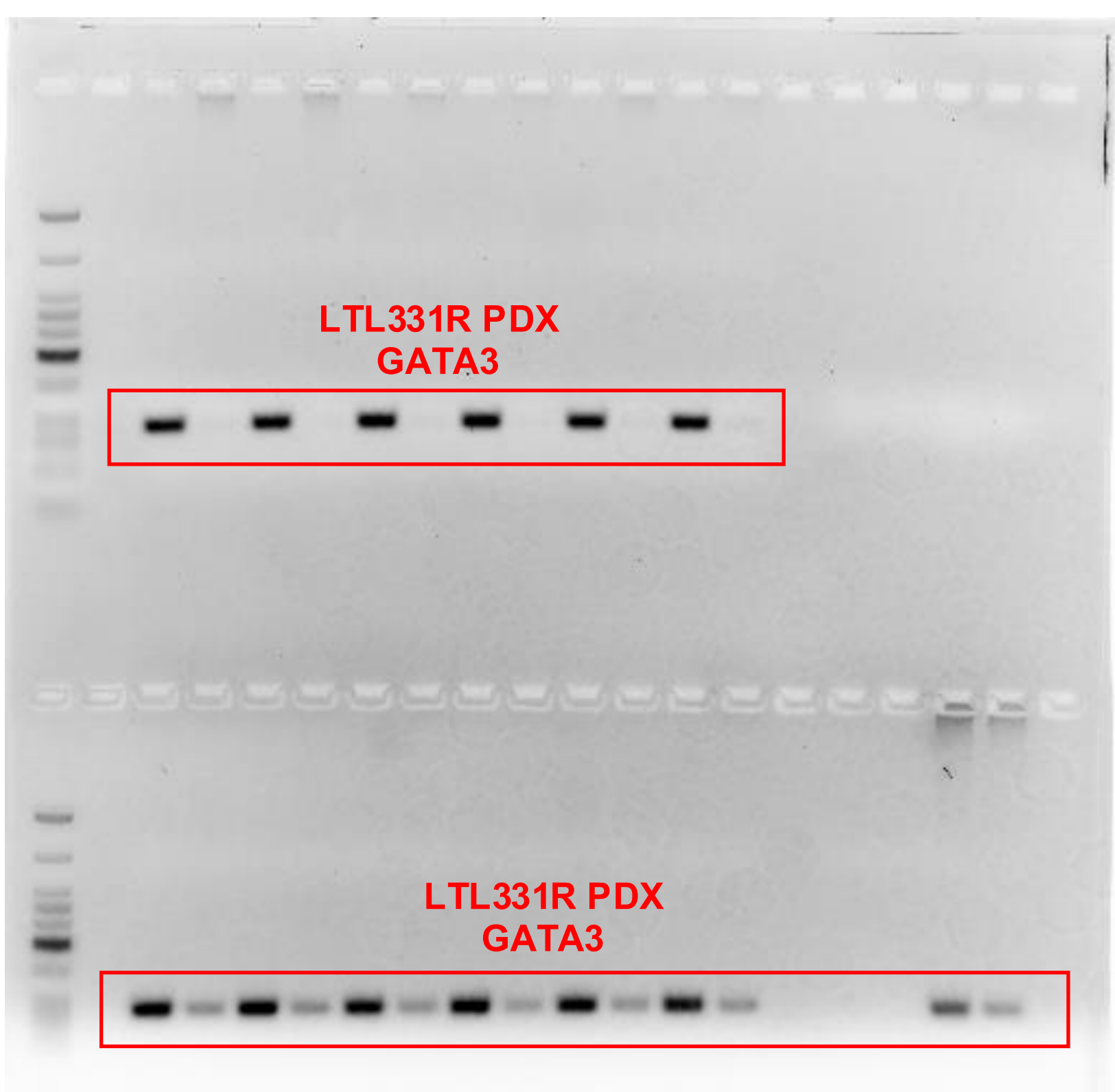

Supplement: Supplementary file 2 [file jciinsight-11-207543-s146.pdf]
